# Synthesis of Novel 1,4-Naphthoquinones Possessing Indole Scaffolds Using In(OTf)_3_ in Solvent-Free Conditions

**DOI:** 10.3390/molecules23081954

**Published:** 2018-08-06

**Authors:** Xiaojuan Yang, Liqiang Wu

**Affiliations:** 1 College of Chemistry and Chemical Engineering, Xinxiang University, Xinxiang 453003, Henan, China; yangxiaojuan2005@126.com; 2 School of Pharmacy, Xinxiang Medical University, Xinxiang 453003, Henan, China

**Keywords:** 1,4-naphthoquinone, substituted salicylic aldehydes, indoles, solvent-free, green synthesis

## Abstract

Novel 1,4-naphthoquinones possessing indole scaffolds were prepared by the reaction of 2-hydroxy-1,4-naphthoquinone-substituted salicylic aldehydes and indoles using In(OTf)_3_ as a catalyst. The method has the advantages of simple operation, mild reaction conditions, and friendly environment.

## 1. Introduction

Multi-component reactions, in which multiple reactants are combined to form a single, more complex system, have been used extensively to synthesize chemically and biologically important compounds [1]. This reaction provides a wide range of possibilities to efficiently construct highly complex molecules in one process, thus avoiding complex purification operations and saving solvents and reagents. In the past decade, the three component and four component reactions have undergone tremendous development [2].

**Figure 1:**
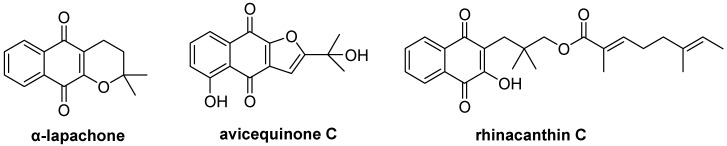
Structure of some natural 1,4-naphthoquinones.

Quinone skeletons are present in a broad range of natural products and synthetic molecules with important biological activities [3]. Among them, 1,4-naphthoquinone scaffolds have received considerable attention because of the synthetic challenges associated with their changeable molecular architecture and their interesting biological properties, such as their anticancer [4], antifungal [5], antiviral [6], and anti-inflammatory activities [7]. 1,4-Naphthoquinone skeleton can be found in a wide range of natural products, such as α-lapachone [8], rhinacanthin C [9], and avicequinone C [10] (Figure 1). In this context, the development of facile methods to access these new targets with structural diversity is very desirable and valuable for drug discovery.

Bioactive indole compounds are widely distributed in nature [11] and presented in the market [12] as active pharmaceutical ingredients [13,14]. Moreover, in modern times, indole derivatives are significant players in a diverse array of markets such as dyes, plastics, agriculture, vitamin supplements, over-the-counter drugs, flavor enhancers, and perfumery.

**Scheme 1:**
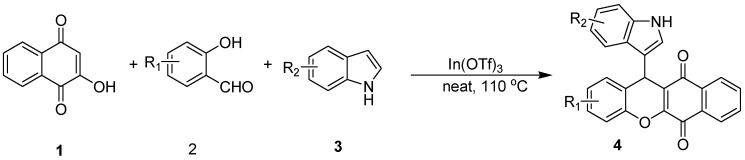
Synthesis of 1,4-naphthoquinones possessing indole scaffolds.

Herein, we describe a simple and efficient method for rapid preparation of 1,4-naphthoquinones possessing indole scaffolds using a catalytic amount of In(OTf)_3_ under solvent-free conditions (Scheme 1). As far as we know, it is probably the first example of synthesis 1,4-naphthoquinones possessing indole scaffolds using In(OTf)_3_ as catalyst under solvent-free conditions. As a result, 1,4-naphthoquinones-fused indoles, which combine two kinds of bioactive heterocyclic nuclei, are expected to become a research hotspot in pharmacology. Further studies to delineate the activities of the novel compounds are underway.

## 2. Results and Discussion

Initially, we began our studies by evaluating the reaction between 2-hydroxy-1,4-naphthoquinone, salicylic aldehyde, and 2-phenylindole using In(OTf)_3_ (20 mol %) as the catalyst in toluene as the solvent at 110 °C for 8 h, which provided the desired product **4a** in a 35% yield. Encouraged by this preliminary result, the screening of solvents and catalysts, as well as the in fluence of temperature and catalyst loading, was investigated to establish the optimized reaction conditions (Table 1). Solvent effects were first investigated (Table 1, compare entries 1–6). The neat reaction provided higher yields than those using other organic solvents (entries 2–6). To improve the yields, we then examined this reaction using different catalysts; the In(OTf)_3_ catalyst showed the best performance (entry 6). When this reaction was carried out without In(OTf)_3_ or in the presence of *p*-TsOH, H_2_SO_4_, FeCl_3_, Sc(OTf)_3_, or InCl_3,_ the product was obtained in low yield (Table 1, entries 7–12). The effect of the reaction temperature was investigated; it was observed that the reaction performed at 110 °C provided the best results (entries 6, 13–15). We also evaluated the amount of In(OTf)_3_ required for the reaction; the results from Table 1 (entries 6, 16–18) show that 10 mol % In(OTf)_3_ at 110 °C under solvent-free conditions is optimal for the reaction.

Under optimal conditions, we then developed the design and diversity-oriented synthesis of novel 1,4-naphthoquinones possessing indole scaffolds. Different substituted salicylic aldehydes and indoles were applied to this reaction to access structural diversity of target molecules. As illustrated in Table 2, the method is suitable for a wide scope of substituted salicylic aldehydes and indoles. In all cases, the three component reactions are regioselective to 1,4-naphthoquinone derivatives **4**, and their structures were characterized by analytical and spectroscopic methods. For example, the infrared spectra of **4a** exhibited that the absorption of two C=O bonds existed at 1650 and 1632 cm^−1^; the high resolution mass spectrum of **4a** showed the quasi ion peak ([M + Na]^+^) at *m*/*z* = 476.1262, which agreed with the 1:1:1 adduct of 2-phenylindole, salicylic aldehyde, and 2-hydroxy-1,4-naphthoquinone with the loss of two water molecules. The ^1^H NMR spectrum of **4a** displayed two singlets at *δ* = 5.82 ppm and *δ* = 11.39 ppm, which belong to CH group of C-12 position and NH protons, respectively. ^13^C NMR spectrum of **4a** exhibited 29 distinct resonances. Among them, three characteristic signals at *δ* = 29.5 ppm (arising from the Ar–CH group), 183.9, and 178.2 ppm (due to the two nonequivalent carbonyl groups) were shown.

**Scheme 2:**
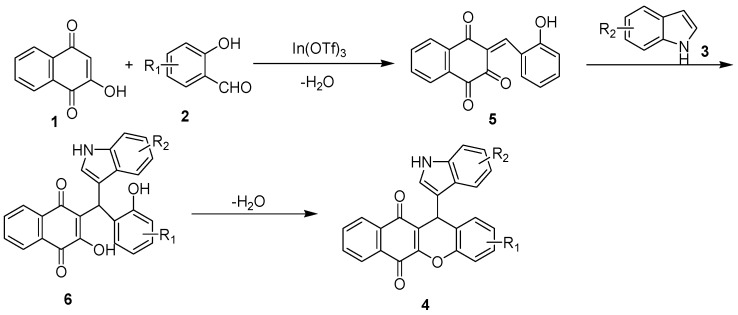
A suggested pathway for the formation of 1,4-naphthoquinones-possessing indole scaffolds.

In Scheme 2, we show the suggested ways to form the hybrid. It is possible that lawone initially reacts with substituted salicylic aldehyde 2 to form olefin **5**, which goes through a nucleophilic addition of indole to form the Mannich-type intermediate **6**. This step is then followed by an intramolecular dehydration to yield to product **4**.

## 3. Materials and Methods

### 3.1. General Information

Melting points were determined on a XT-4 binocular microscope and were uncorrected. NMR spectra were determined on Bruker AV-400 spectrometer at room temperature using TMS as internal standard. IR spectra were determined on FTS-40 infrared spectrometer. High resolution mass spectra were recorded on a bruker micrOTOF-QIII mass spectrometer. Commercially available reagents were used throughout without further purification, unless otherwise stated.

### 3.2. General Procedure for the Synthesis of Compounds **4**

To a mixture of indole (1 mmol), substituted salicylic aldehyde (1 mmol) and 2-hydroxy-1,4-naphthoquinone (1 mmol), In(OTf)_3_ (0.1 mmol) was added. The mixture was stirred at 110 °C for 5–7 h. After completion of the reaction (TLC), the reaction mixture is cooled to room temperature, treated with water (10 mL), extracted with CH_2_Cl_2_ (2 × 10 mL), and filtered, and the solvent is evaporated in vacuo. Solvent was evaporated and the crude product purified by silica gel column chromatography using petroleum ether: dichloromethane (v:v = 1:3) as eluent to afford the pure product 4 (the copy of IR, ^1^H NMR, ^13^C NMR, and HMRS of compounds 4 see Supplementary Materials).

*12-(2-Phenyl-1H-indol-3-yl)-12H-benzo[b]xanthene-6,11-dione* (**4a**): Reddish-brown powder, m.p. 260–262 °C, ^1^H NMR (400 MHz,DMSO-*d*_6_) *δ*: 11.39 (s, 1H), 8.07–8.00 (m, 3H), 7.90–7.80 (m, 3H), 7.59 (t, 2H, *J* = 7.6 Hz), 7.49 (t, 1H, *J* = 7.2 Hz), 7.31–7.28 (m, 4H), 7.23–7.21 (m, 2H), 7.19 (t, 2H, *J* = 1.2 Hz), 5.82 (s, 1H); ^13^C NMR (100 MHz, DMSO-*d*6) *δ*: 183.9, 178.2, 150.9, 148.2, 136.3, 135.3, 135.2, 134.4, 133.4, 131.8, 130.7, 129.8, 129.6 (2C), 129.2 (2C), 128.7, 128.6, 126.9, 126.5, 126.3, 126.1, 124.1, 121.8, 121.3, 119.8, 118.1, 117.2, 115.2, 112.0, 29.5; IR (KBr): *v* 3360, 1632, 1650 cm^−1^; HRMS-ESI (*m*/*z*): calcd for C_31_H_19_NNaO_3_ [M + Na]^+^: 476.1263, found: 476.1262.

*2-Bromo-12-(2-Phenyl-1H-indol-3-yl)-12H-benzo[b]xanthene-6,11-dione* (**4b**): Red powder, m.p. 279–280 °C, ^1^H NMR (400 MHz,DMSO-*d*_6_) *δ*: 11.46 (s, 1H), 8.04–8.02 (m, 1H), 7.93–7.78 (m, 5H), 7.58 (t, *J* = 7.6 Hz, 2H), 7.48 (t, *J* = 7.2 Hz, 1H), 7.37–7.32 (m, 3H), 7.24 (d, *J* = 8.8 Hz ,1H), 7.04 (t, *J* = 7.6 Hz ,1H), 6.92–6.89 (m, 2H), 5.79 (s, 1H); ^13^C NMR (100 MHz, DMSO-*d*6) *δ*: 183.6, 177.8, 150.5, 147.3, 136.2, 135.6, 135.2, 134.5, 133.2, 132.0, 131.6 (2C), 130.6, 129.5 (2C), 129.3 (2C), 128.7, 126.8, 126.6, 126.5, 126.3, 122.0, 120.9, 120.0, 119.6, 118.0, 117.4, 114.8, 112.2, 19.4; IR (KBr): *v* 3379, 1671, 1634 cm^−1^; HRMS-ESI (*m*/*z*): calcd for C_31_H_18_BrNNaO_3_ [M + Na]^+^: 554.0368, found: 554.0362.

*2-Chloro-12-(2-Phenyl-1H-indol-3-yl)-12H-benzo[b]xanthene-6,11-dione* (**4c**): Red powder, m.p. 275-276 °C, ^1^H NMR (400 MHz,DMSO-*d*_6_) *δ*: 11.46 (s, 1H), 8.05–8.03 (m, 1H), 7.93–7.80 (m, 5H), 7.58 (t, *J* = 7.2 Hz, 2H), 7.49 (t, *J* = 7.2 Hz, 1H), 7.34–7.32 (m, 3H), 7.27–7.24 (m, 1H), 7.04 (d, *J* = 7.6 Hz, 1H), 6.92 (t, *J* = 7.6 Hz ,1H), 6.77 (d, *J* = 2.0 Hz ,1H), 6.92–6.89 (m, 2H), 5.81 (s, 1H); ^13^C NMR (100 MHz, DMSO-*d*6) *δ*: 183.6, 177.9, 150.6, 146.9, 136.2, 135.6, 135.2, 134.5, 133.2, 131.7, 130.7, 129.5 (2C), 129.4 (2C), 129.3, 129.0, 128.8, 128.7, 126.8, 126.5, 126.3, 126.2, 122.0, 120.8, 120.0, 119.3, 118.0, 114.8, 112.2, 29.6; IR (KBr): *v* 3377, 1675, 1634 cm^−1^; HRMS-ESI (*m*/*z*): calcd for C_31_H_18_ClNNaO_3_ [M + Na]^+^: 510.0873, found: 510.0858.

*4-Bromo-2-chloro-12-(2-Phenyl-1H-indol-3-yl)-12H-benzo[b]xanthene-6,11-dione* (**4d**): Red powder, m.p. 243–244 °C, ^1^H NMR (400 MHz,DMSO-*d*_6_) *δ*: 11.49 (s, 1H), 8.07–8.04 (m, 1H), 7.90–7.80 (m, 5H), 7.67 (d, *J* = 2.4 Hz, 1H), 7.57–7.46 (m, 3H), 7.39–7.32 (m, 2H), 7.08–7.04 (m, 1H), 6.97–6.93 (m, 1H), 6.78 (d, *J* = 2.4 Hz ,1H), 5.85 (s, 1H); ^13^C NMR (100 MHz, DMSO-*d*6) *δ*: 183.5, 177.4, 150.3, 144.2, 136.1, 136.0, 135.2, 134.6, 133.0, 131.6, 130.7, 129.6, 129.5 (2C), 129.2 (2C), 128.8, 128.5, 127.7, 127.4, 126.8, 126.5, 126.4, 122.1, 121.3, 120.1, 118.0, 114.6, 112.2, 112.0, 30.0; IR (KBr): *v* 3288, 1685, 1637 cm^−1^; HRMS-ESI (*m*/*z*): calcd for C_31_H_17_BrClNNaO_3_ [M + Na]^+^: 587.9978, found: 587.9968.

*2-Methyl-12-(2-Phenyl-1H-indol-3-yl)-12H-benzo[b]xanthene-6,11-dione* (**4e**): Orange-red powder, m.p. 244–245 °C, ^1^H NMR (400 MHz,DMSO-*d*_6_) *δ*: 11.38 (s, 1H), 8.03–7.99 (m, 3H), 7.86–776 (m, 3H), 7.60 (t, *J* = 7.6 Hz, 2H), 7.50 (t, *J* = 7.2 Hz, 1H), 7.32–7.26 (m, 2H), 7.13 (d, *J* = 8.0 Hz ,1H), 7.03–6.96 (m, 2H), 6.86 (t, *J* = 7.2 Hz, 2H), 6.63 (s, 3H), 5.72 (s, 1H); ^13^C NMR (100 MHz, DMSO-*d*6) *δ*: 183.7, 178.2, 150.9, 146.2, 136.3, 135.3, 135.1, 134.3, 133.5, 131.8, 130.7, 129.8, 129.5 (2C), 129.4 (2C), 129.2, 128.5, 126.9, 126.4, 126.3, 123.7, 121.8, 121.1, 119.7, 118.1, 117.0, 115.2, 112.0, 29.5, 20.8; IR (KBr): *v* 3372, 1677, 1631 cm^−1^; HRMS-ESI (*m*/*z*): calcd for C_32_H_21_NNaO_3_ [M + Na]^+^: 490.1419, found: 490.1410.

*2-Nitro-12-(2-Phenyl-1H-indol-3-yl)-12H-benzo[b]xanthene-6,11-dione* (**4f**): Yellow powder, m.p. 245–246 °C, ^1^H NMR (400 MHz, DMSO-*d*_6_) *δ*: 11.49 (s, 1H), 8.07–8.04 (m, 1H), 7.90–7.80 (m, 5H), 7.67 (d, *J* = 2.4 Hz, 1H), 7.57–7.46 (m, 3H), 7.39–7.32 (m, 2H), 7.08–7.04 (m, 1H), 6.97–6.93 (m, 1H), 6.78 (d *J* = 2.4 Hz, 1H), 5.85 (s, 1H); ^13^C NMR (100 MHz, DMSO-*d*6) *δ*: 183.6, 177.6, 152.3, 150.2, 144.7, 136.2, 136.0, 135.3, 134.7, 133.0, 131.6, 130.7, 129.5 (2C), 129.3 (2C), 128.9, 126.8, 126.6, 126.4, 125.7, 125.5, 124.4, 122.1, 121.3, 120.1, 118.7, 118.1, 114.9, 112.2, 29.6; IR (KBr): *v* 3358, 1680, 1665, 1644 cm^−1^; HRMS-ESI (*m*/*z*): calcd for C_31_H_18_N_2_NaO_5_ [M + Na]^+^: 521.113, found: 521.1106.

*2-Methoxy-12-(2-Phenyl-1H-indol-3-yl)-12H-benzo[b]xanthene-6,11-dione* (**4g**): Yellow powder, m.p. 211–212 °C, ^1^H NMR (400 MHz,DMSO-*d*_6_) *δ*: 11.35 (s, 1H), 8.06–7.98 (m, 3H), 7.88–7.78 (m, 3H), 7.59 (d, *J* = 7.6 Hz, 2H), 7.48 (d, *J* = 7.6 Hz, 1H), 7.31–7.26 (m, 2H), 7.02–6.98 (m, 1H), 6.88–6.83 (m, 2H), 6.73 (d *J* = 7.4 Hz, 1H), 6.60–6.58 (m, 1H), 5.72 (s, 1H), 3.71 (s, 3H); ^13^C NMR (100 MHz, DMSO-*d*6) *δ*: 183.8, 178.2, 159.4, 150.9, 148.7, 136.3, 135.2, 135.1, 134.4, 133.5, 131.8, 130.7, 130.3, 129.5 (2C), 129.2 (2C), 128.5, 126.9, 126.4, 126.3, 121.8, 121.6, 119.7, 118.2, 115.9, 115.4, 113.2, 112.0, 101.7, 55.9, 29.0; IR (KBr): *v* 3326, 1682, 1638 cm^−1^; HRMS-ESI (*m*/*z*): calcd for C_32_H_21_NNaO_4_ [M + Na]^+^: 506.1368, found: 506.1358.

*2,4-Dibromo-12-(2-phenyl-1H-indol-3-yl)-12H-benzo[b]xanthene-6,11-dione* (**4h**): Red powder, m.p. 252–253 °C, ^1^H NMR (400 MHz,DMSO-*d*_6_) *δ*: 11.50 (s, 1H), 8.06 (dd, *J* = 2.0, 7.6 Hz, 1H), 7.88–7.82 (m, 5H), 7.77 (d, *J* = 2.4 Hz, 1H), 7.57–7.47 (m, 3H), 7.39–7.33 (m, 2H), 7.08–7.04 (m, 1H), 6.96 (t, *J* = 7.6 Hz, 1H), 6.90 (d, *J* = 7.0 Hz, 1H), 5.85 (s, 1H); ^13^C NMR (100 MHz, DMSO-*d*6) *δ*: 183.5, 177.4, 150.3, 144.6, 136.1, 136.0, 135.2, 134.6, 134.2, 133.0, 131.6, 131.5, 130.7, 129.5 (2C), 129.3 (2C), 128.8, 128.1, 126.8, 126.5, 126.4, 122.1, 121.4, 120.1, 118.0, 117.3, 114.7, 112.2, 112.1, 29.9; IR (KBr): *v* 3289, 1683, 1635 cm^−1^; HRMS-ESI (*m*/*z*): calcd for C_31_H_17_Br_2_NNaO_3_ [M + Na]^+^: 631.9473, found: 631.9462.

*3-Methoxy-12-(2-phenyl-1H-indol-3-yl)-12H-benzo[b]xanthene-6,11-dione* (**4i**): Orange-red powder, m.p. 244–245 °C, ^1^H NMR (400 MHz, DMSO-*d*_6_) *δ*: 11.40 (s, 1H), 8.05–8.00 (m, 3H), 7.88–7.78 (m, 3H), 7.61 (d, *J* = 7.6 Hz, 2H), 7.50 (t, *J* = 7.6 Hz, 1H), 7.32–7.20 (m, 3H), 7.03–6.99 (m, 1H), 6.88–6.84 (m, 1H), 6.77 (dd, *J* = 2.8, 8.8 Hz, 1H), 5.75 (s, 1H), 3.51 (s, 1H); ^13^C NMR (100 MHz, DMSO-*d*6) *δ*: 183.8, 178.2, 156.8, 151.2, 142.1, 136.3, 135.3, 135.1, 134.3, 133.4, 131.8, 130.7, 129.6 (2C), 129.3 (2C), 128.6, 126.8, 126.4, 126.3, 125.1, 121.8, 120.2, 119.7, 118.3, 118.1, 115.0, 114.1, 114.0, 112.1, 55.5, 29.9; IR (KBr): *v* 3304, 1683, 1638 cm^−1^; HRMS-ESI (*m*/*z*): calcd for C_32_H_21_NNaO_4_ [M + Na]^+^: 506.1368, found: 506.1357.

*4-Bromo-2-chloro-12-(1H-indol-3-yl)-12H-benzo[b]xanthene-6,11-dione* (**4j**): Yellow-brown powder, m.p. 248–250 °C, ^1^H NMR (400 MHz, DMSO-*d*_6_) *δ*: 11.08 (s, 1H), 8.10 (d, *J* = 7.2 Hz, 1H), 7.92–7.84 (m, 3H), 7.73 (s, 1H), 7.68 (d, *J* = 8.0 Hz, 1H), 7.52 (s, 1H), 7.38 (s, 1H), 7.33–7.31 (d, *J* = 7.6 Hz, 1H), 7.08–7.00 (m, 2H), 5.71 (s, 1H); ^13^C NMR (100 MHz, DMSO-*d*_6_) *δ*: 183.2, 177.7, 150.5, 144.9, 136.7, 135.0, 134.6, 131.7, 131.3, 131.2, 129.6, 129.0, 128.6, 126.6, 126.2, 125.5, 124.9, 122.1, 121.8, 119.7, 118.5, 118.2, 112.3, 111.8, 29.9; IR (KBr): *v* 3403, 1633, 1680 cm^−1^; HRMS-ESI (*m*/*z*): calcd for C_25_H_13_BrClNNaO_3_ [M + Na]^+^: 511.9665, found: 511.9664.

*2-chloro-12-(1H-indol-3-yl)-12H-benzo[b]xanthene-6,11-dione* (**4k**): Brown powder, m.p. 343–345 °C, ^1^H NMR (400 MHz, DMSO-*d*_6_) *δ*: 11.04 (s, 1H), 7.91–7.89 (m, 1H), 7.85–7.82 (m, 3H), 7.61 (d, *J* = 8.0 Hz, 1H), 7.46–7.30 (m, 5H), 7.06–6.96 (m, 2H), 5.67 (s, 1H); ^13^C NMR (100 MHz, DMSO-*d*_6_) *δ*: 183.4, 178.2, 150.5, 147.6, 136.7, 135.0, 134.4, 131.8, 131.1, 129.5, 129.4, 128.5, 127.1, 126.5, 126.2, 125.6, 124.6, 121.7, 119.5, 119.3, 118.5 (2C), 112.3, 29.5; IR (KBr): *v* 3410, 1634, 1685 cm^−1^; HRMS-ESI (*m*/*z*): calcd for C_25_H_14_ClNNaO_3_ [M + Na]^+^: 434.0560, found: 434.0559.

*2-Bromo-12-(1H-indol-3-yl)-12H-benzo[b]xanthene-6,11-dione* (**4l**): Reddish-brown powder, m.p. 262–264 °C, ^1^H NMR (400 MHz, DMSO-*d*_6_) *δ*: 8.10–8.07 (m, 1H), 7.91–7.82 (m, 3H), 7.61–7.58 (m, 2H), 7.46–7.43 (m, 1H), 7.36–7.30 (m, 3H), 7.04 (t, *J* = 7.2 Hz, 1H), 6.98 (t, *J* = 7.6 Hz, 1H); ^13^C NMR (100 MHz, DMSO-*d*_6_) *δ*: 183.3, 178.2, 150.5, 148.1, 136.7, 135.0, 134.4, 132.5, 131.8, 131.4, 131.1, 127.5, 126.5, 126.2, 125.6, 124.6, 121.8, 121.7, 119.7, 119.5 (2C), 118.5, 117.4, 112.3, 29.4; IR (KBr): *v* 3410, 1633, 1683 cm^−1^; HRMS-ESI (*m*/*z*): calcd for C_25_H_14_BrNNaO_3_ [M + Na]^+^: 478.0055, found: 478.0056.

*2-Fluoro-12-(1H-indol-3-yl)-12H-benzo[b]xanthene-6,11-dione* (**4m**): Reddish-brown powder, m.p. 330–332 °C, ^1^H NMR (400 MHz, DMSO-*d*_6_) *δ*: 11.02 (s, 1H), 8.10–8.08 (m, 1H), 7.92–7.82 (m, 3H), 7.61 (d, *J* = 8.8 Hz, 1H), 7.43–7.40 (m, 1H), 7.35–7.22 (m, 3H), 7.15–7.10 (m, 1H), 7.04 (t, *J* = 7.2 Hz, 1H), 6.97 (t, *J* = 6.8 Hz, 1H), 5.66 (s, 1H); ^13^C NMR (100 MHz, DMSO-*d*_6_) *δ*: 183.4, 178.4, 160.6, 150.7, 145.2, 136.8, 135.0, 134.4, 131.8, 131.1, 126.5, 126.2, 125.6, 124.5, 121.6, 121.2, 119.5, 119.2, 119.1, 118.5, 118.4, 115.8, 115.7, 112.2, 29.8; IR (KBr): *v* 3416, 1650, 1638 cm^−1^; HRMS-ESI (*m*/*z*): calcd for C_25_H_14_FNNaO_3_ [M + Na]^+^: 418.0855, found: 418.0858.

*2-Chloro-12-(2-methyl-1H-indol-3-yl)-12H-benzo[b]xanthene-6,11-dione* (**4n**): Reddish-brown powder, m.p. 264–265 °C, ^1^H NMR (400 MHz, DMSO-*d*_6_) *δ*: 11.00 (s, 1H), 8.05–7.79 (m, 4H), 7.41 (d, *J* = 8.8 Hz, 1H), 7.33–7.30 (m, 1H), 7.19 (d, *J* = 7.6 Hz, 2H), 7.07 (d, *J* = 7.6 Hz, 1H), 6.88 (d, *J* = 7.2 Hz, 1H), 6.79 (d, *J* = 7.2 Hz, 1H), 5.57 (s, 1H), 2.63 (s, 3H); ^13^C NMR (100 MHz, DMSO-*d*_6_) *δ*: 183.4, 178.2, 149.9, 147.8, 135.5, 135.1, 134.5, 133.4, 131.7, 130.8, 129.9, 129.6, 128.7, 126.7, 126.5, 126.2, 121.1, 120.5, 119.3, 119.0, 116.9, 113.5, 111.4, 29.1, 12.1; IR (KBr): *v* 3379, 1679, 1660, 1635 cm^−1^; HRMS-ESI (*m*/*z*): calcd for C_26_H_16_ClNNaO_3_ [M + Na]^+^: 448.0716, found: 448.0707.

*2-Methyl-12-(2-methyl-1H-indol-3-yl)-12H-benzo[b]xanthene-6,11-dione* (**4o**): Reddish-brown powder, m.p. 269–270 °C, ^1^H NMR (400 MHz, DMSO-*d*_6_) *δ*: 10.92 (s, 1H), 8.01 (dd, *J* = 4.0, 7.2 Hz, 1H), 7.85–7.75 (m, 3H), 7.20–7.17 (m, 2H), 7.09–7.02 (m, 2H), 6.90–6.85 (m, 2H), 6.77 (t, *J* = 7.6 Hz, 1H), 5.44 (s, 1H), 2.59 (s, 3H), 2.15 (s, 3H); ^13^C NMR (100 MHz, DMSO-*d*_6_) *δ*: 183.5, 178.5, 150.2, 146.9, 135.4, 135.3, 135.0, 134.3, 132.9, 131.7, 130.8, 130.4, 129.3, 126.8, 126.4, 126.1, 123.6, 121.4, 120.4, 119.1, 117.1, 116.8, 114.1, 111.2, 29.1, 20.7, 12.1; IR (KBr): *v* 3375, 1673, 1632 cm^−1^; HRMS-ESI (*m*/*z*): calcd for C_27_H_19_NNaO_3_ [M + Na]^+^: 428.1263, found: 428.1263.

*12-(2-Methyl-1H-indol-3-yl)-12H-benzo[b]xanthene-6,11-dione* (**4p**): Yellow-brown powder, m.p. 350–352 °C, ^1^H NMR (400 MHz, DMSO-*d*_6_) *δ*: 10.94 (s, 1H), 8.05 (d, *J* = 4.4Hz, 1H), 7.86–7.80 (m, 3H), 7.35–7.24 (m, 2H), 7.18–7.07 (m, 4H), 6.87 (t, *J* = 7.2 Hz, 1H), 6.77 (t, *J* = 7.2 Hz, 1H) 5.55 (s, 1H); ^13^C NMR (100 MHz, DMSO-*d*_6_) *δ*: 183.5, 178.5, 150.2, 148.9, 135.5, 135.1, 134.4, 132.9, 131.7, 130.8, 130.5, 128.6, 126.8, 126.5, 126.2, 126.1, 124.0, 121.6, 120.4, 119.1, 117.1, 117.0, 114.1, 111.2, 29.0, 12.1; IR (KBr): *v* 3401, 2919, 1631, 1649 cm^−1^; HRMS-ESI (*m*/*z*): calcd for C_26_H_17_NNaO_3_ [M + Na]^+^: 414.1106, found: 414.1102.

*12-(5-Methoxy-1H-indol-3-yl)-12H-benzo[b]xanthene-6,11-dione* (**4q**): Yellow-brown powder, m.p. 260–261 °C, ^1^H NMR (400 MHz, DMSO-*d*_6_) *δ*: 10.82 (s, 1H), 8.10–8.08 (m, 1H), 7.93–7.83 (m, 3H), 7.42 (d, *J* = 7.2 Hz, 1H), 7.35 (d, *J* = 7.6Hz, 1H), 7.30–7.24 (m, 2H), 7.19–7.12 (m, 2H), 7.06 (d, *J* = 2.0 Hz, 1H), 6.70–6.67 (m, 1H), 5.61 (s, 1H), 3.72 (s, 3H); ^13^C NMR (100 MHz, DMSO-*d*_6_) *δ*: 183.5, 178.5, 153.7, 150.7, 149.0, 135.0, 134.4, 131.9, 131.1, 130.2, 128.6, 126.8, 126.5, 126.2, 126.0, 125.0, 124.9, 122.2, 118.7, 117.2, 112.8, 111.3, 100.7, 55.7, 29.5; IR (KBr): *v* 3442, 2926, 1631, 1654 cm^−1^; HRMS-ESI (*m*/*z*): calcd for C_26_H_17_NNaO_4_ [M + Na]^+^: 430.1055, found: 430.1062.

*12-(4-Methoxy-1H-indol-3-yl)-12H-benzo[b]xanthene-6,11-dione* (**4r**): Yellow-brown powder, m.p. 190–192 °C, ^1^H NMR (400 MHz, DMSO-*d*_6_) *δ*: 8.12–8.10 (m, 1H), 7.87–7.81 (m, 3H), 7.47 (d, *J* = 7.6 Hz, 1H), 7.27–7.16 (m, 3H), 7.09 (t, *J* = 6.4 Hz, 1H), 6.97 (t, *J* = 7.6 Hz, 1H), 6.89 (d, *J* = 8.0Hz, 1H), 6.51 (d, *J* = 7.6Hz, 1H), 5.91 (s, 1H), 3.94 (s, 3H); ^13^C NMR (100 MHz, DMSO-*d*_6_) *δ*: 183.6, 178.7, 154.2, 151.0, 148.4, 138.3, 135.0 (2C), 133.7, 132.4, 131.1, 130.1, 128.0, 126.4, 125.9, 123.4, 122.7, 120.1, 117.2, 115.7, 111.5, 105.5, 99.9, 55.4, 29.8; IR(KBr): *v* 3441, 2930, 1633, 1644 cm^−1^; HRMS-ESI (*m*/*z*): calcd for C_26_H_17_NNaO_4_ [M + Na]^+^: 430.1055, found: 430.1054.

*12-(5-Chloro-1H-indol-3-yl)-12H-benzo[b]xanthene-6,11-dione* (**4s**): Yellow-brown powder, m.p. 256–257 °C, ^1^H NMR (400 MHz, DMSO-*d*_6_) *δ*: 11.19 (s, 1H), 8.10–8.08 (m, 1H), 7.93–7.83 (m, 3H), 7.72 (d, *J* = 1.6 Hz, 1H), 7.16–7.12 (m, 5H), 7.06 (t, *J* = 2.0 Hz, 1H), 7.03 (d, *J* = 2.0 Hz, 1H), 5.66 (s, 1H); ^13^C NMR (100 MHz, DMSO-*d*_6_) *δ*: 183.5, 178.4, 150.8, 148.8, 135.1, 134.9, 134.4, 131.8, 131.2, 130.2, 128.7, 126.9, 126.5, 126.3, 126.2, 126.1, 124.9, 124.1, 122.1, 121.6, 119.3, 118.1, 117.3, 113.7, 29.1; IR(KBr): 3407, 2922, 1633, 1685 cm^−1^; HRMS-ESI (*m*/*z*): calcd for C_25_H_14_ClNNaO_3_ [M + Na]^+^: 434.0560, found: 434.0557.

## 4. Conclusions

A green and efficient procedure for the synthesis of 1,4-naphthoquinones possessing indole scaffolds was investigated via the three-component reaction of 2-hydroxy-1,4-naphthoquinone, substituted salicylic aldehydes, and indoles using In(OTf)_3_ as a catalyst. The method has the advantages of simple operation, mild reaction conditions, and a hospitable environment.

## Figures and Tables

**Table 1:** Reaction conditions optimization for the synthesis **4a**.

Entry	Solvent	Catalyst	Temperature/°C	Time/h	Yield/% ^1^
1	Toluene	In(OTf)_3_ (10 mol %)	110	8	35
2	DMF	In(OTf)_3_ (10 mol %)	110	8	39
3	EtOH	In(OTf)_3_ (10 mol %)	reflux	12	8
4	CH_3_CN	In(OTf)_3_ (10 mol %)	reflux	12	7
5	H_2_O	In(OTf)_3_ (10 mol %)	110	12	trace
6	-	In(OTf)_3_ (10 mol %)	110	6	53
7	-	-	110	10	11
8	-	*p*-TsOH (10 mol %)	110	8	25
9	-	H_2_SO_4_ (10 mol %)	110	8	8
10	-	FeCl_3_ (10 mol %)	110	8	29
11	-	Sc(OTf)_3_ (10 mol %)	110	8	36
12		InCl_3_ (10 mol %)	110	8	42
13	-	In(OTf)_3_ (10 mol %)	25	12	trace
14	-	In(OTf)_3_ (10 mol %)	100	6	43
15		In(OTf)_3_ (10 mol %)	120	6	51
16	-	In(OTf)_3_ (5 mol %)	110	6	40
17	-	In(OTf)_3_ (15 mol %)	110	6	49
18	-	In(OTf)_3_ (20 mol %)	110	6	53

^1^ Isolated yield.

**Table 2:** Preparation of 1,4-naphthoquinones possessing indole scaffolds.

Entry	R_1_	R_2_	Time/h	Product	Yield/%
1	H	2-Ph	6	**4a**	53
2	5-Br	2-Ph	5	**4b**	57
3	5-Cl	2-Ph	6	**4c**	50
4	3-Br-5-Cl	2-Ph	5	**4d**	53
5	5-Me	2-Ph	7	**4e**	48
6	5-NO_2_	2-Ph	7	**4f**	46
7	5-MeO	2-Ph	7	**4g**	42
8	3,5-Br_2_	2-Ph	5	**4h**	63
9	4-MeO	2-Ph	6	**4i**	55
10	3-Br-5-Cl	H	7	**4j**	41
10	5-Cl	H	7	**4k**	42
12	5-Br	H	7	**4l**	44
13	5-F	H	7	**4m**	47
14	5-Cl	2-Me	5	**4n**	59
15	5-Me	2-Me	5	**4o**	48
16	H	2-Me	5	**4p**	50
17	H	5-MeO	7	**4q**	40
18	H	4-MeO	7	**4r**	42
19	H	5-Cl	7	**4s**	43

## Supplementary Materials

The following are available online: IR, ^1^H NMR, ^13^C NMR, and HMRS of compounds **4.**
