# Supplementary material for: Synthesis of Novel 1,4-Naphthoquinones Possessing Indole Scaffolds Using In(OTf)3 in Solvent-Free Conditions
Source: Molecules. 2018 Aug 6;23(8):1954. doi: 10.3390/molecules23081954 (PMC6222671; doi:10.3390/molecules23081954)
Supplement: Supplementary file 1 [file molecules-23-01954-s001.pdf]

Synthesis of novel 1,4-naphthoquinones possessing indole scaffolds using $In(OTf)_3$ under solvent-free conditions

Xiaojuan Yang[1] and Liqiang Wu[2,*]

[1] College of Chemistry and Chemical Engineering, Xinxiang University, Xinxiang, Henan 453003, China; yangxiaojuan2005@126.com
[2] School of Pharmacy, Xinxiang Medical University, Xinxiang 453003, China; wliq1974@163.com
* Correspondence: wliq1974@163.com; Tel.: +86-373-302-9879

Content

Copy of IR, ^1H NMR, ^{13}C NMR and HRMS…………………………… 2-39

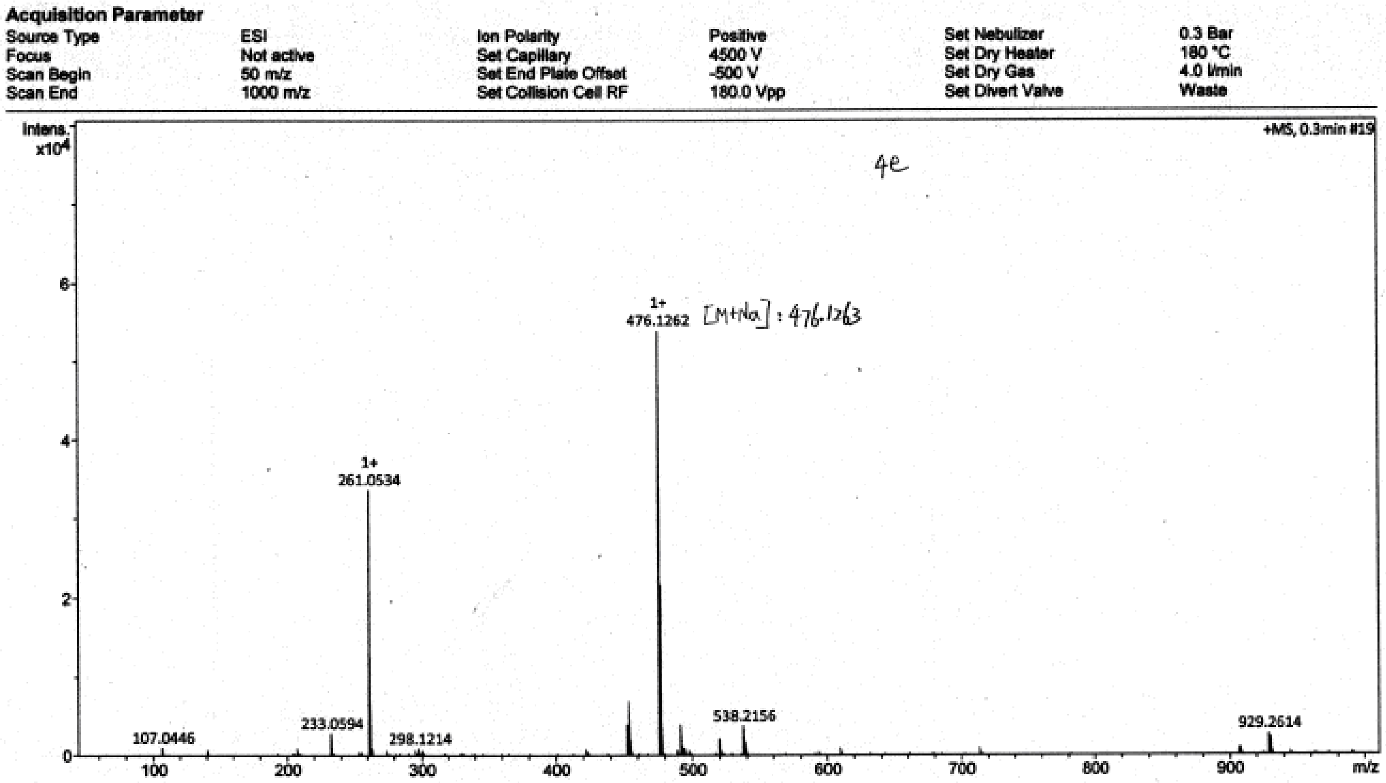

Figure S1 HRMS of **4a**

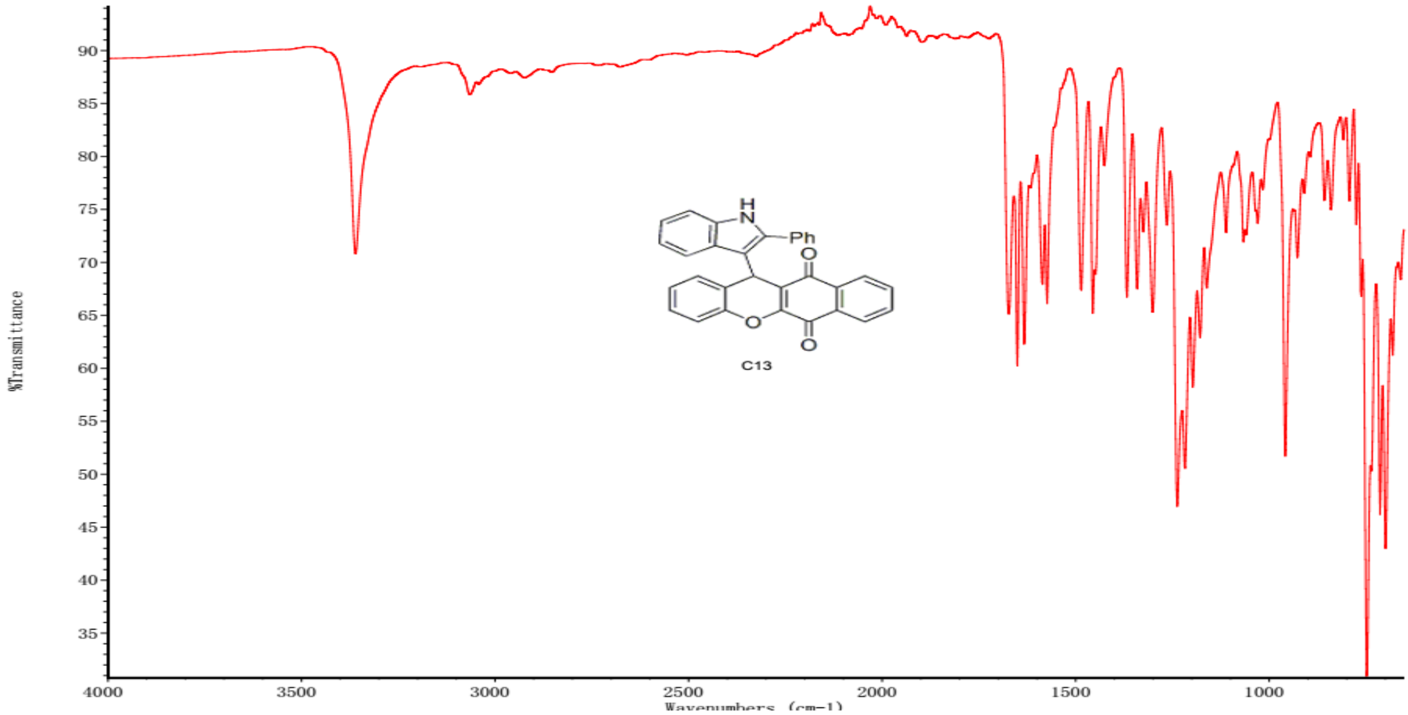

Figure S2 IR of **4a**

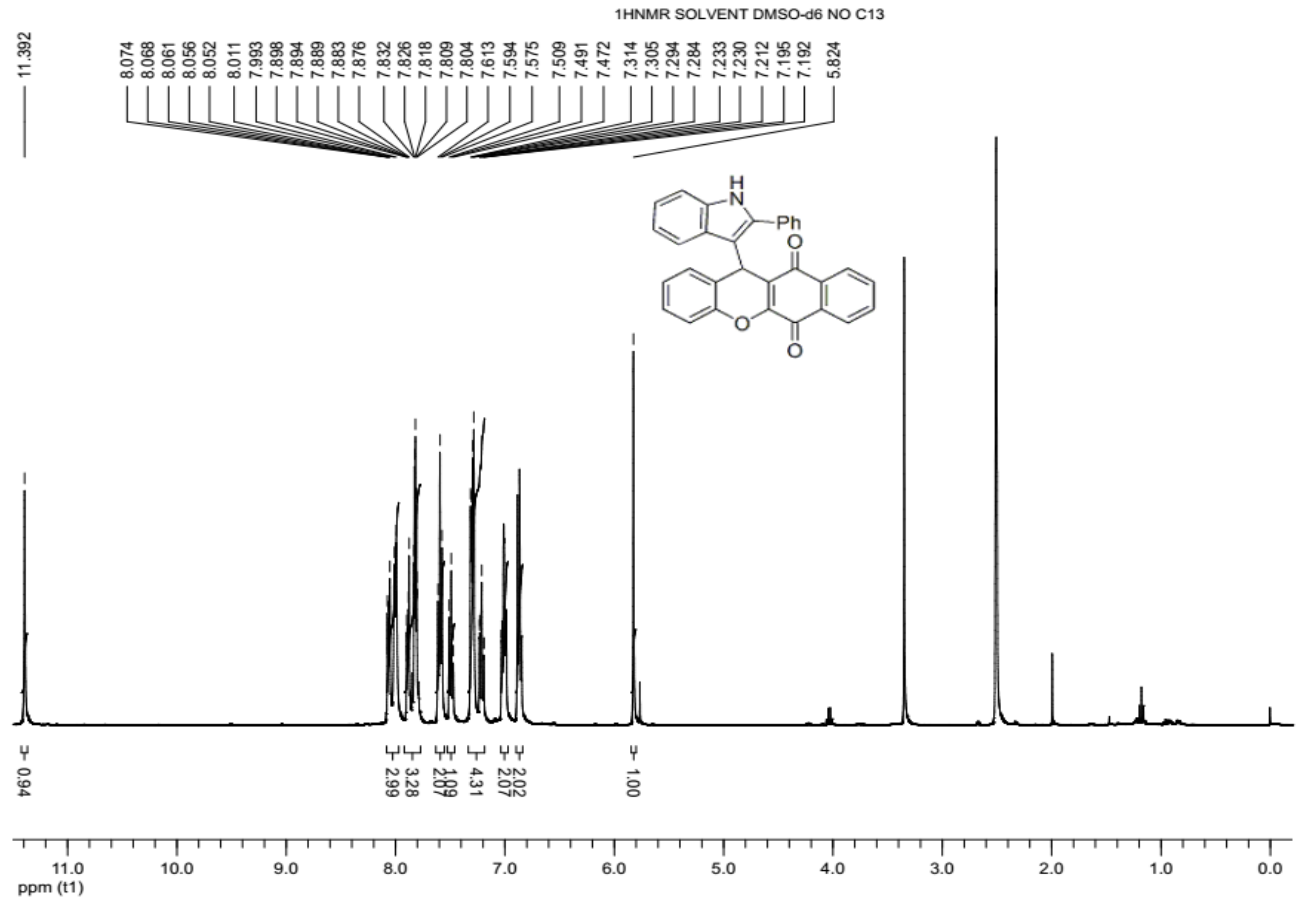

Figure S3 ^{1}H NMR of **4**a

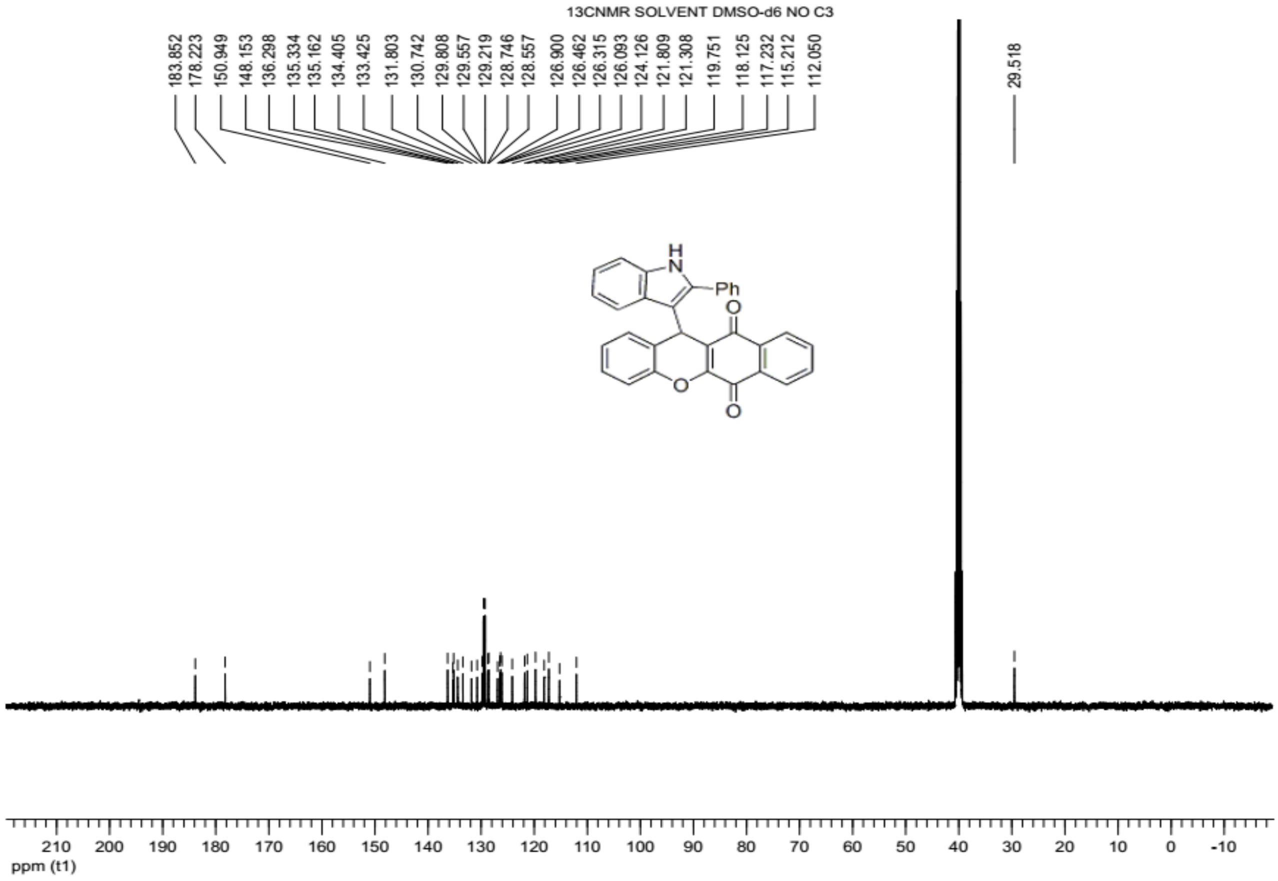

Figure S4 ^{13}C NMR of **4a**

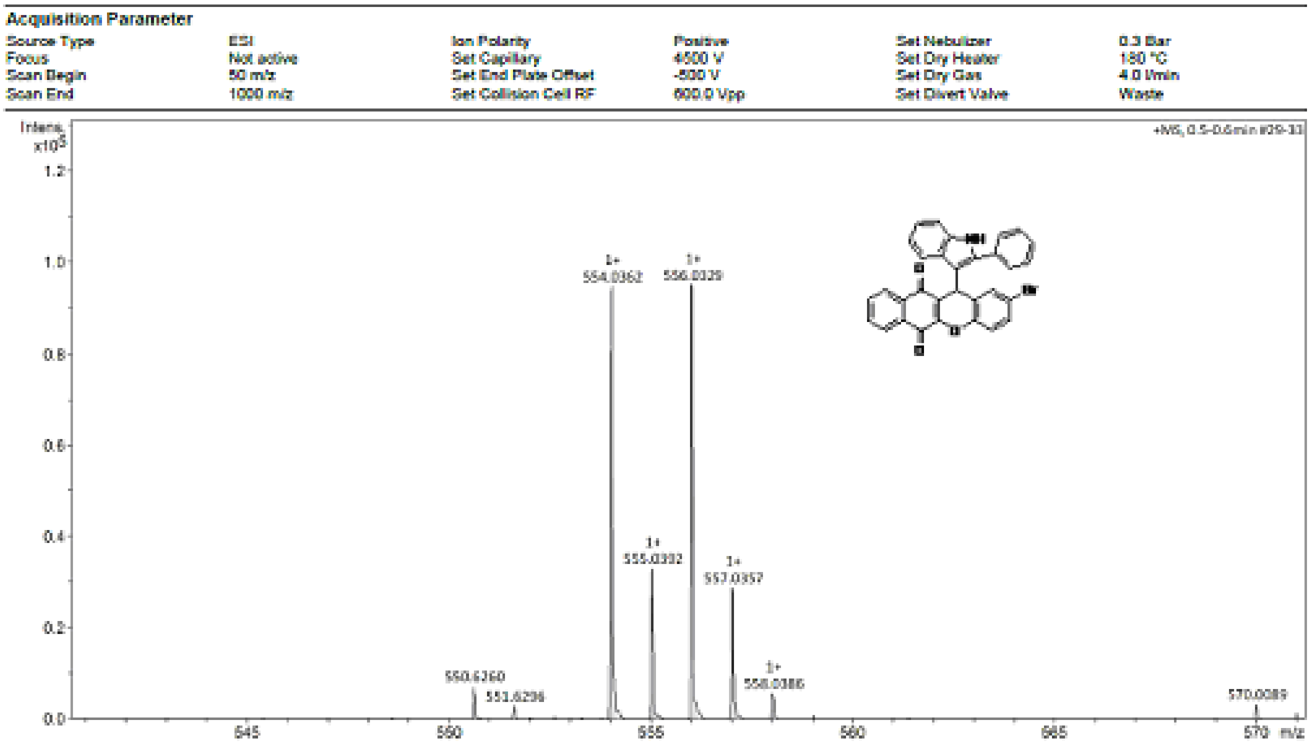

Figure S5 HRMS of **4b**

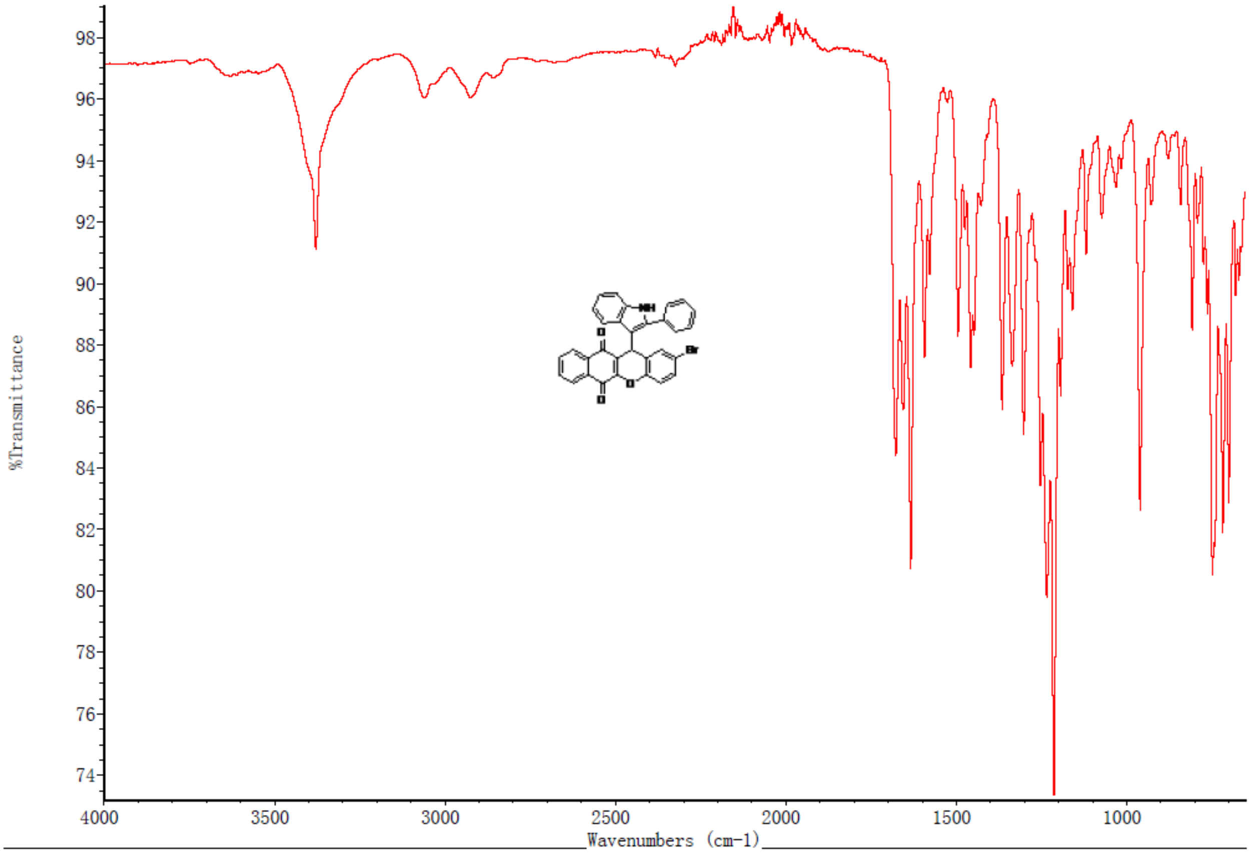

Figure S6 IR of **4b**

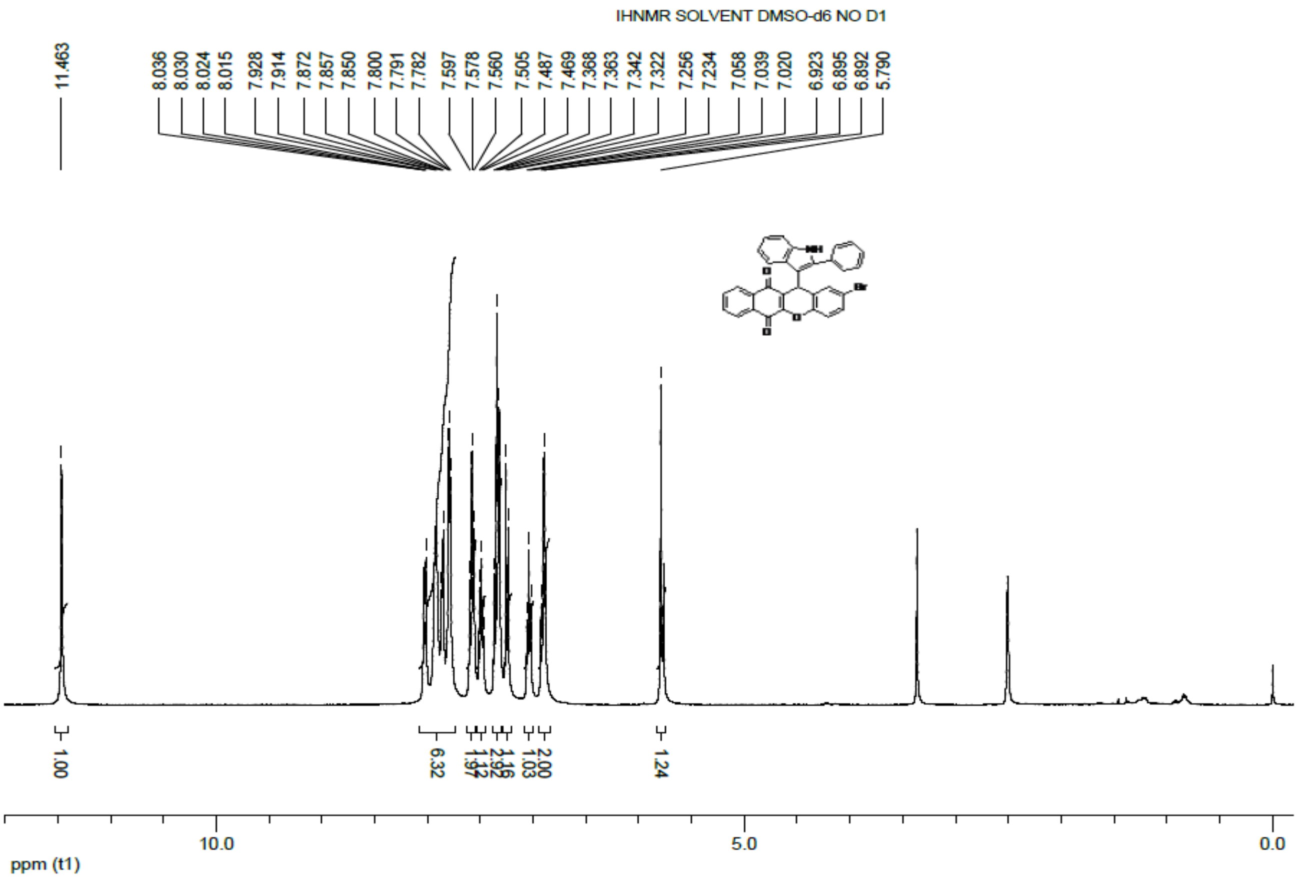

Figure S7 1H NMR of **4b**

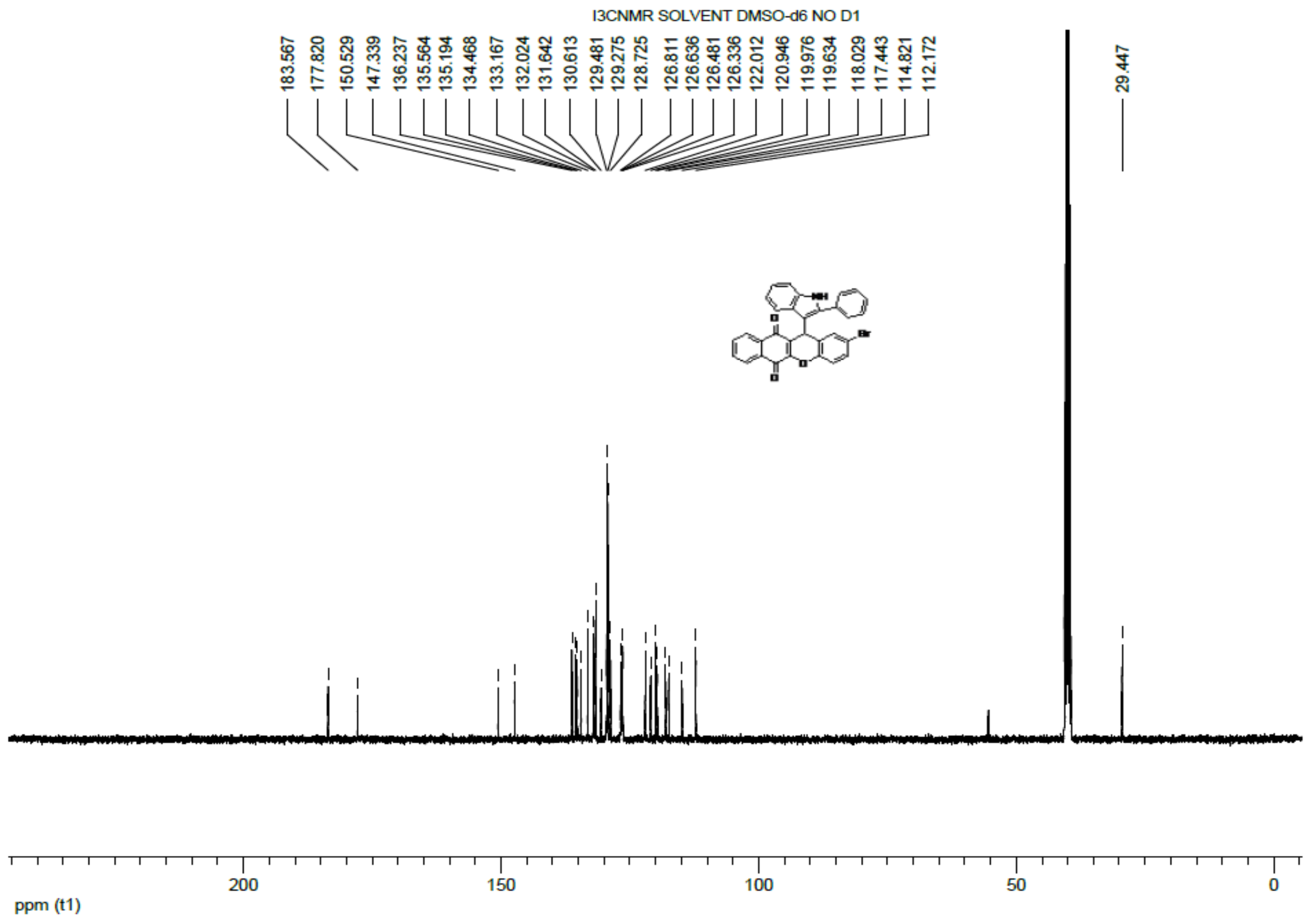

Figure S8 13C NMR of **4b**

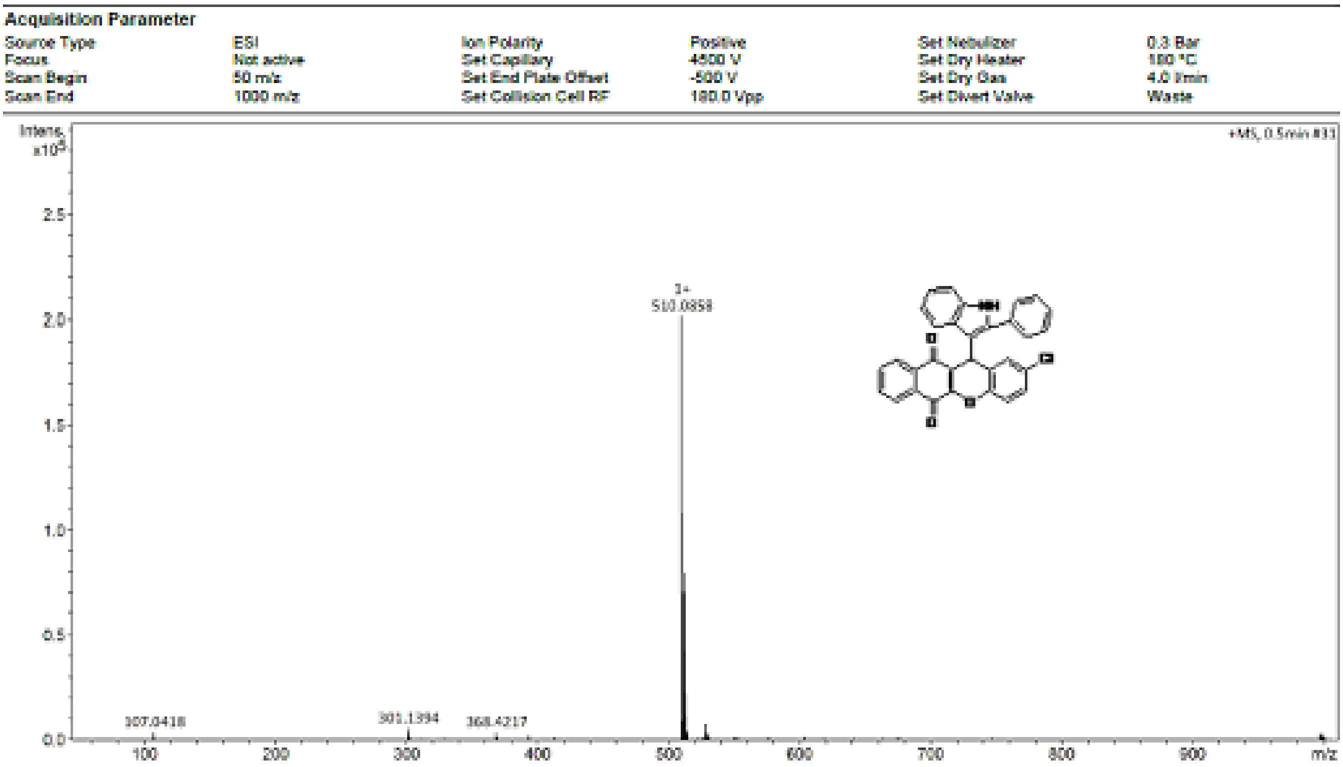

Figure S9 HRMS of **4c**

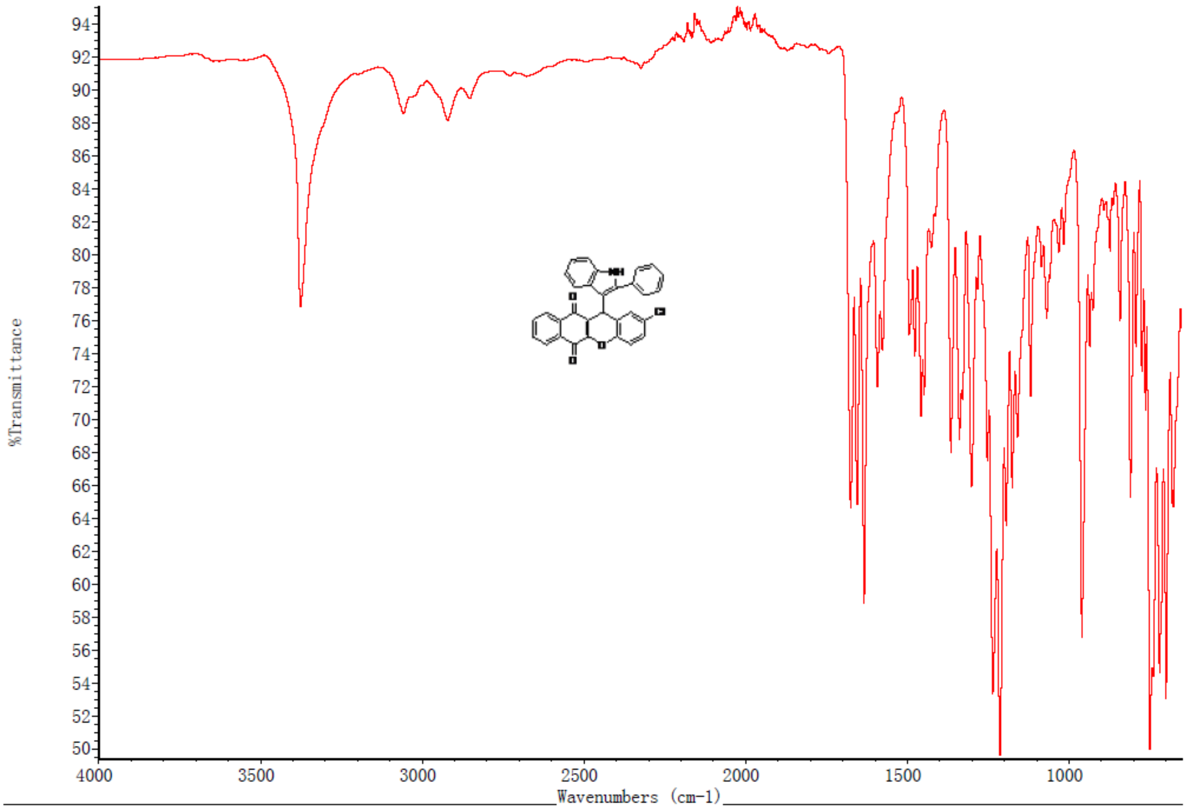

Figure S10 IR of **4c**

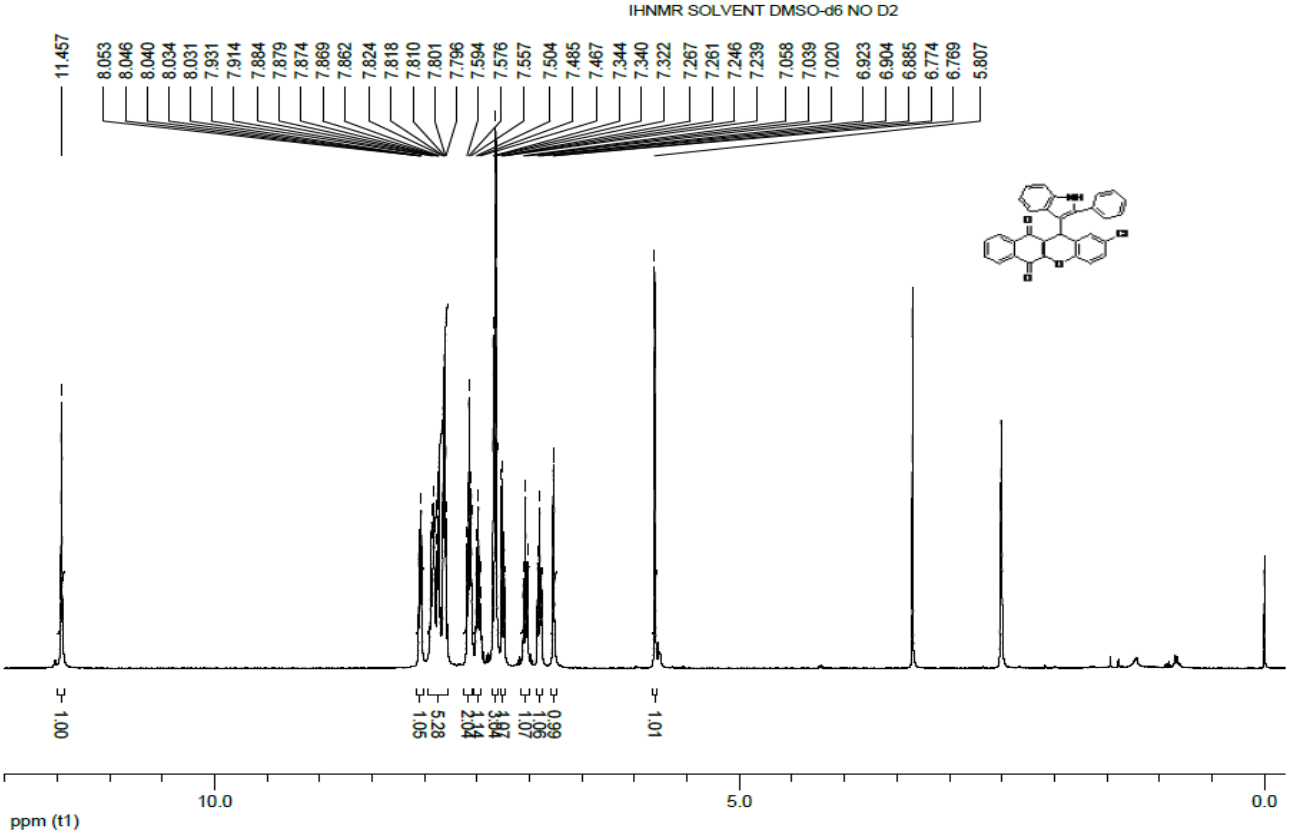

Figure S11 1H NMR of **4c**

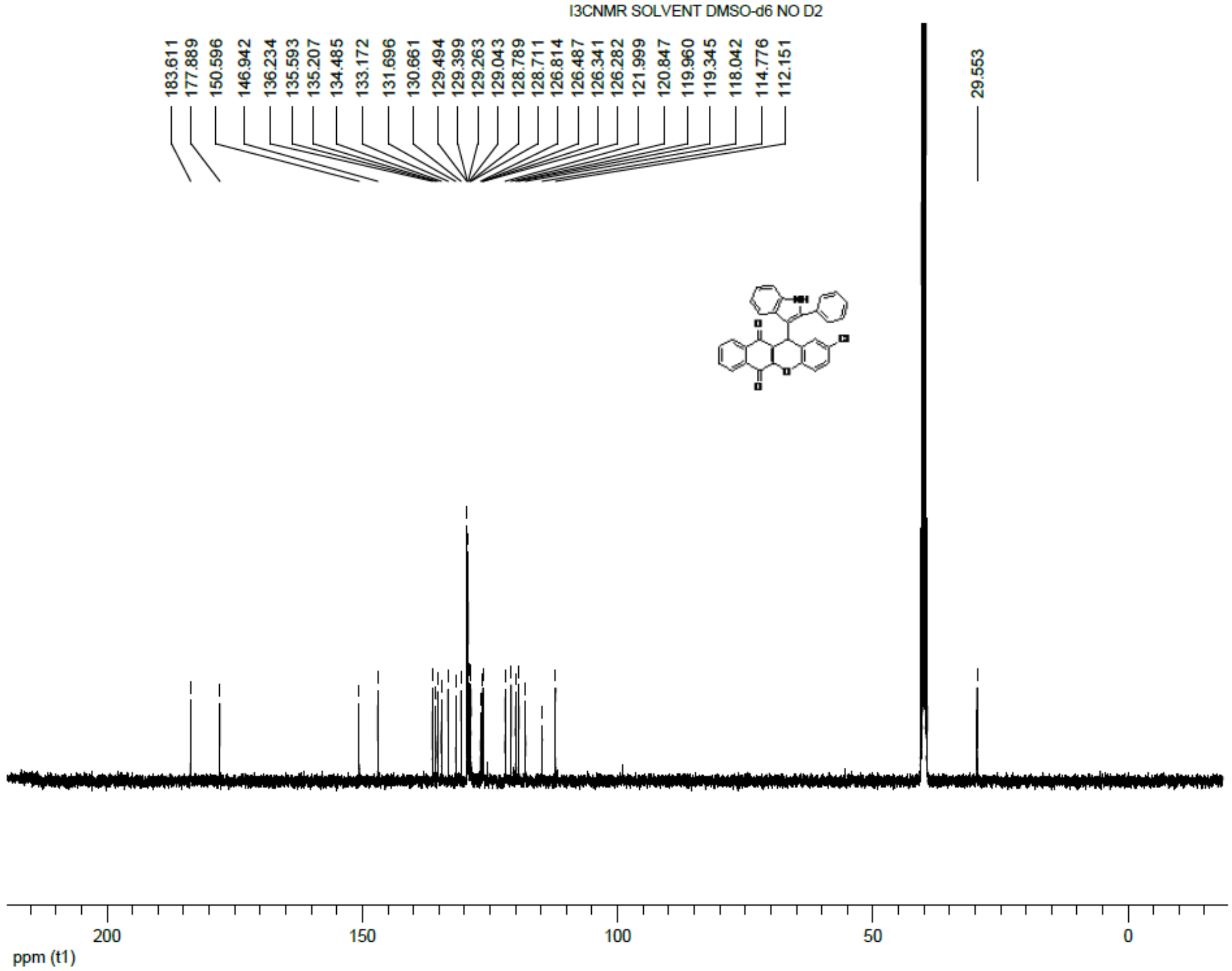

Figure S12 13C NMR of **4c**

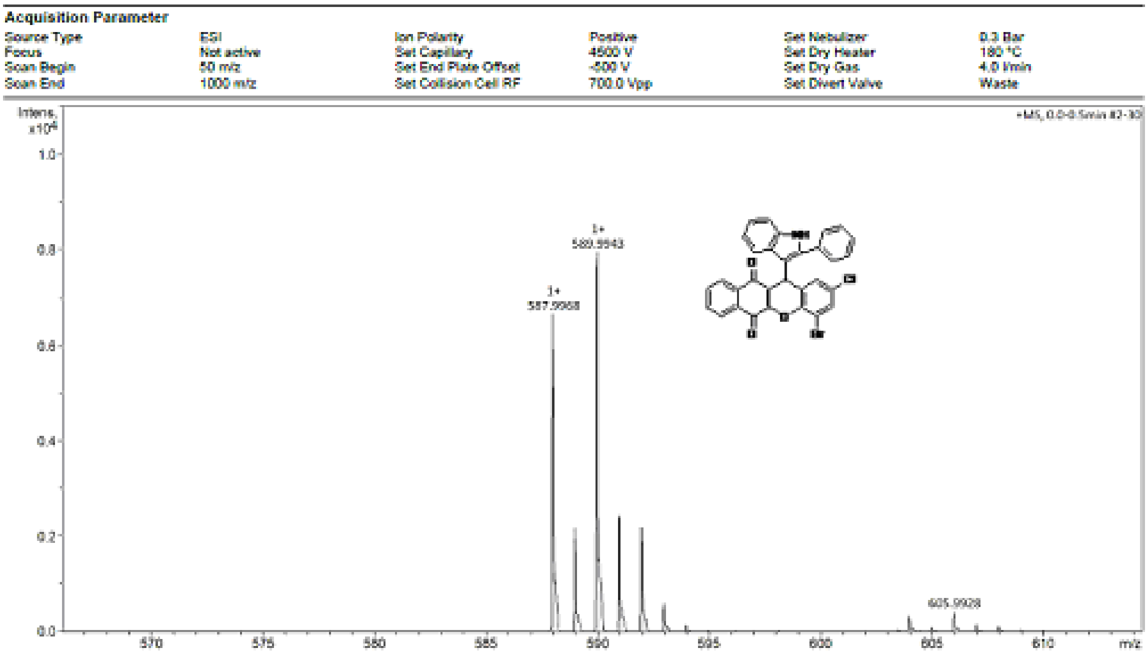

Figure S13 HRMS of **4d**

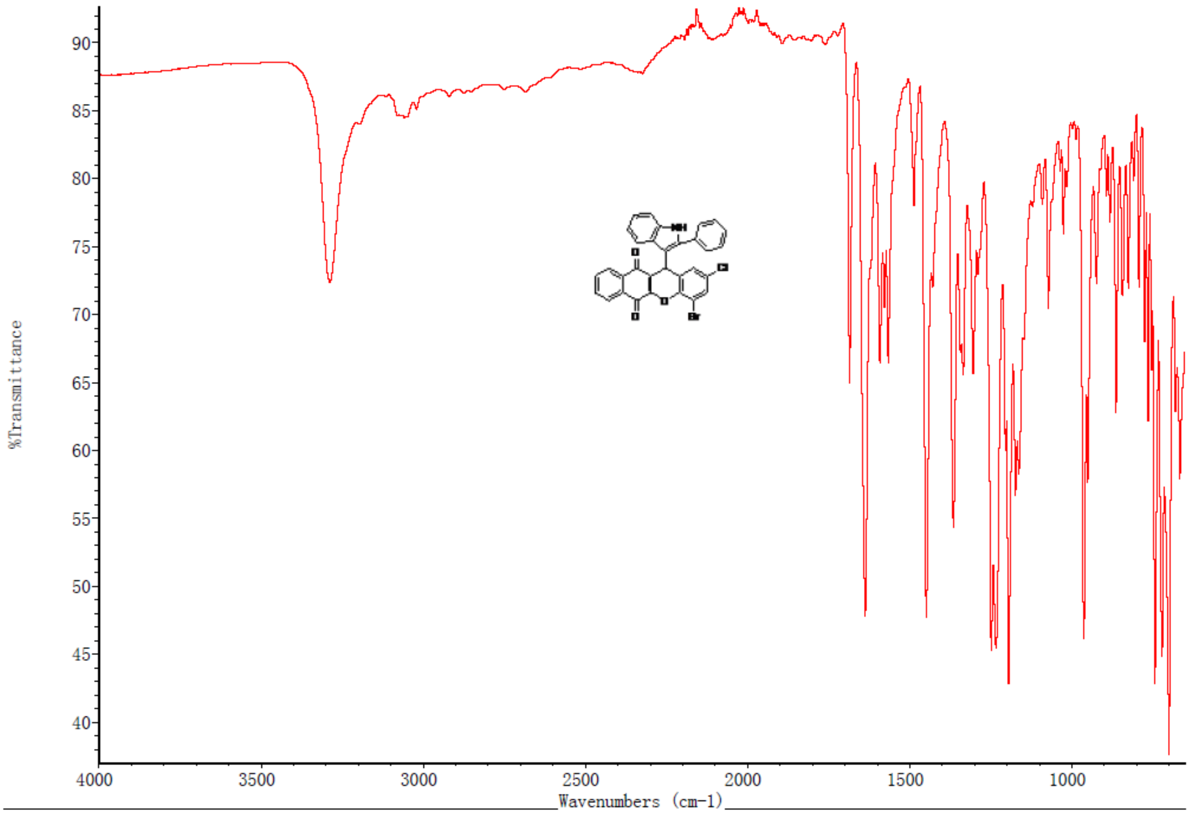

Figure S14 IR of **4d**

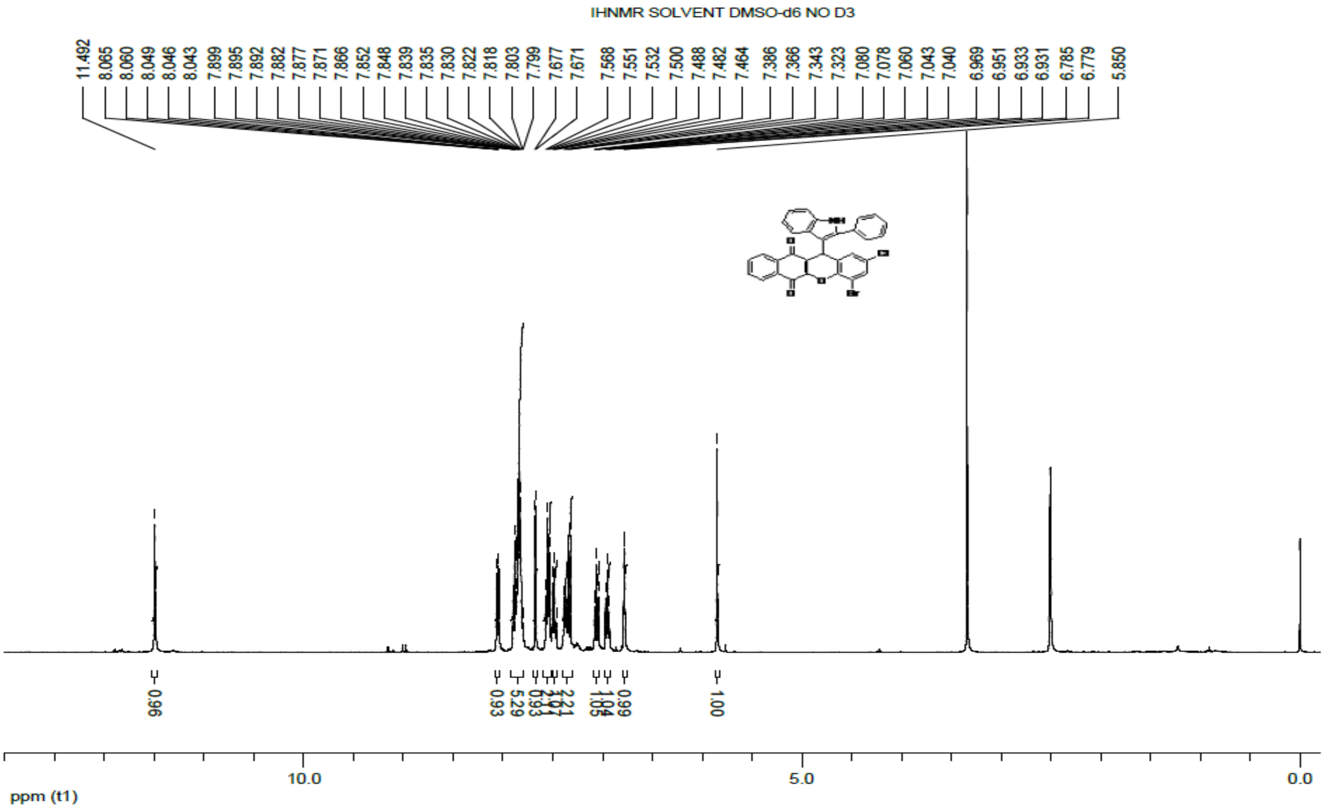

Figure S15 1H NMR of **4d**

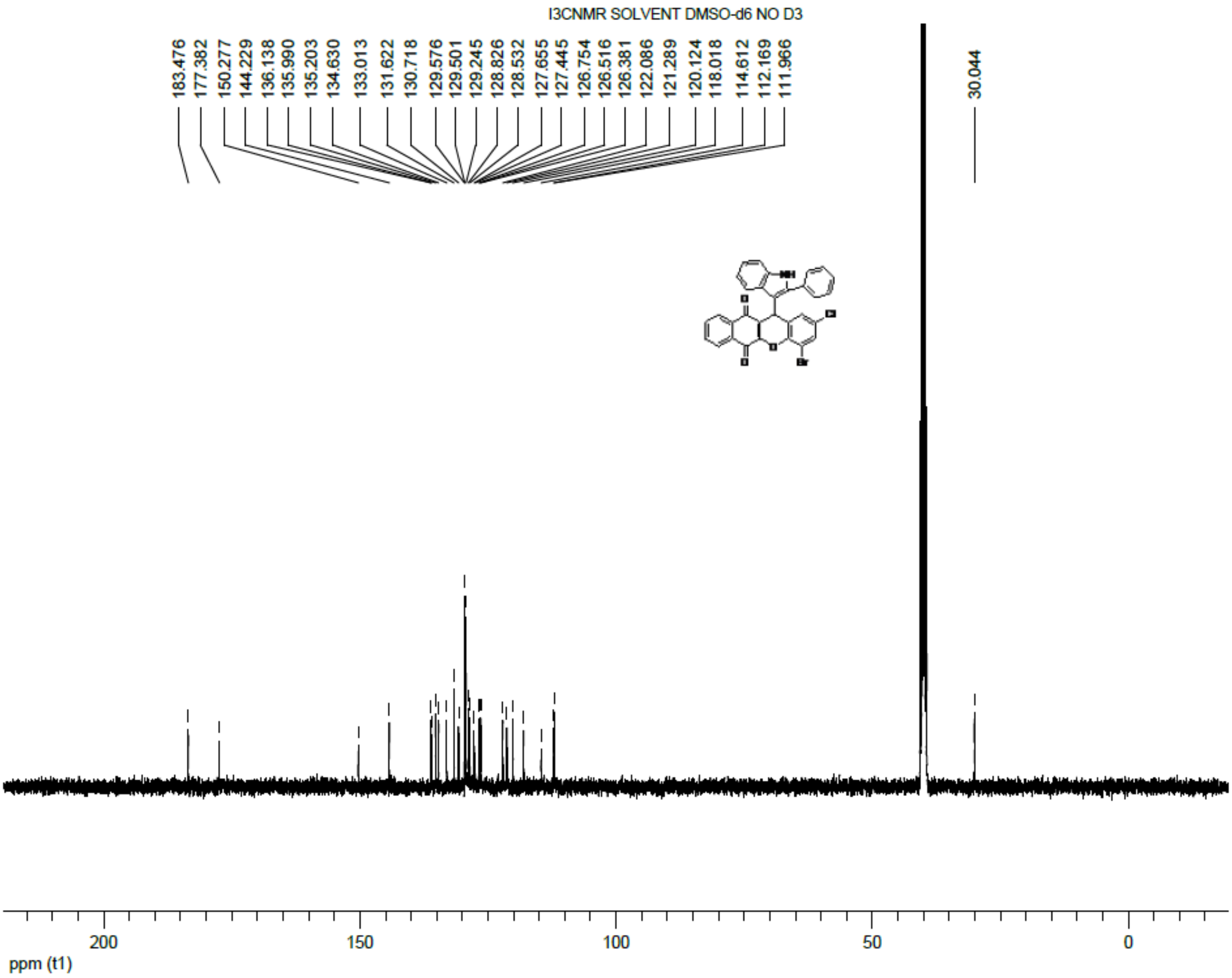

Figure S16 13C NMR of **4d**

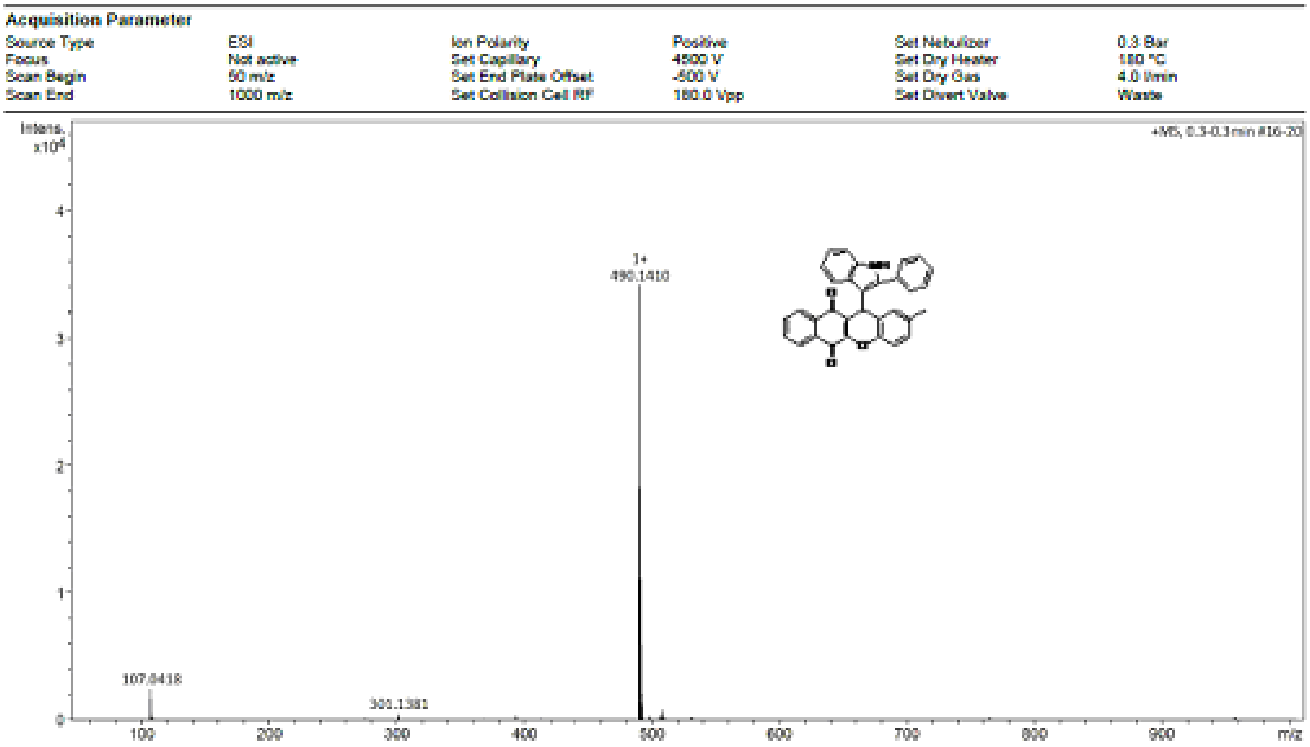

Figure S17 HRMS of **4e**

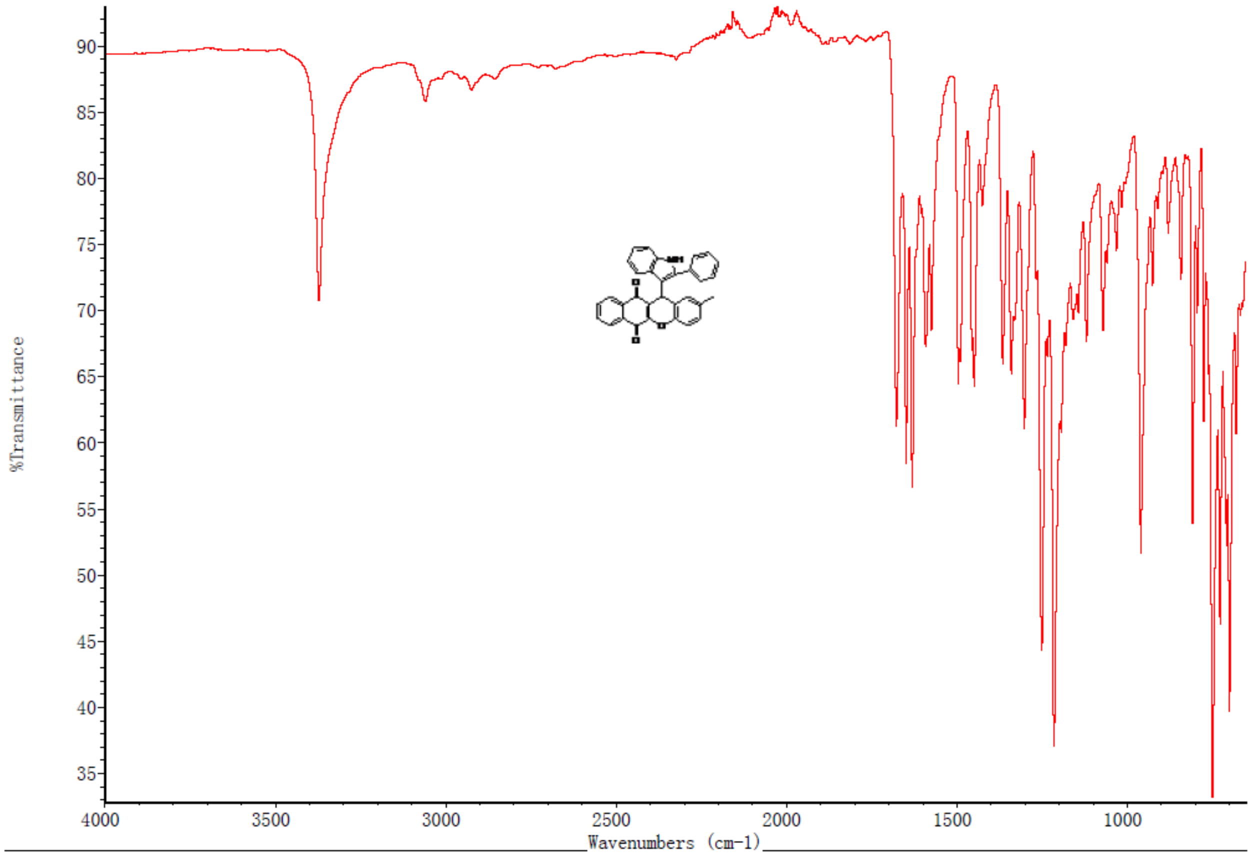

Figure S18 IR of **4e**

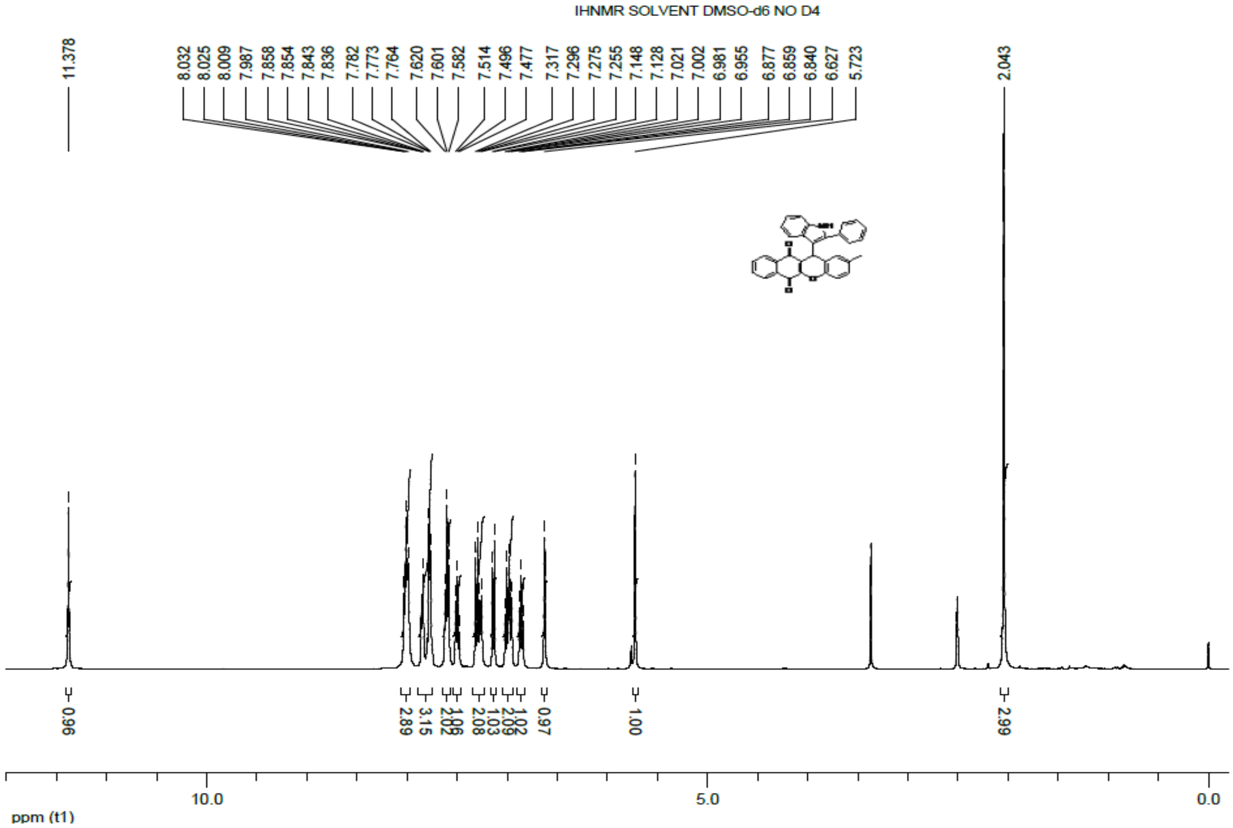

Figure S19 1H NMR of **4e**

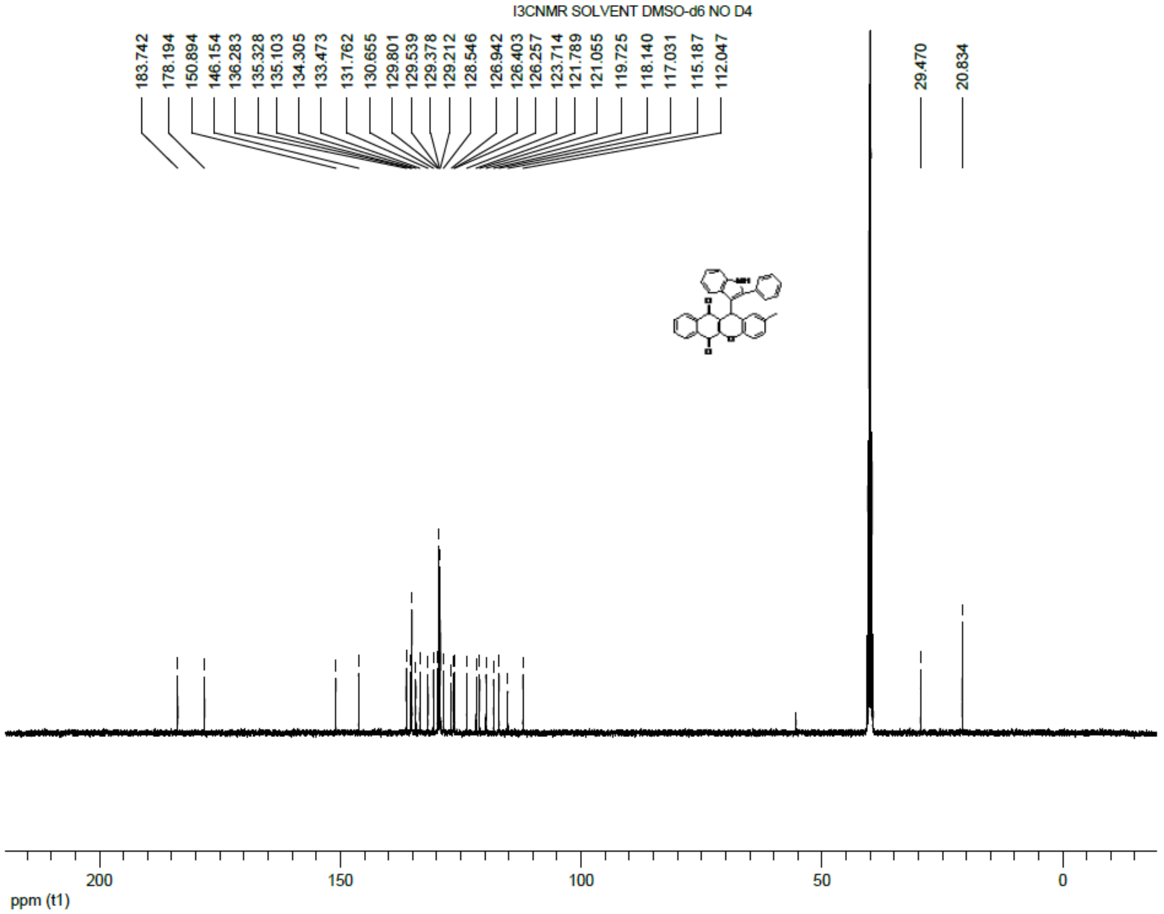

Figure S20 13C NMR of **4e**

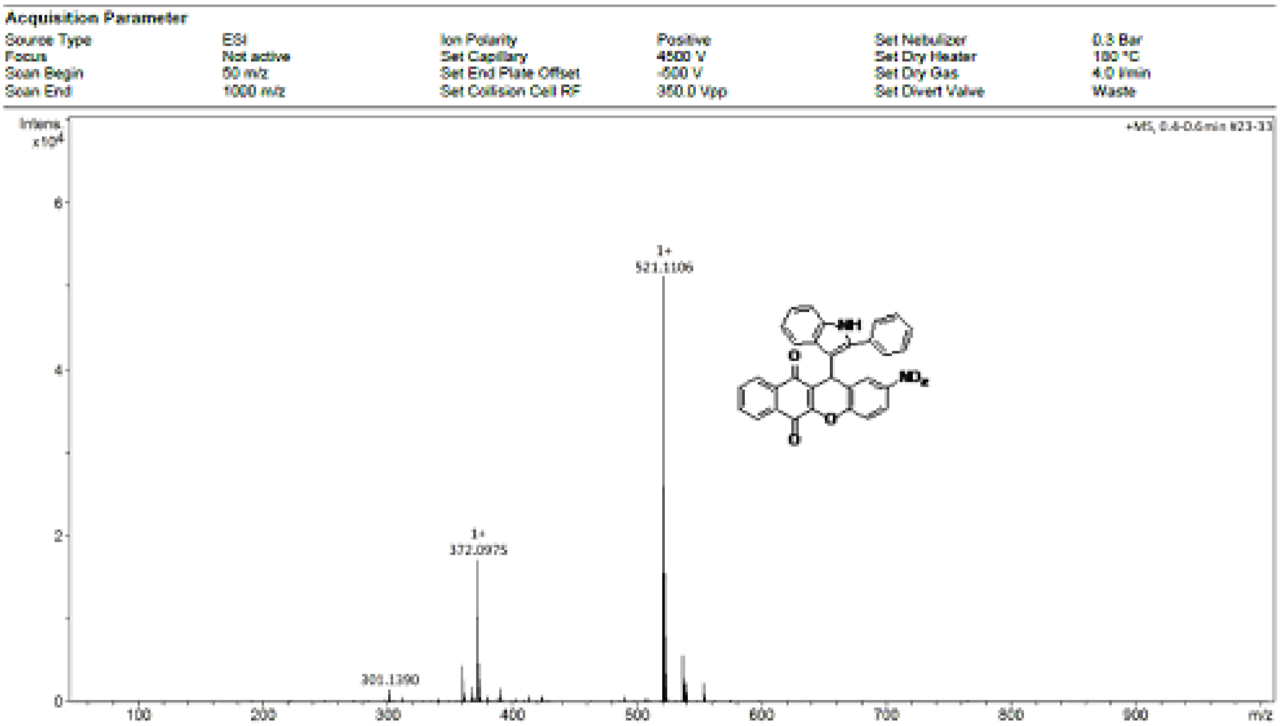

Figure S21 HRMS of **4f**

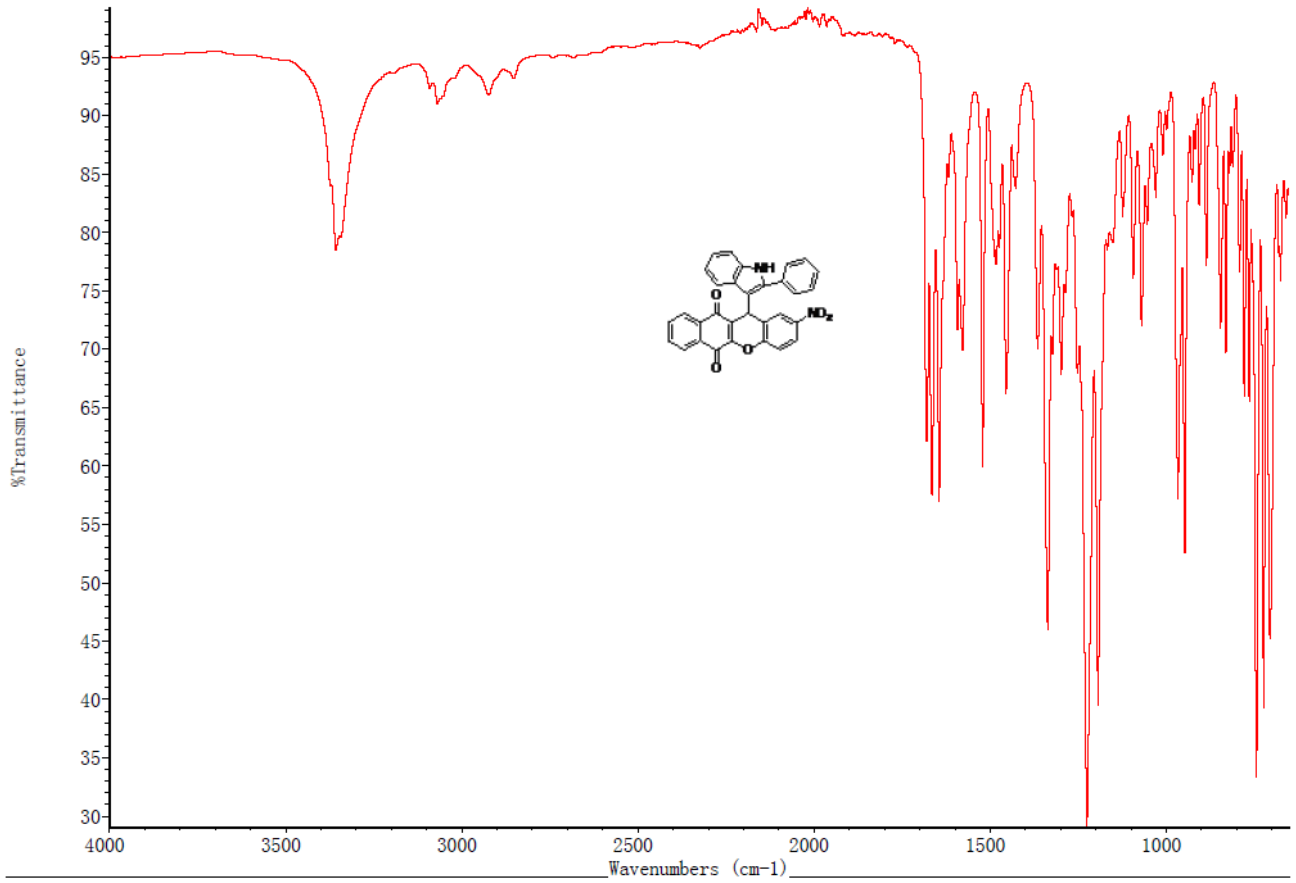

Figure S22 IR of **4f**

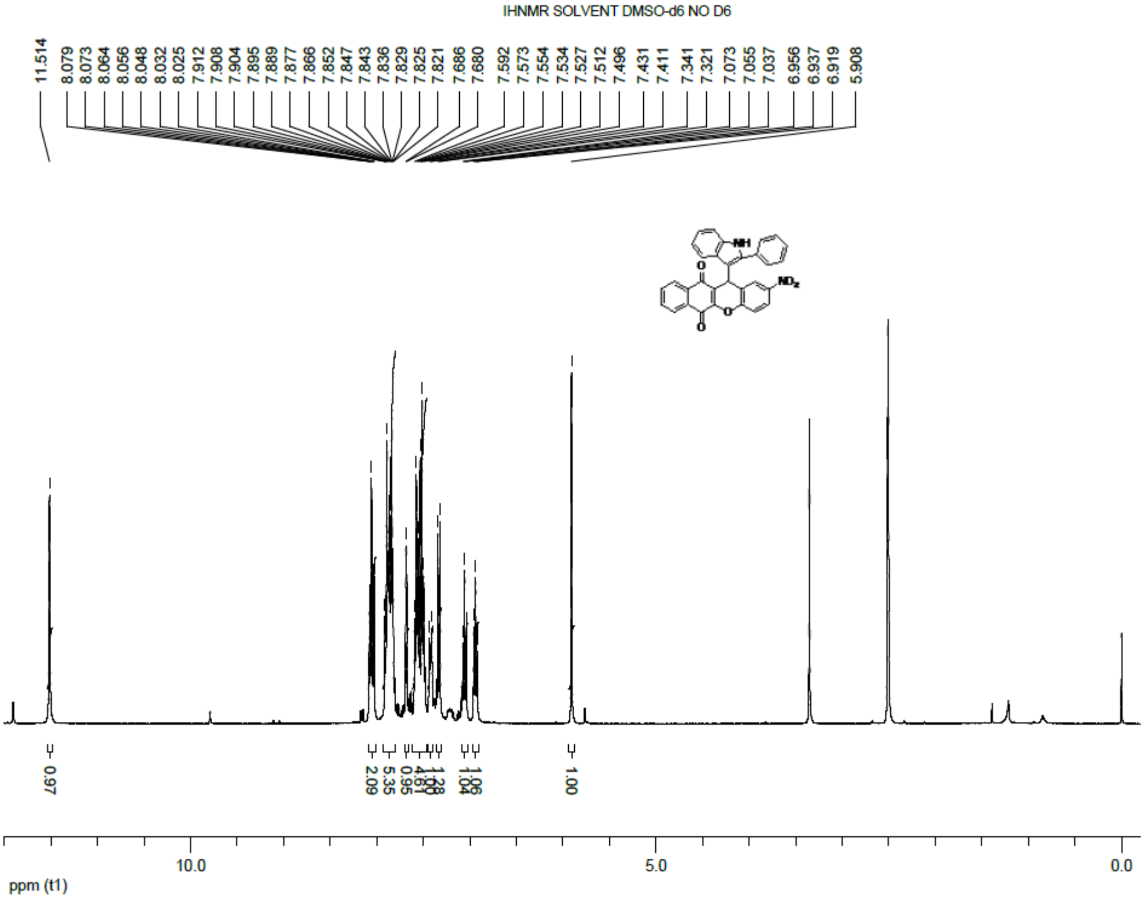

Figure S23 1H NMR of **4f**

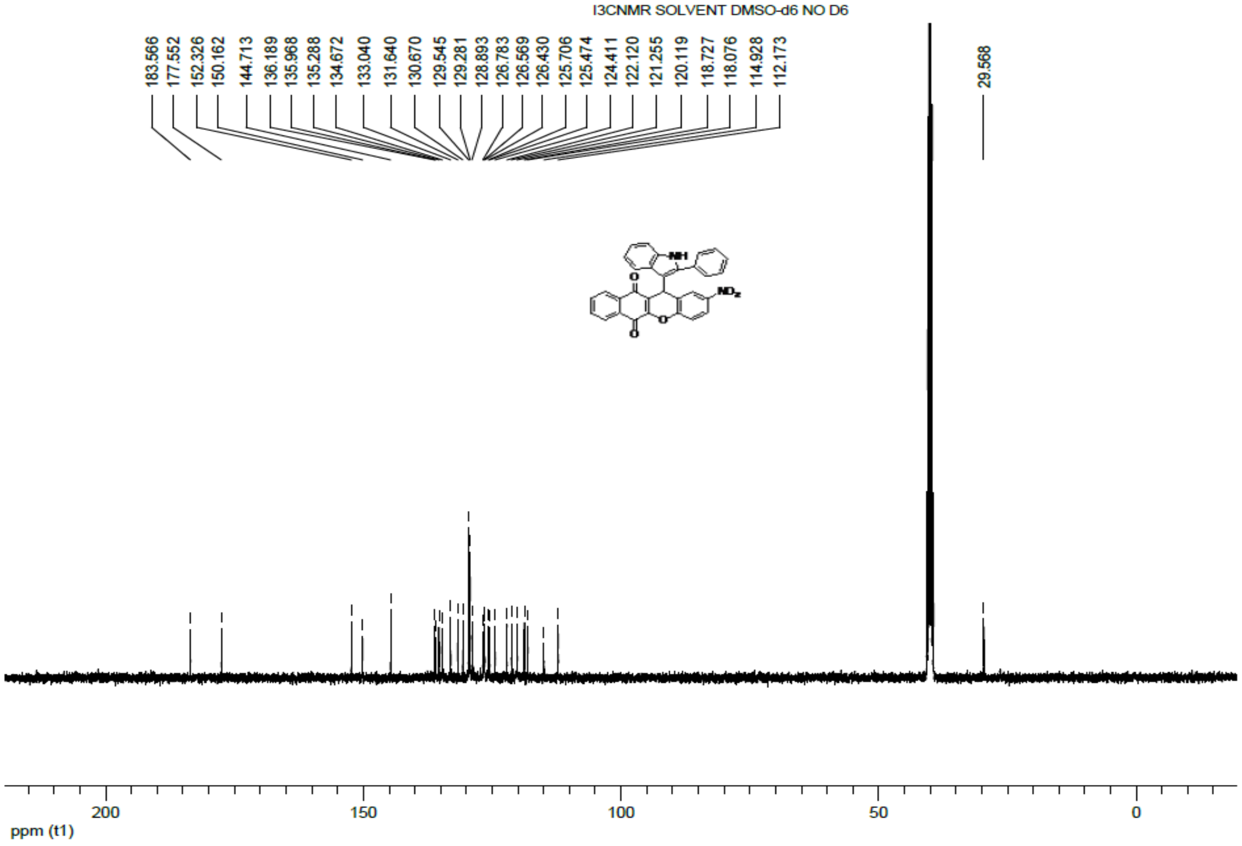

Figure S24 13C NMR of **4f**

Acquisition Parameter

Source Type	ESI	Ion Polarity	Positive	Set Nebulizer	0.3 Bar
Focus	Not active	Set Capillary	4500 V	Set Dry Heater	180 °C
Scan Begin	50 m/z	Set End Plate Offset	-500 V	Set Dry Gas	4.0 l/min
Scan End	1000 m/z	Set Collision Cell RF	280.0 Vpp	Set Divert Valve	Waste

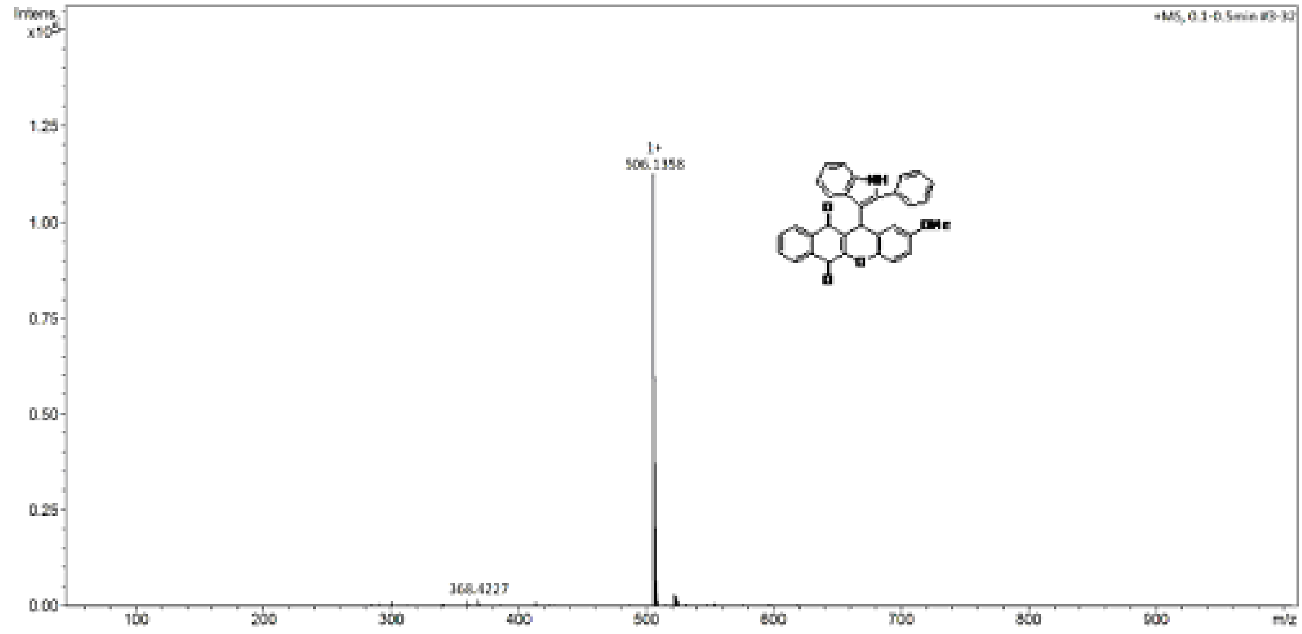

Figure S25 HRMS of **4g**

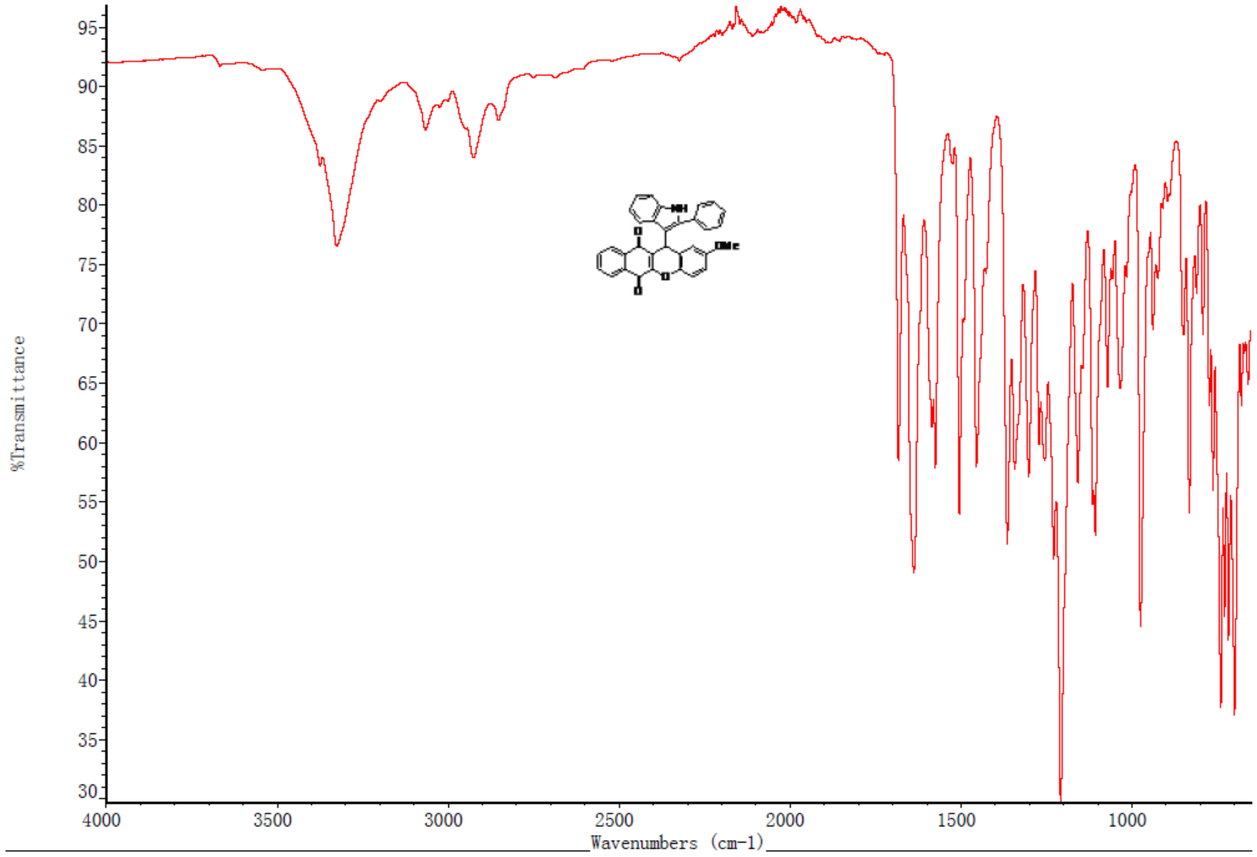

Figure S26 IR of **4g**

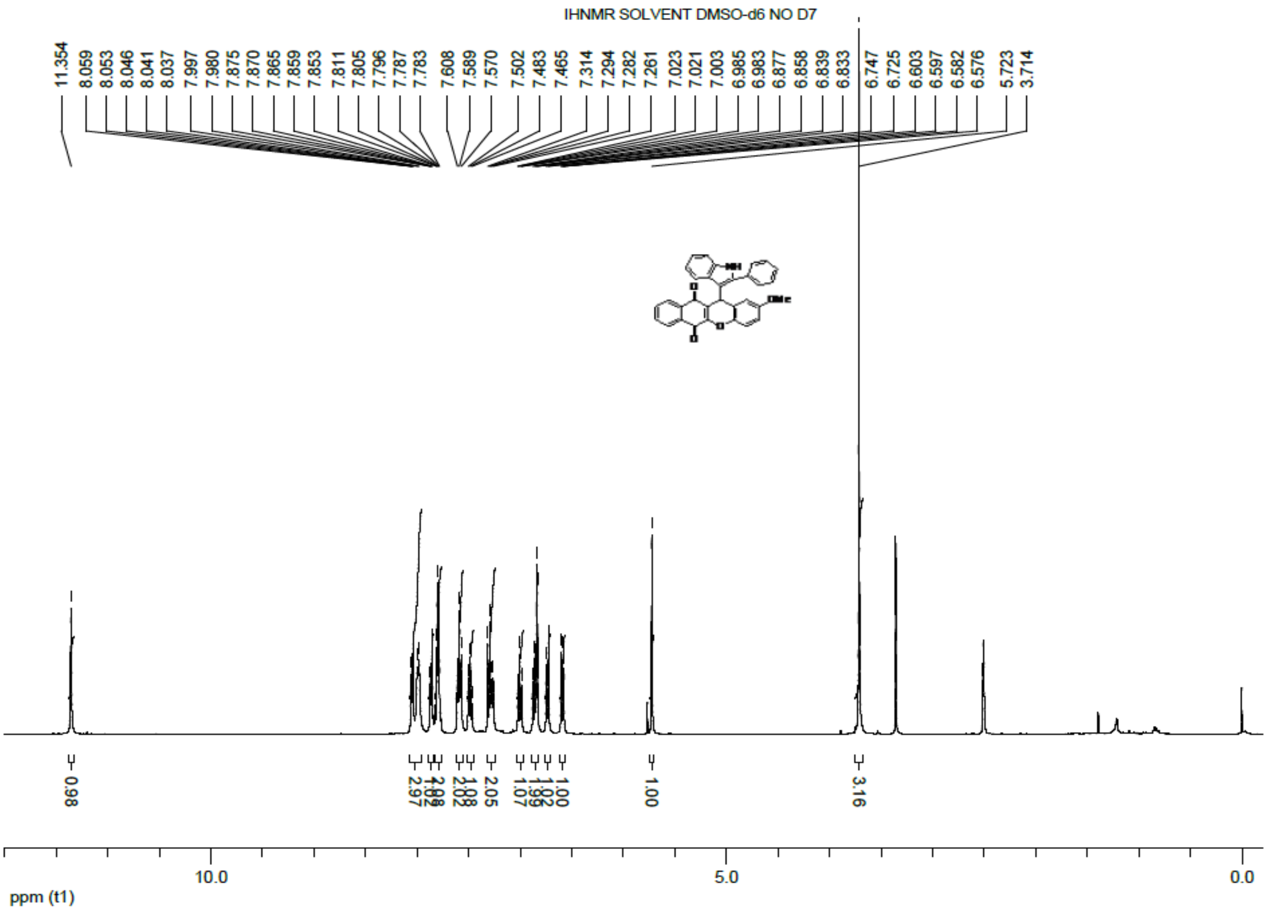

Figure S27 1H NMR of **4g**

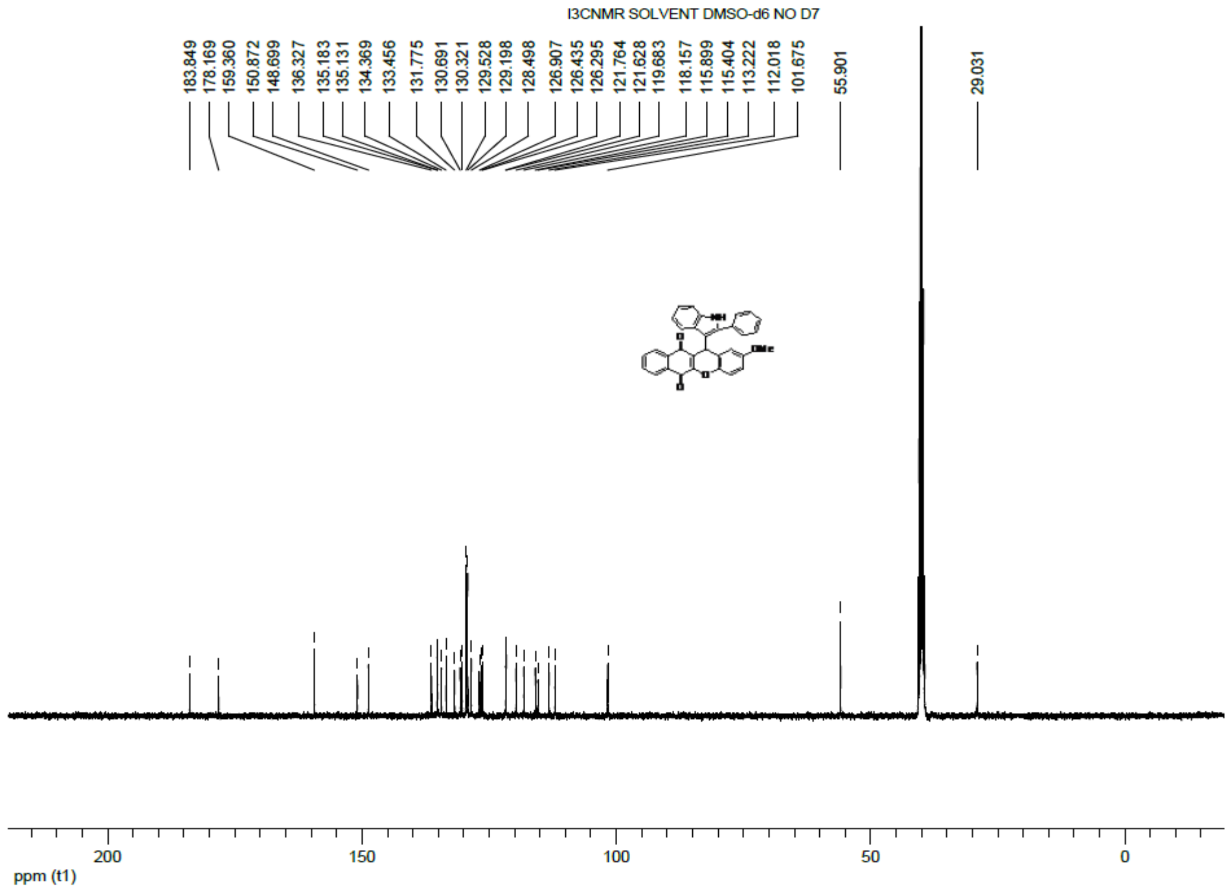

Figure S28 13C NMR of **4g**

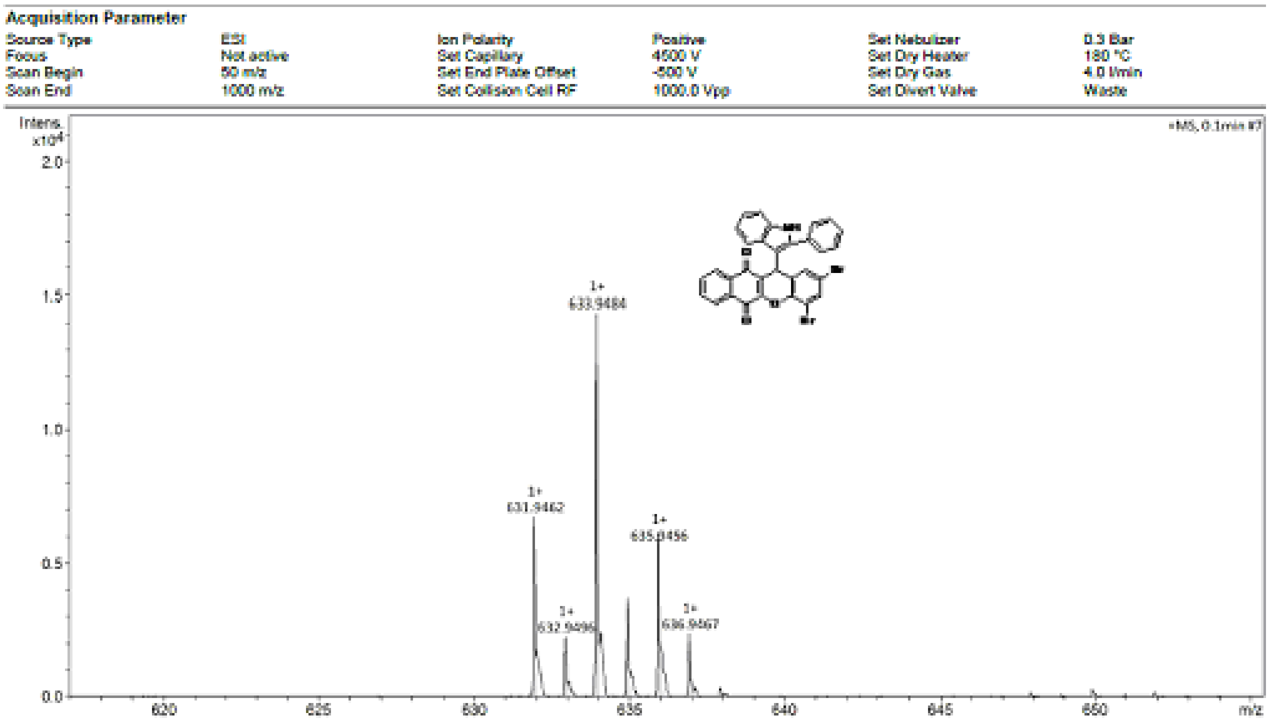

Figure S29 HRMS of **4h**

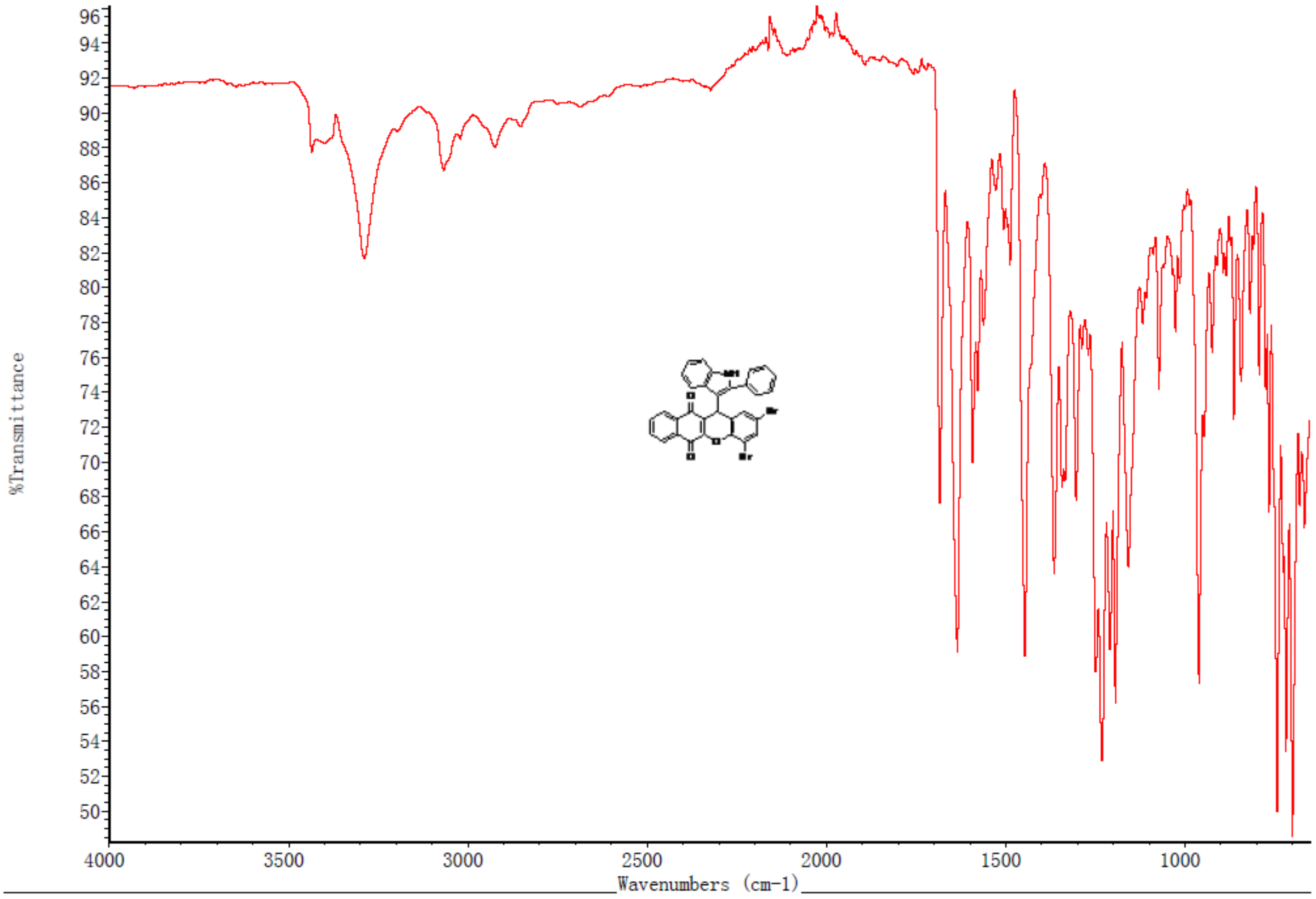

Figure S30 IR of **4h**

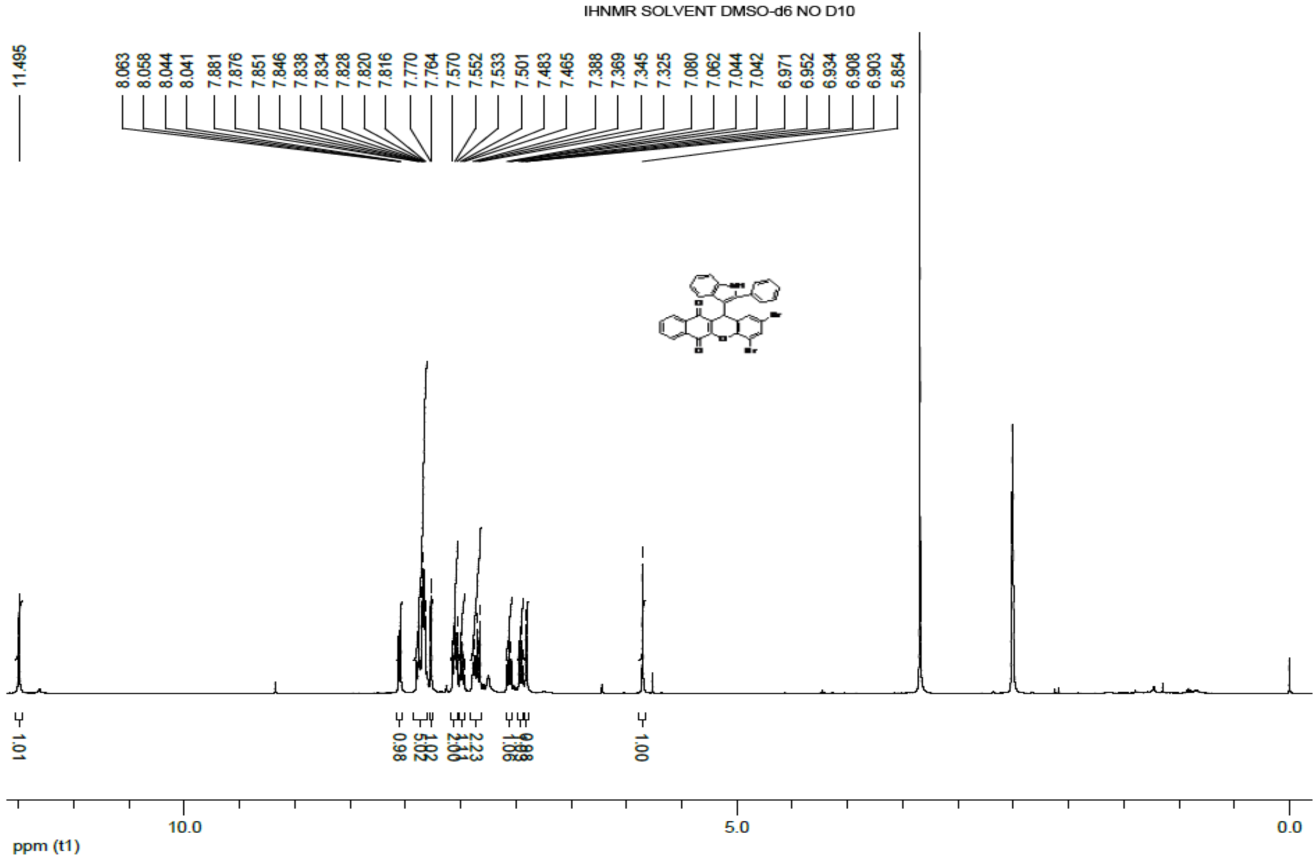

Figure S31 1H NMR of **4h**

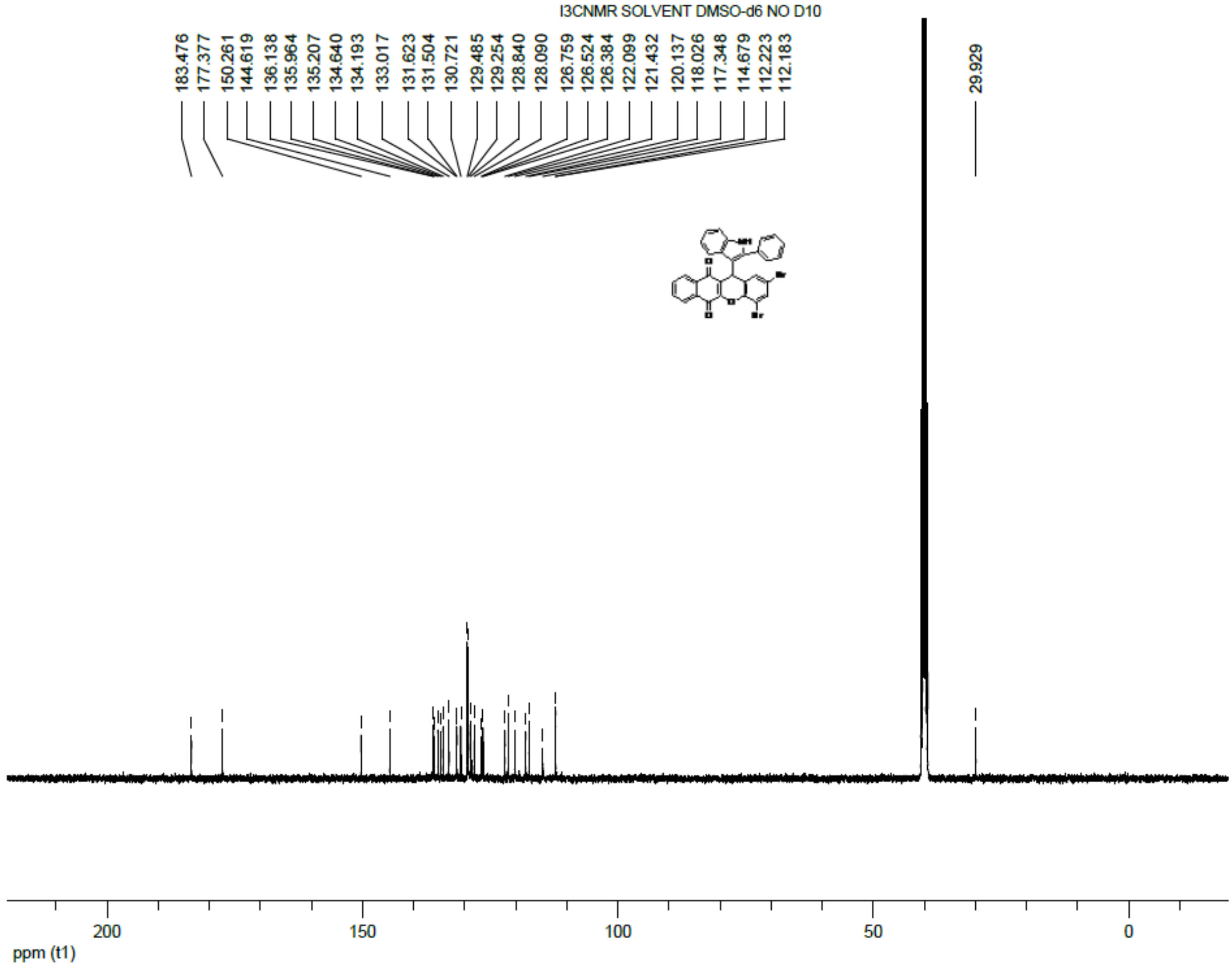

Figure S3213C NMR of **4h**

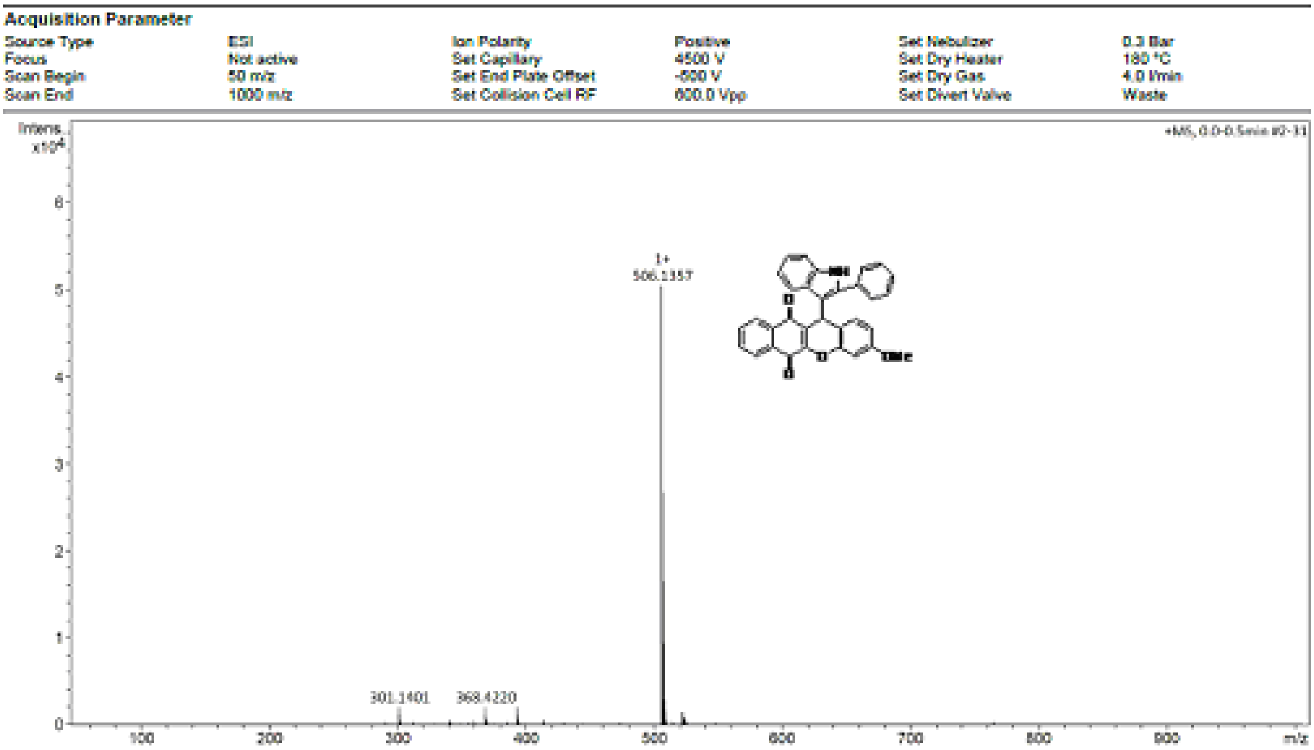

Figure S33 HRMS of **4i**

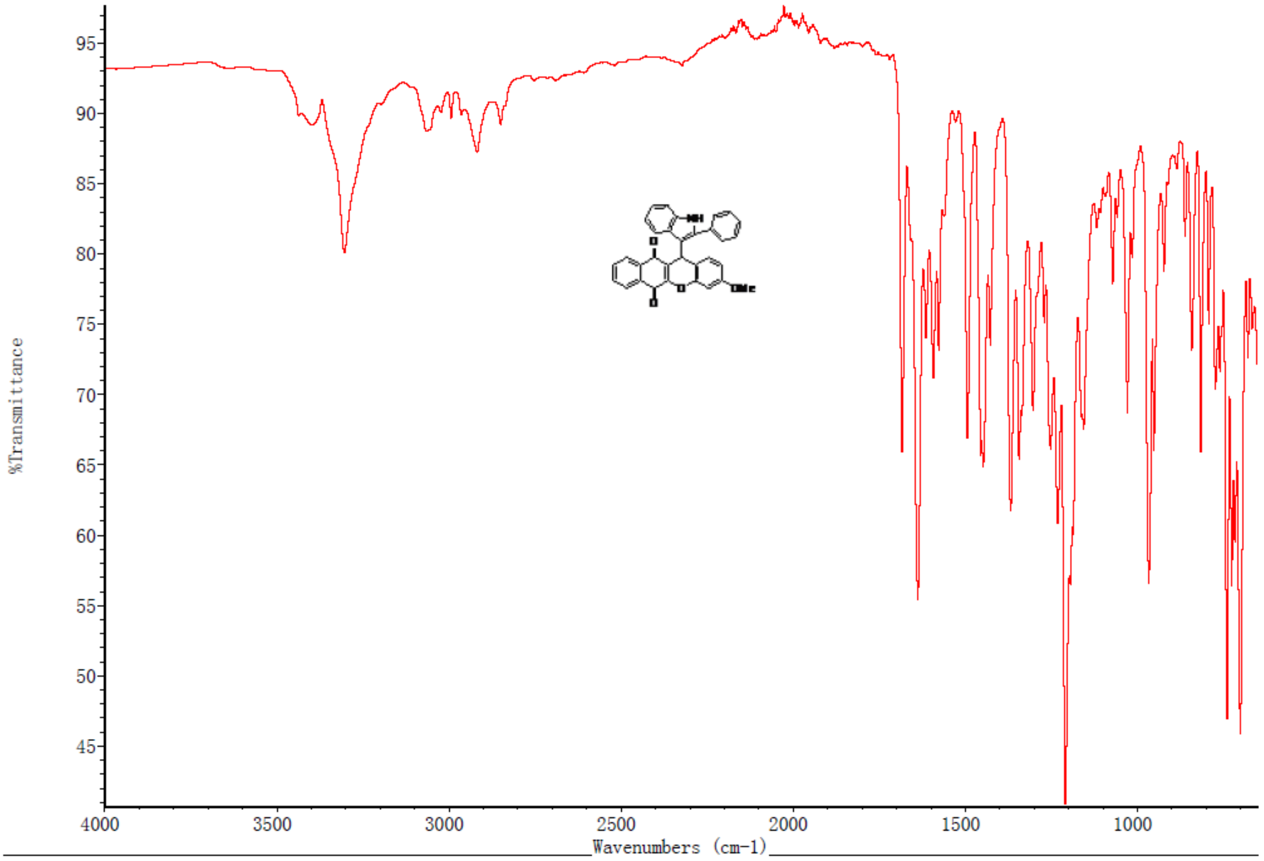

Figure S34 IR of **4i**

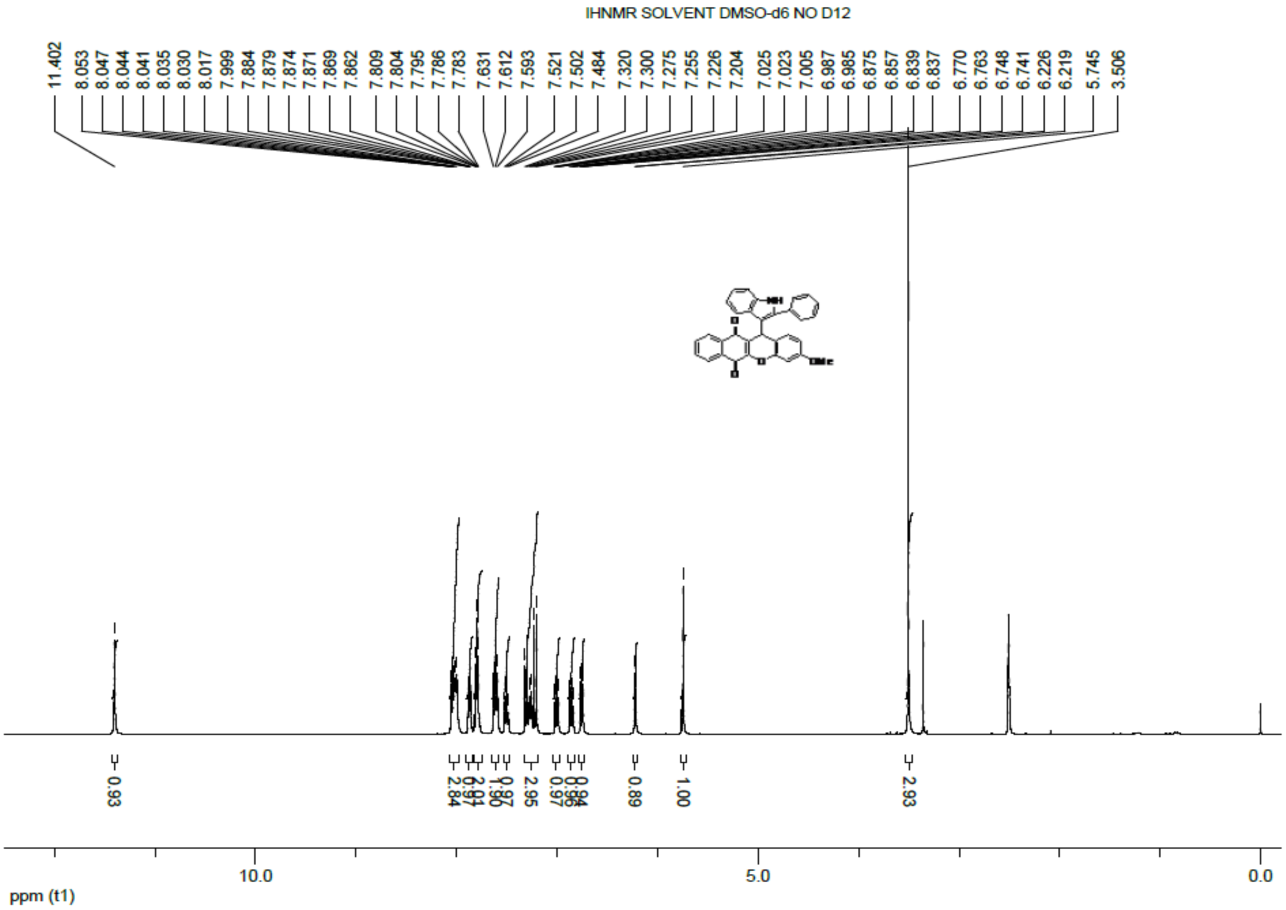

Figure S35 1H NMR of **4i**

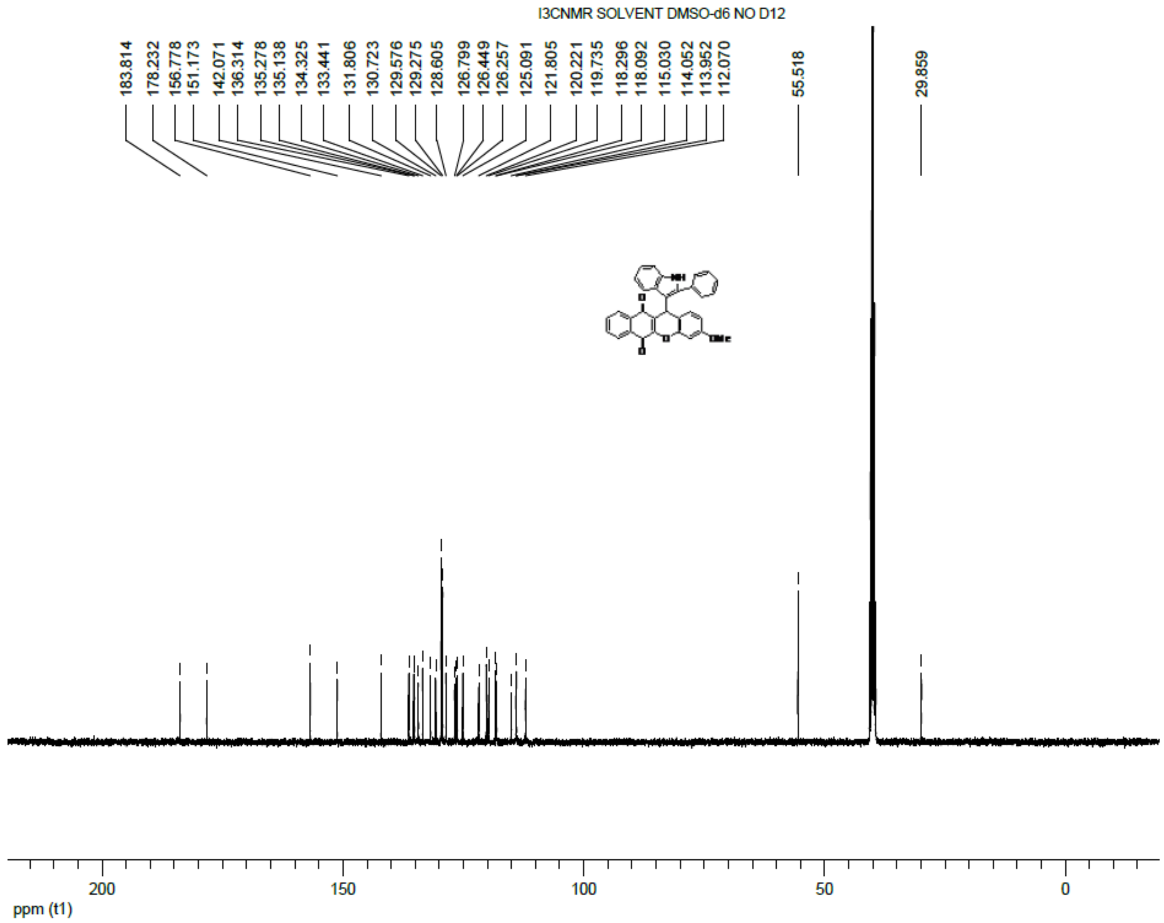

Figure S3613C NMR of **4i**

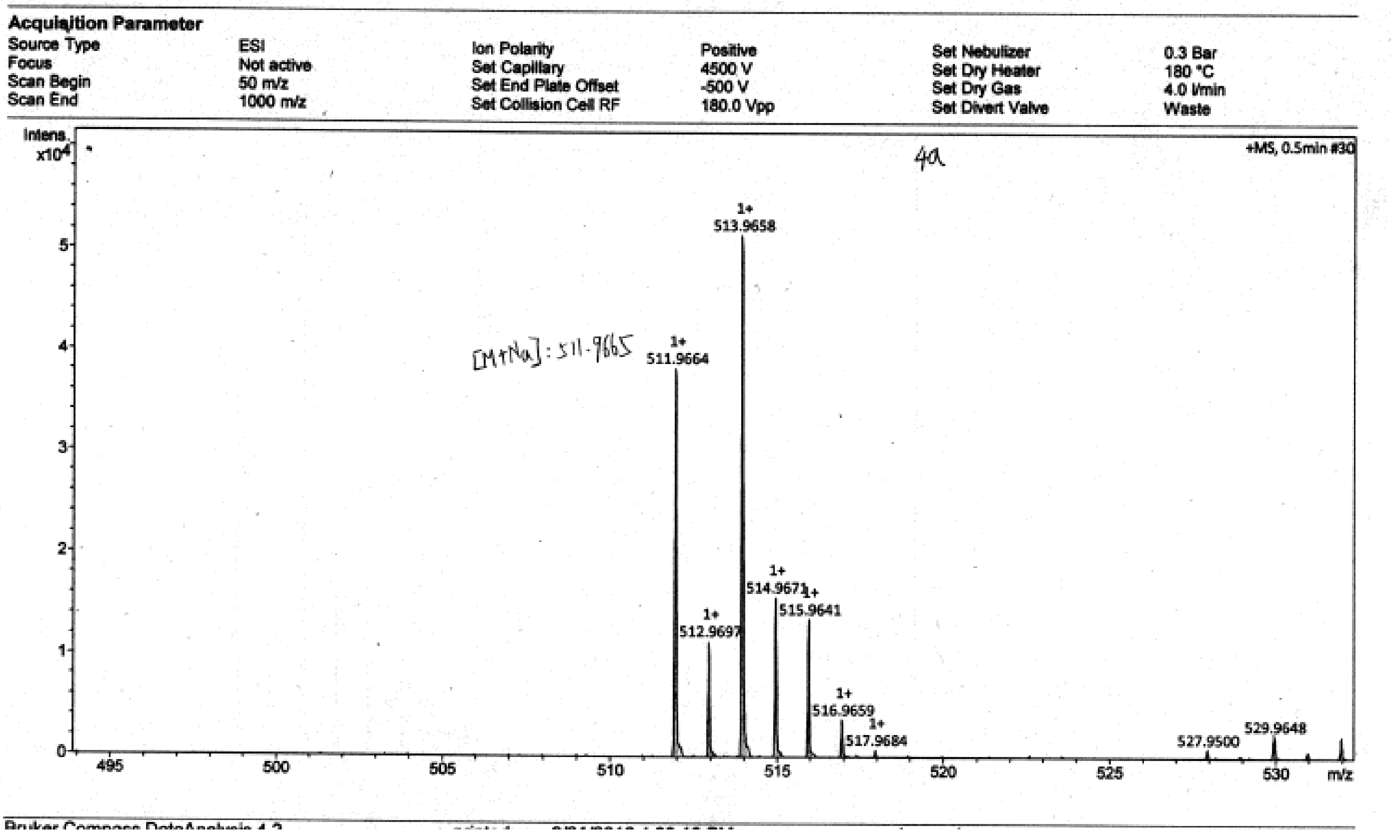

Figure S37 HRMS of **4j**

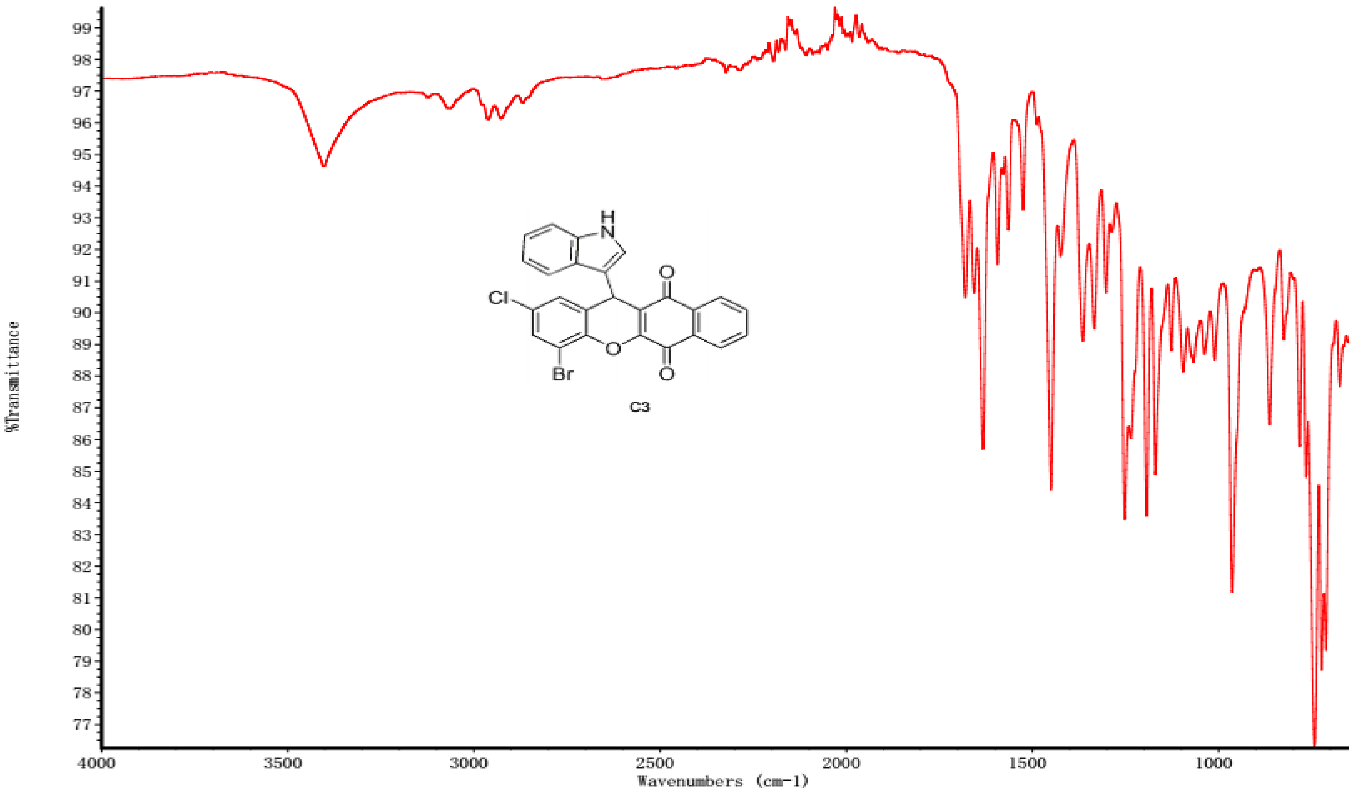

Figure S38 IR of **4j**

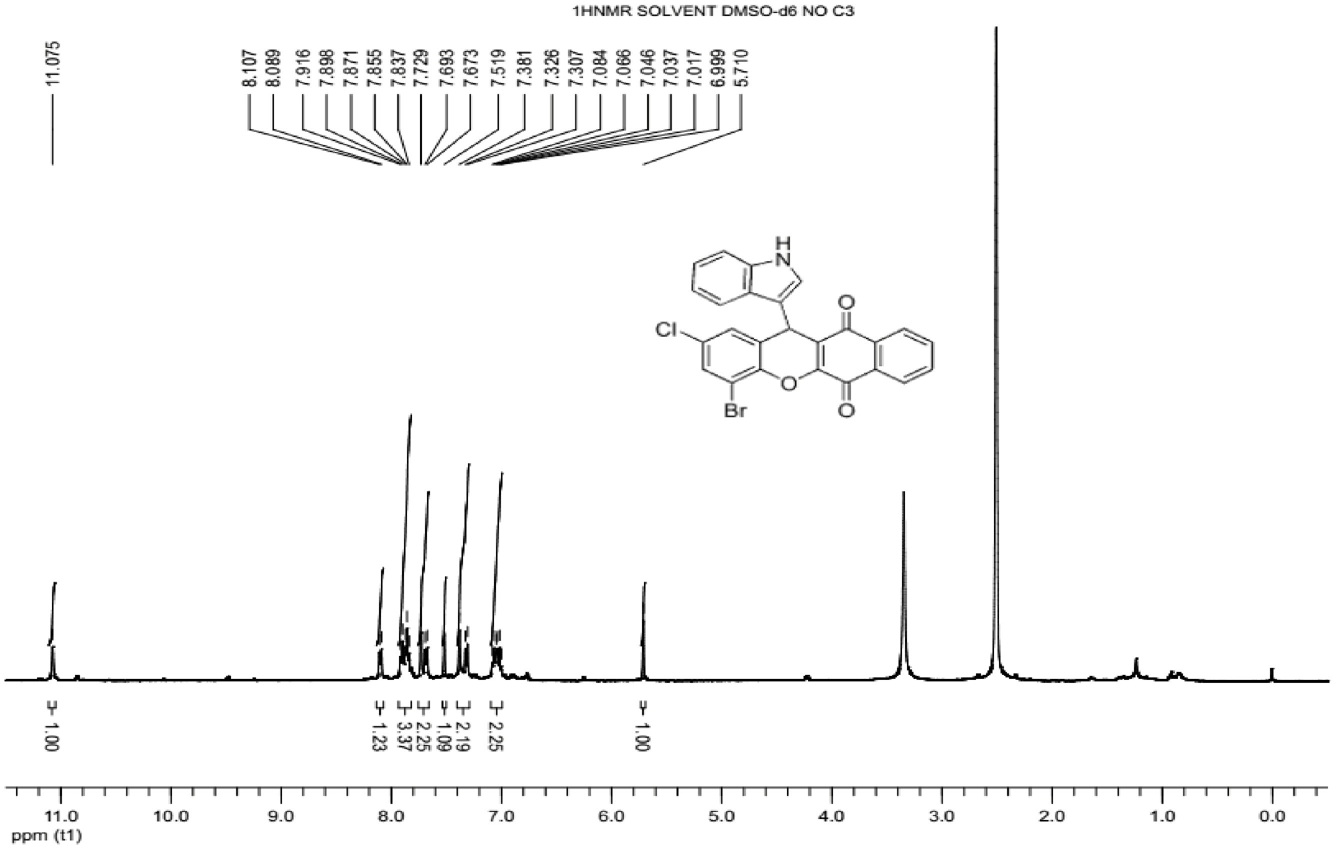

Figure S39 [1] HNMR of **4j**

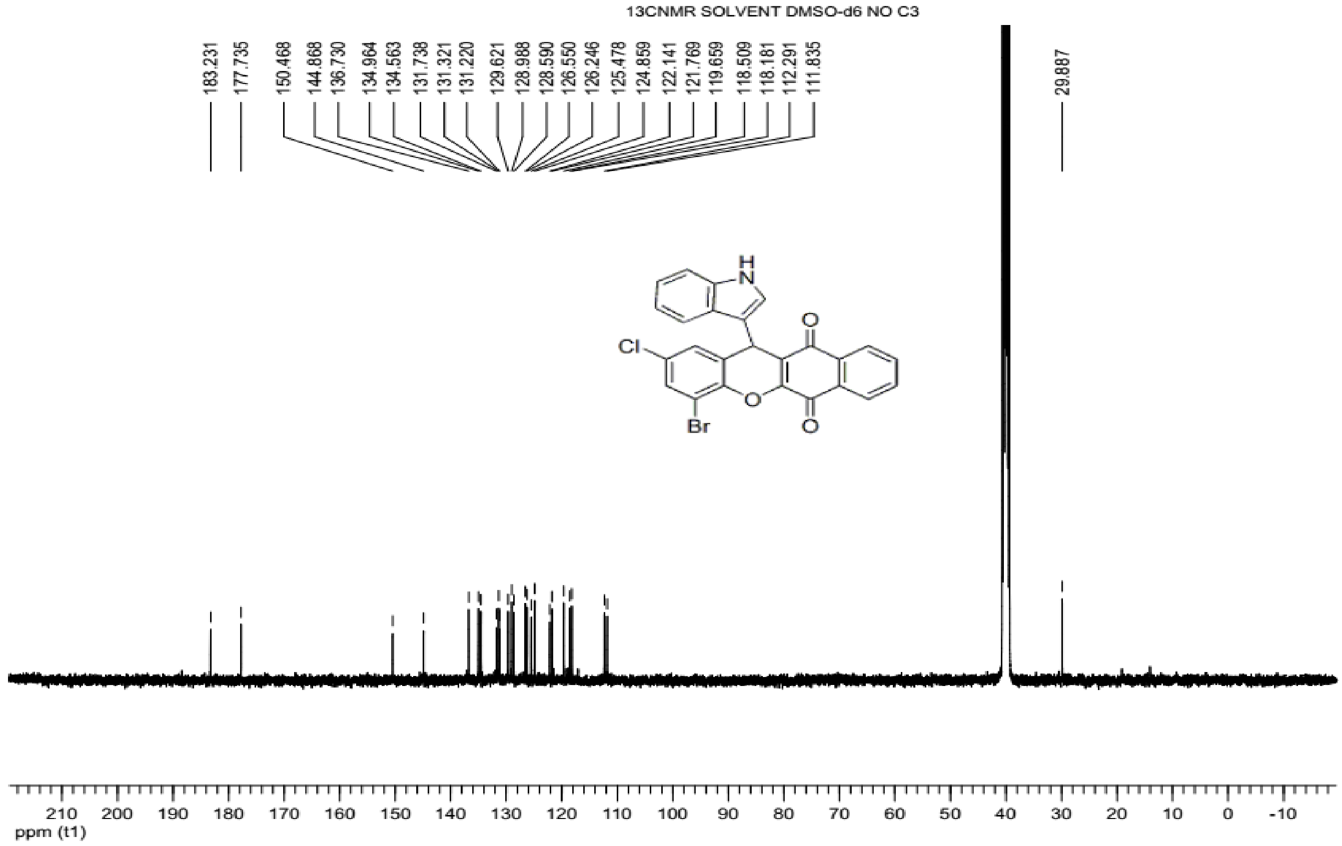

Figure S40 ^{13}C NMR**4j**

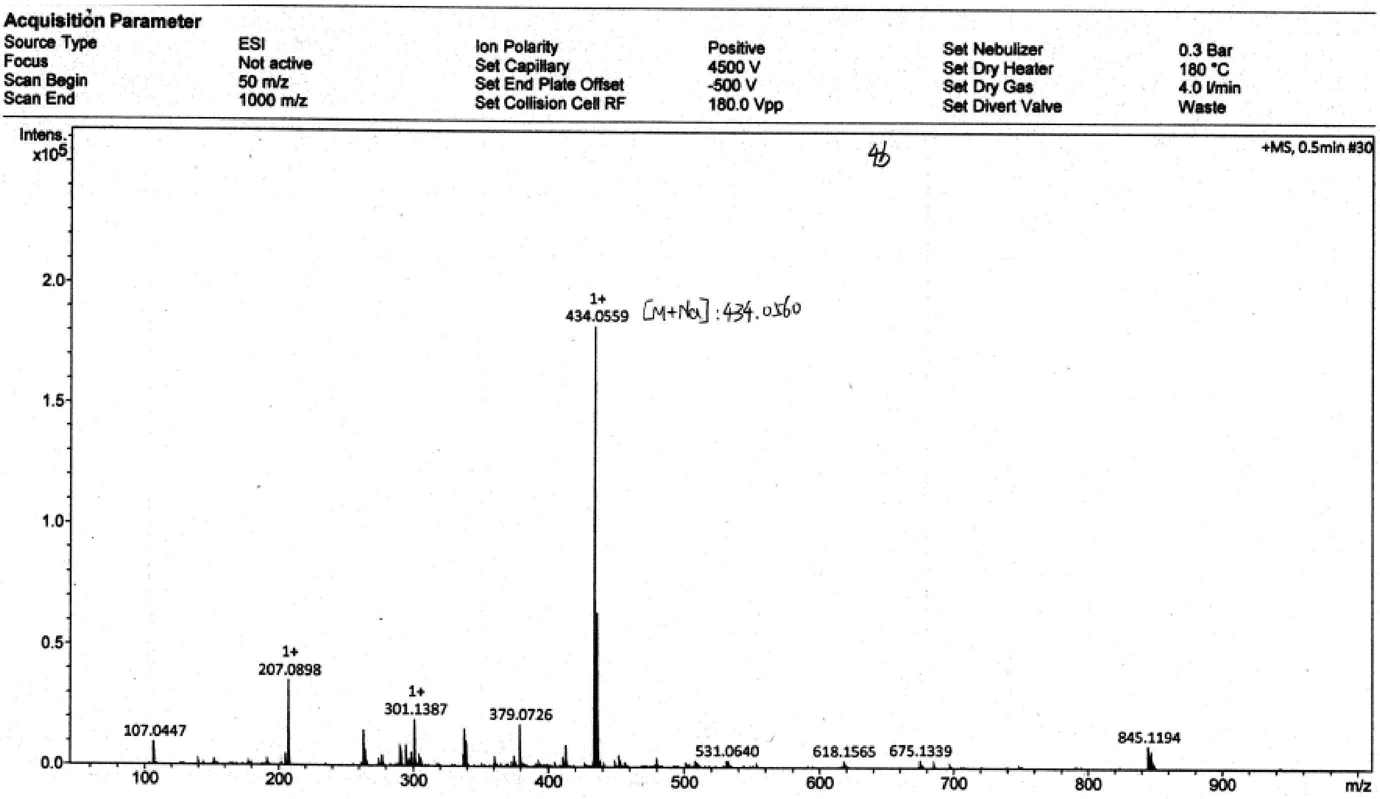

Figure S41 HRMS of **4k**

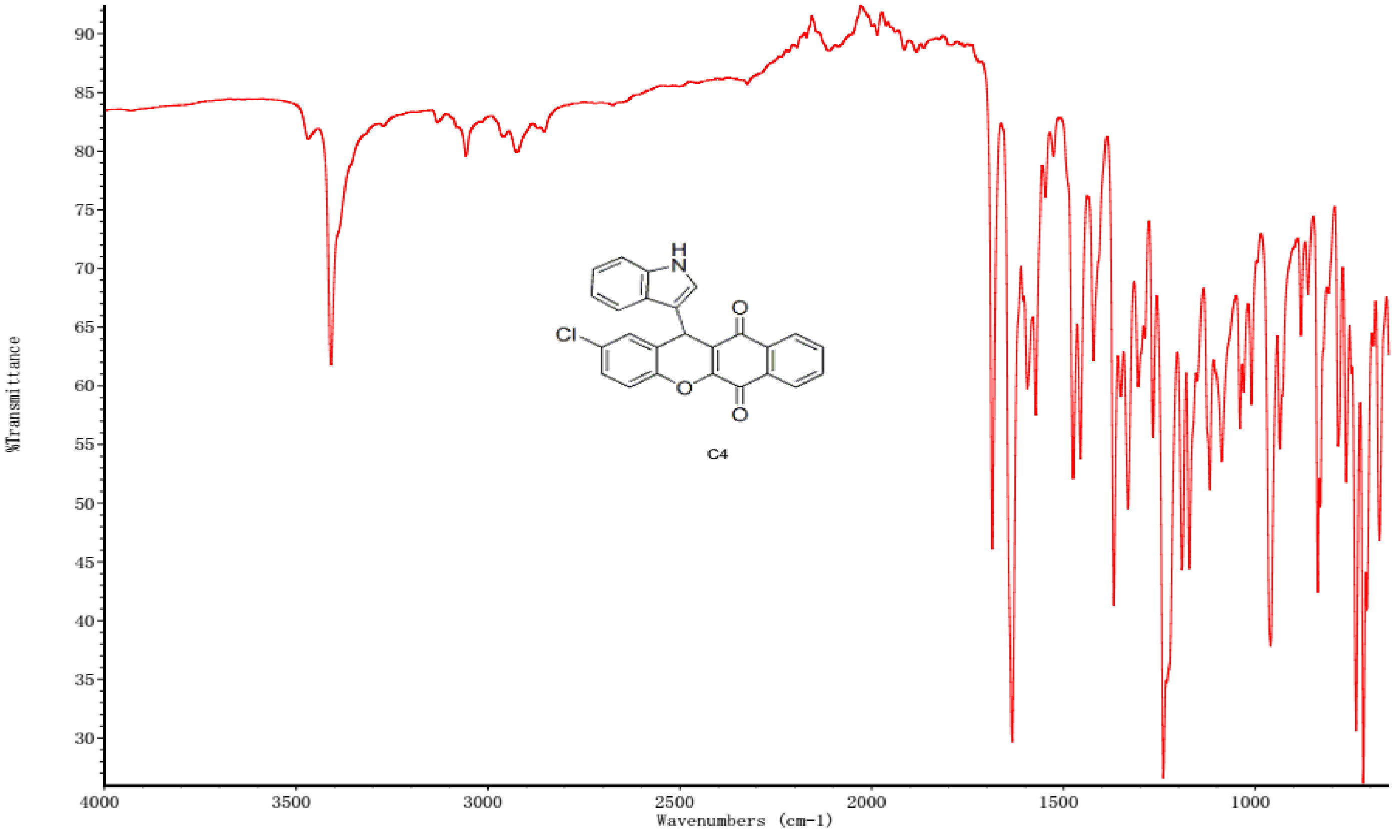

Figure S42 IR of **4k**

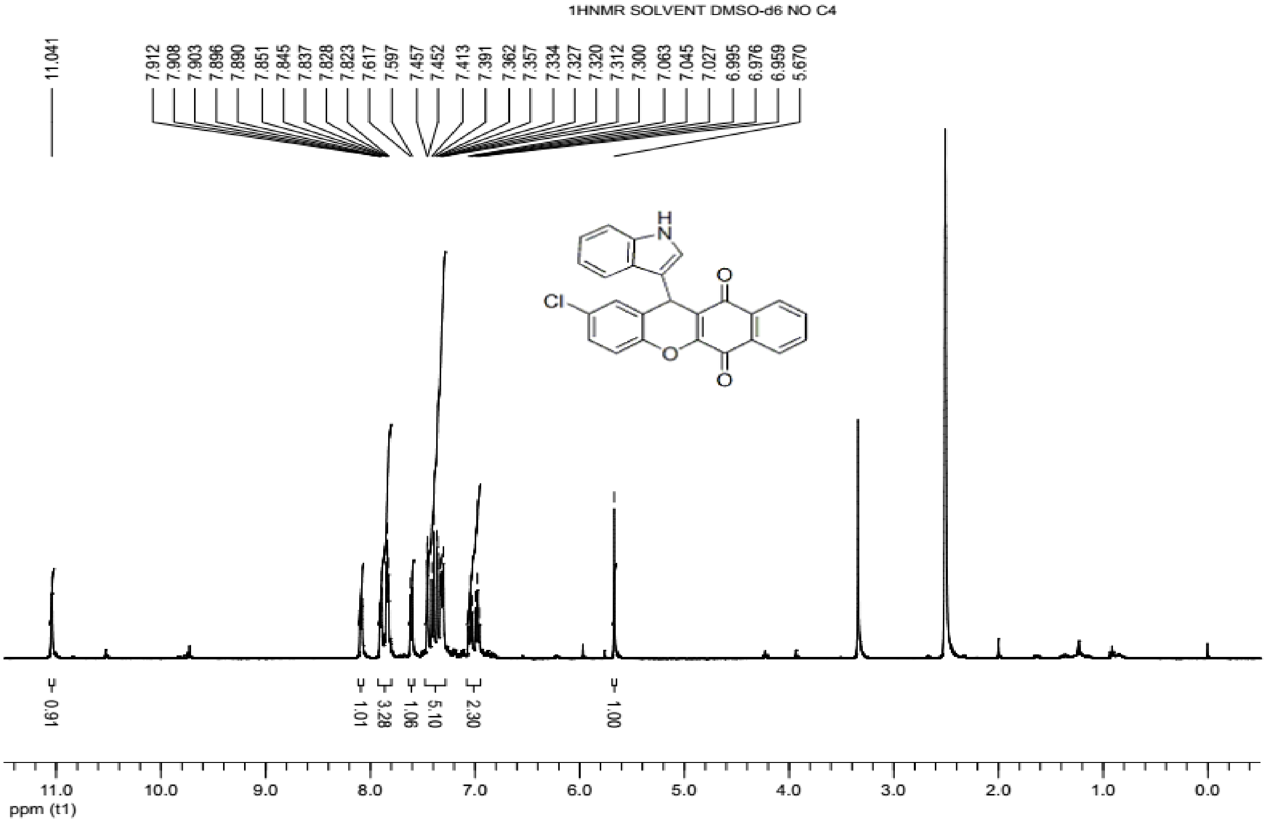

Figure S43 ^{1}H NMR of **4k**

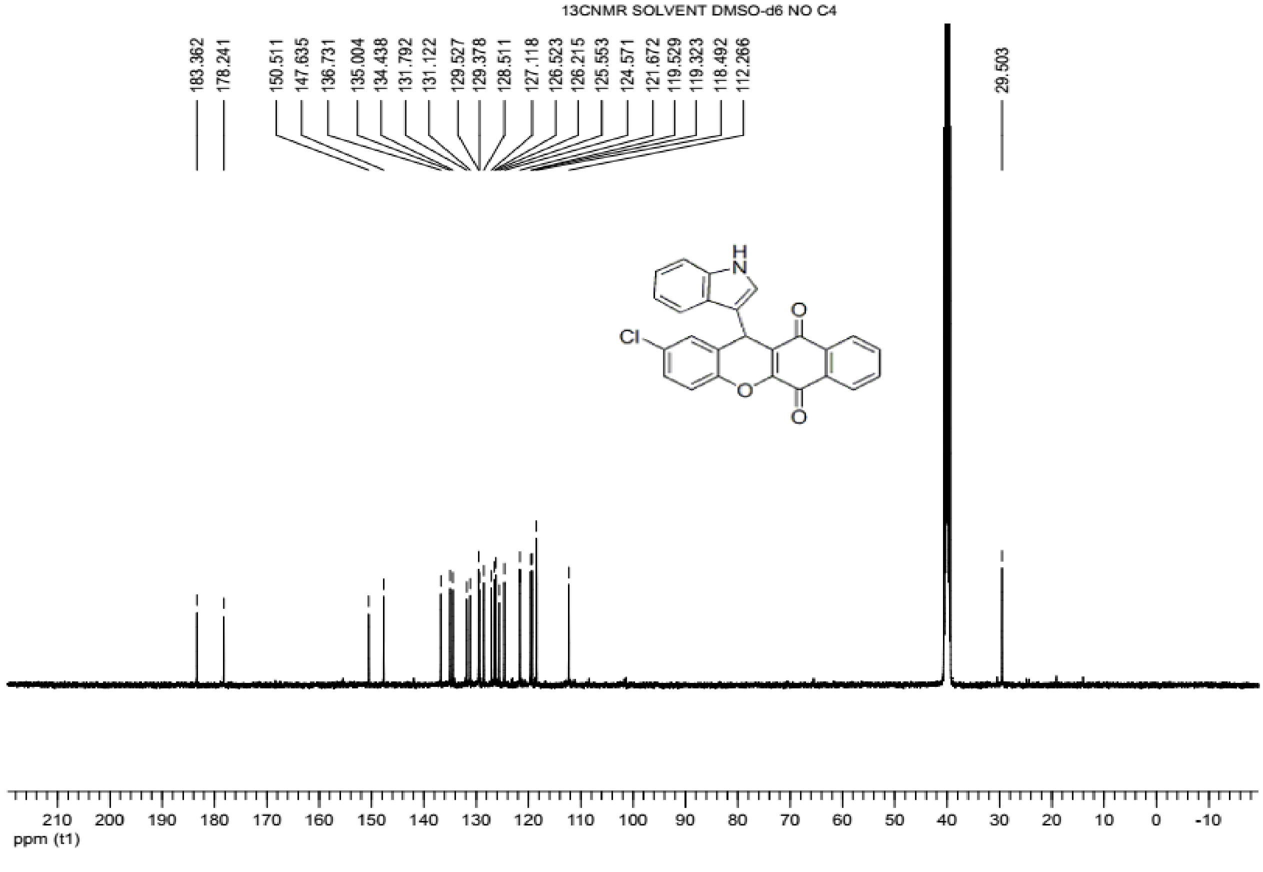

Figure S44 ^{13}C NMR of **4k**

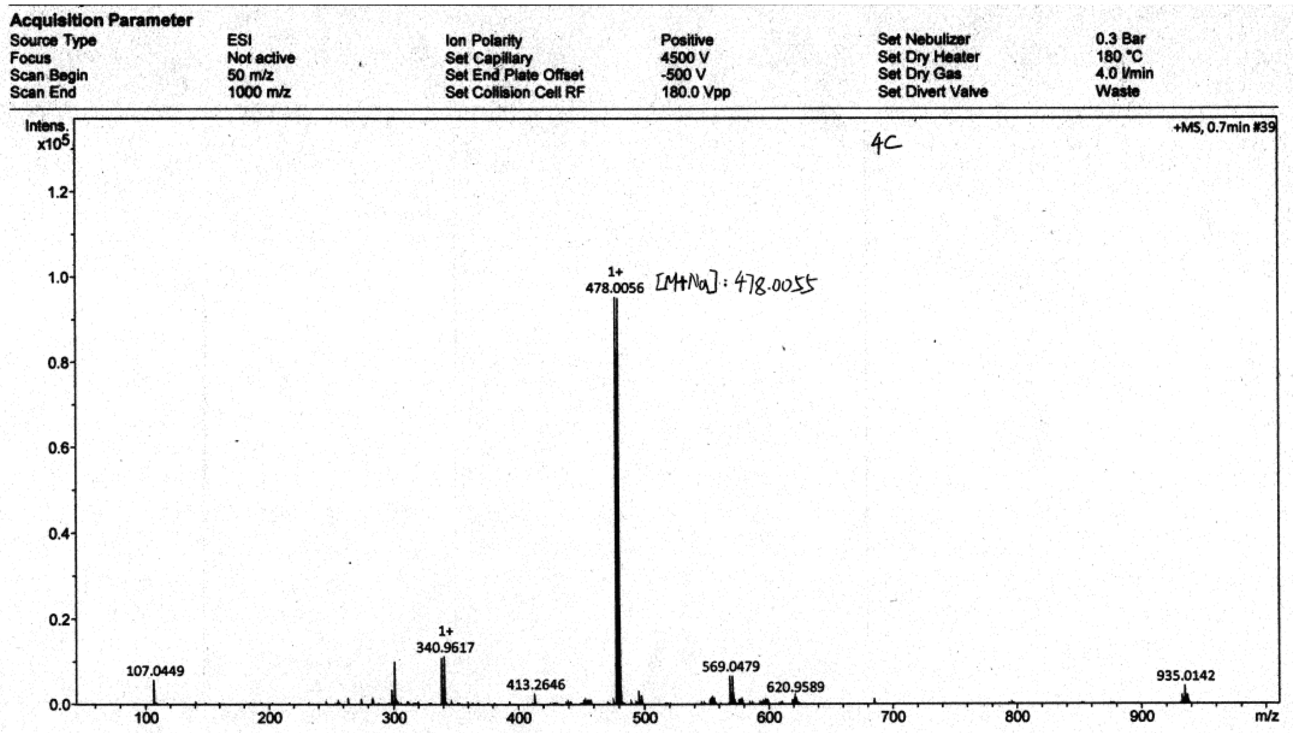

Figure S45 HRMS of **4l**

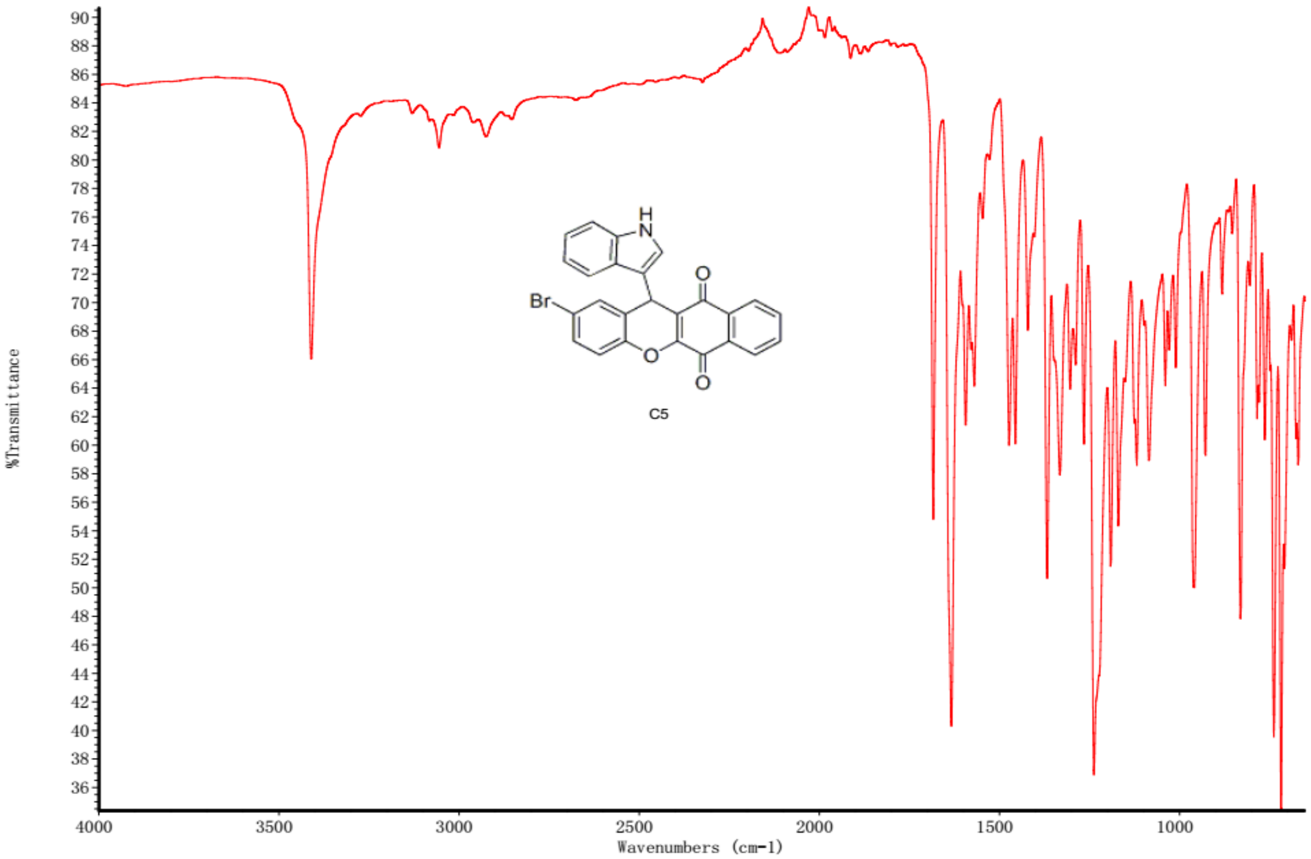

Figure S46 IR of **4l**

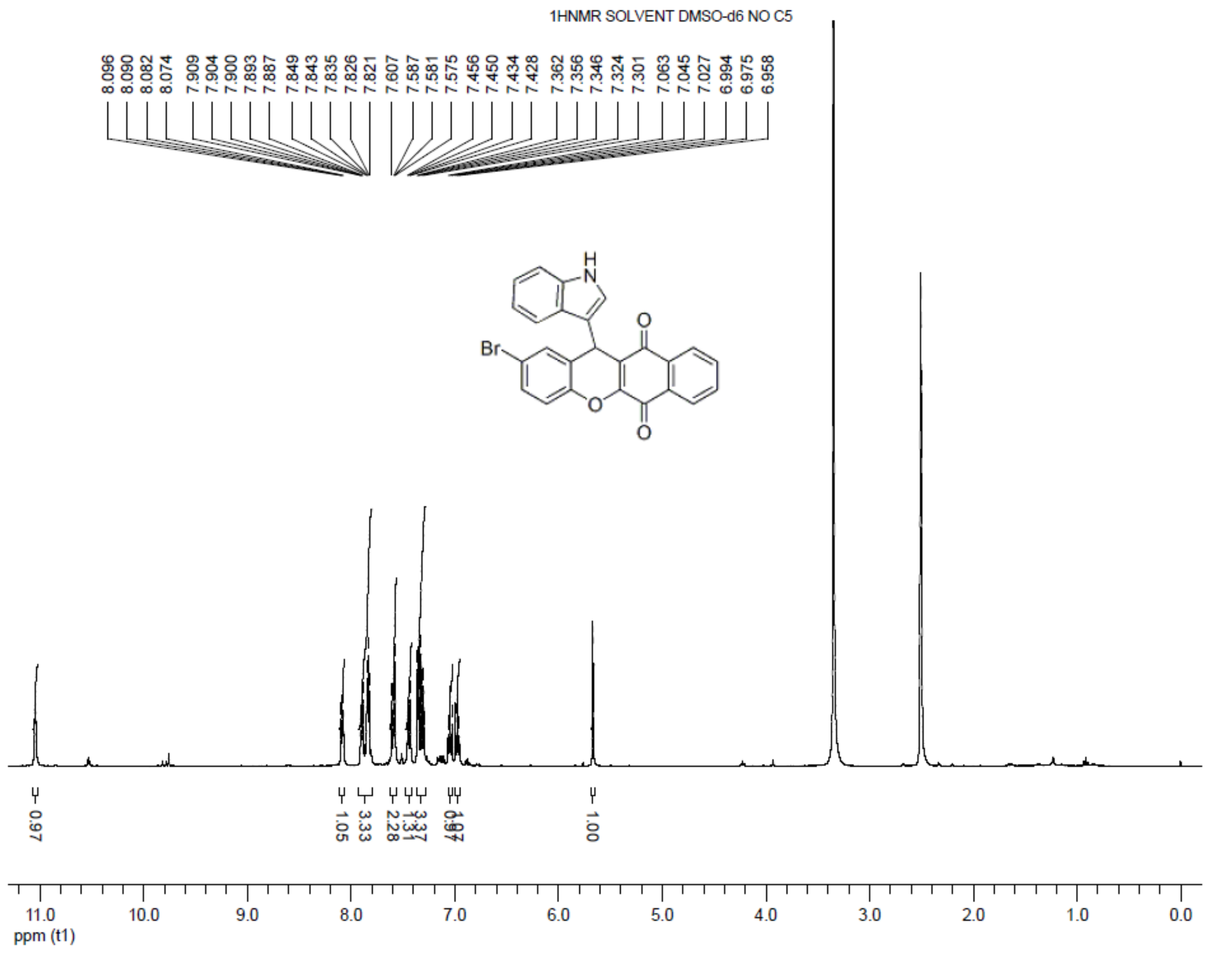

Figure S47 ^{1}H NMR of **4l**

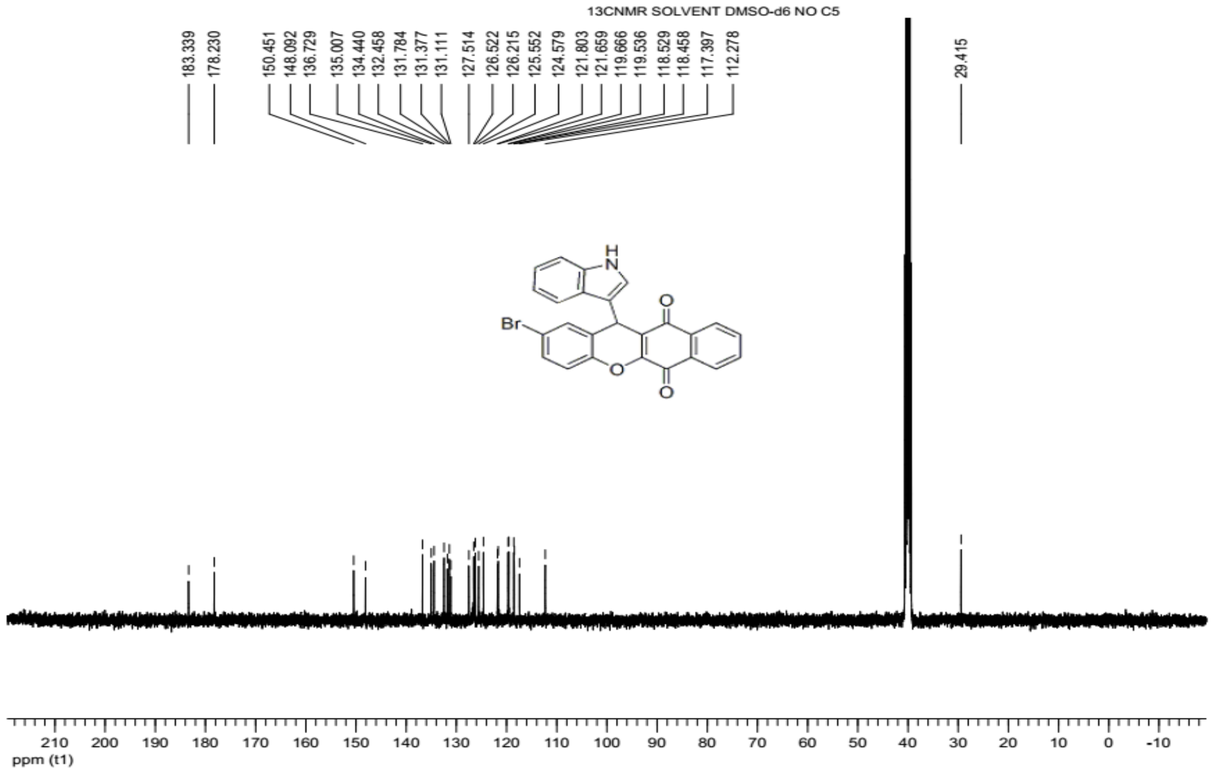

Figure S48 ^{13}C NMR of **4l**

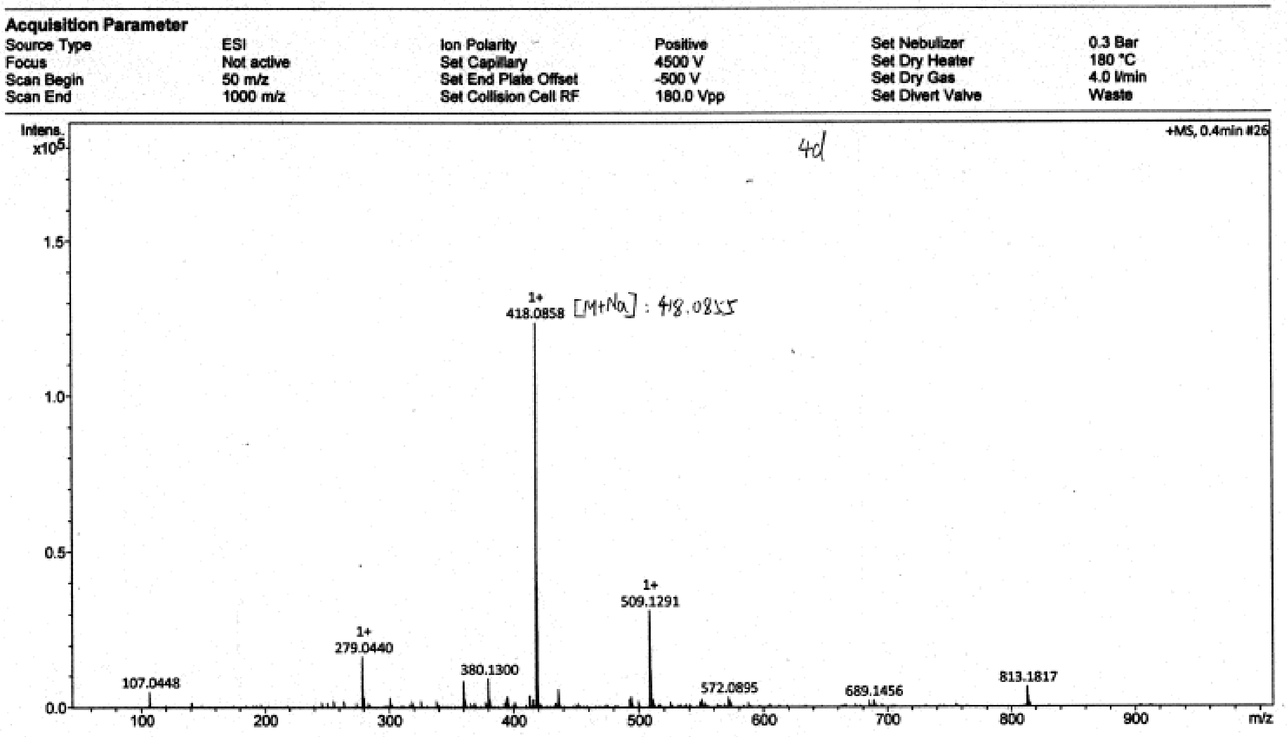

Figure S49 HRMS of **4m**

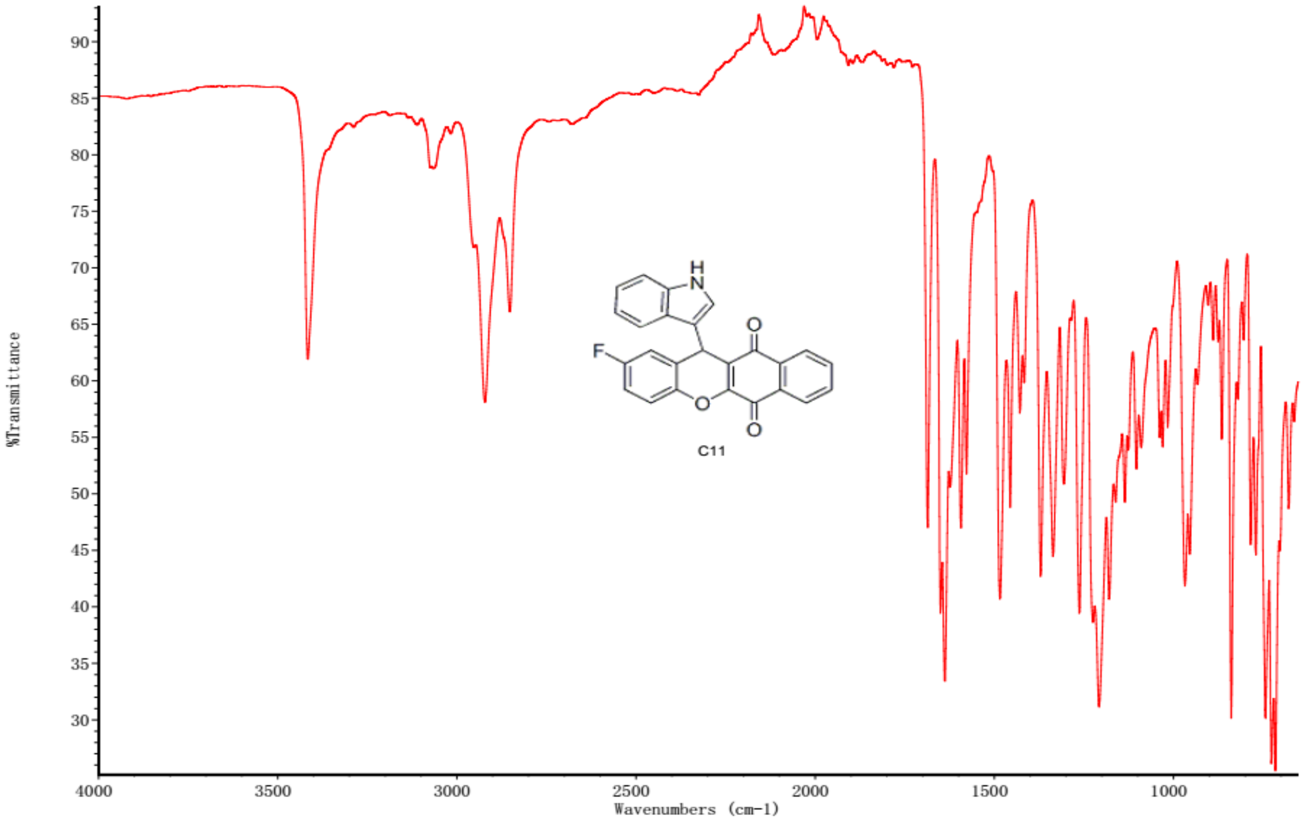

Figure S50 IR of **4m**

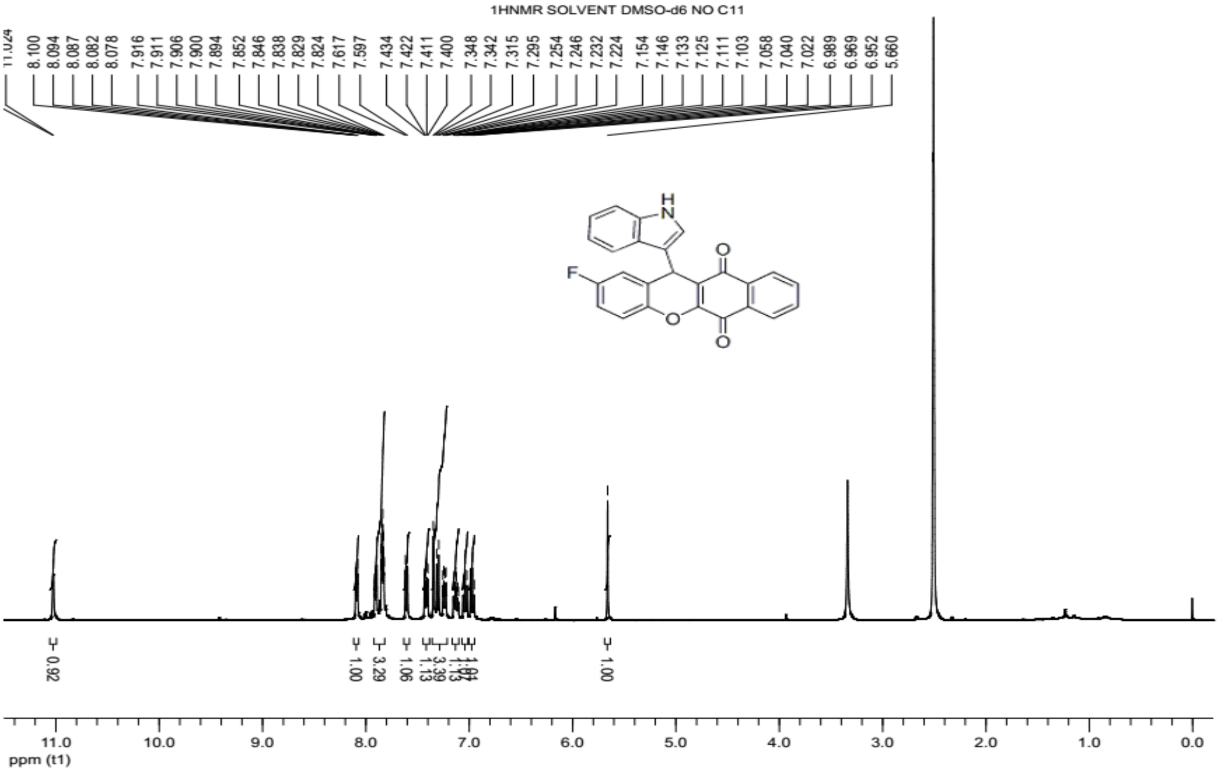

FigureS51 ^{1}H NMR of **4m**

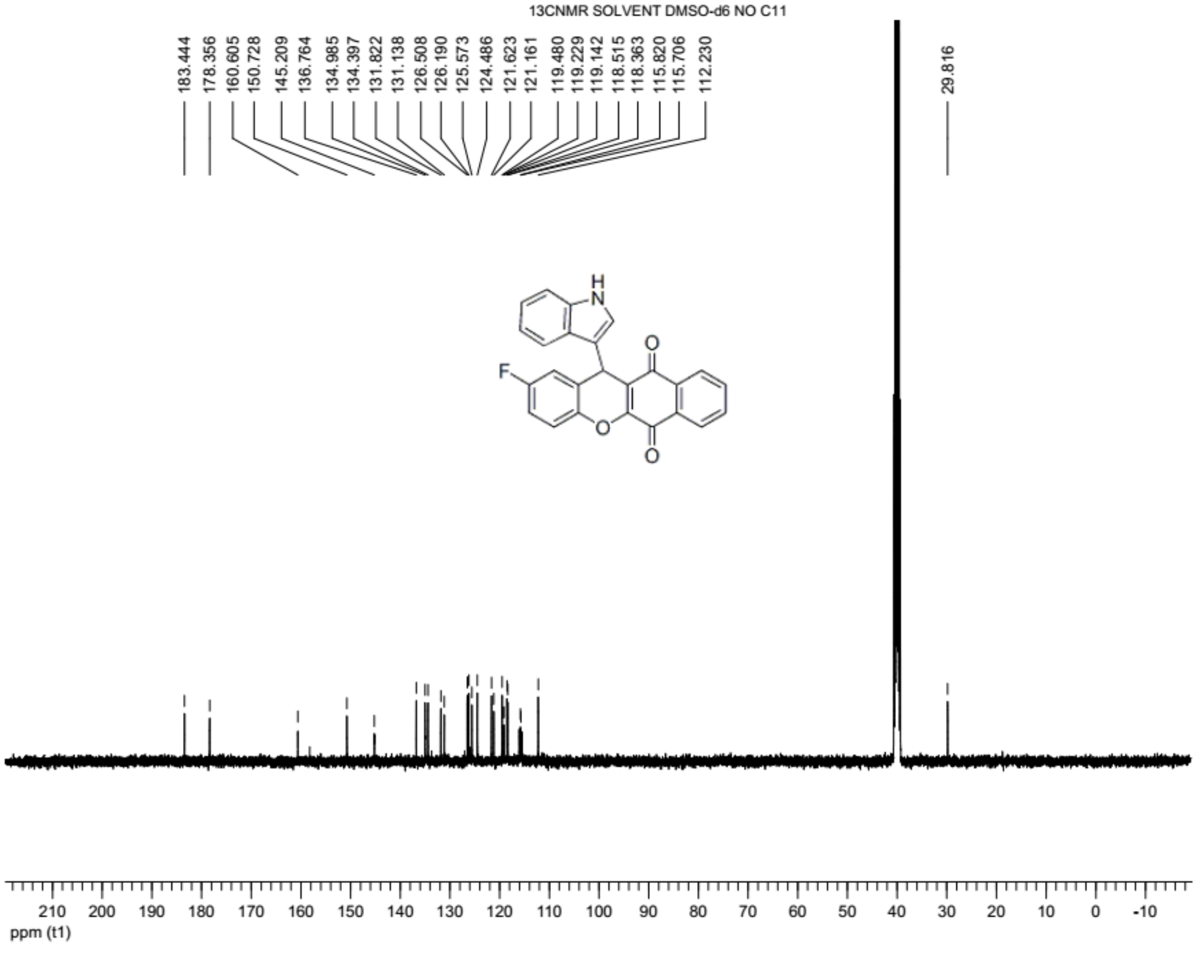

Figure S52 ^{13}CNMR of **4m**

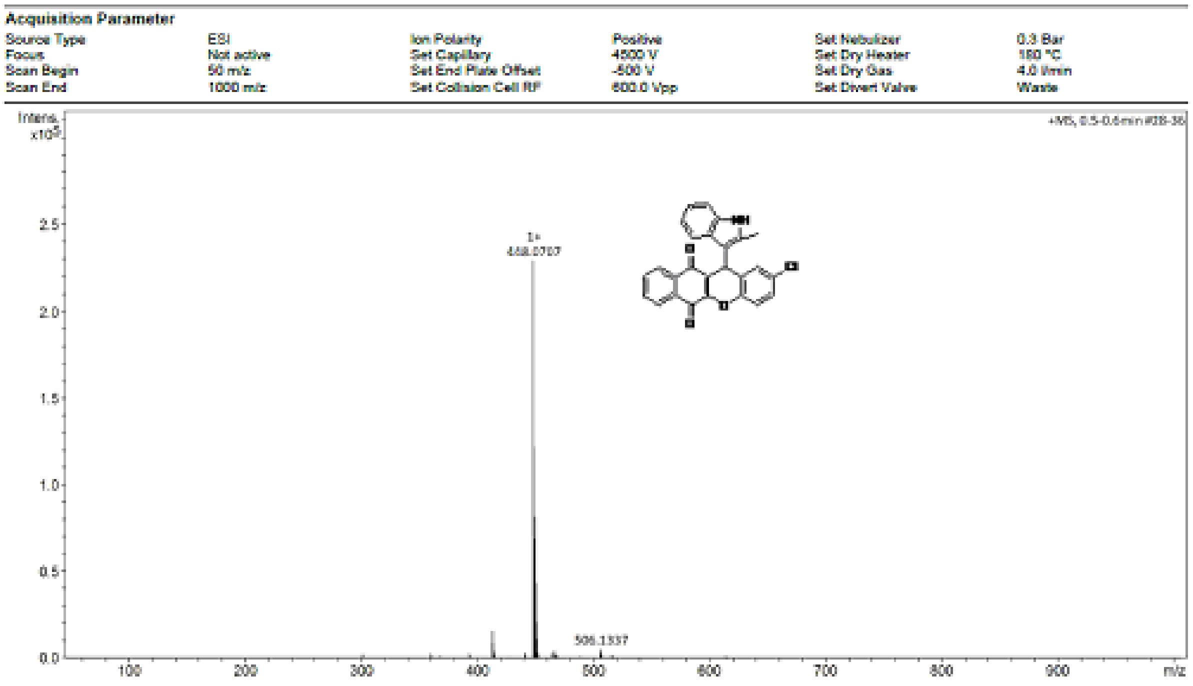

Figure S53 HRMS of **4n**

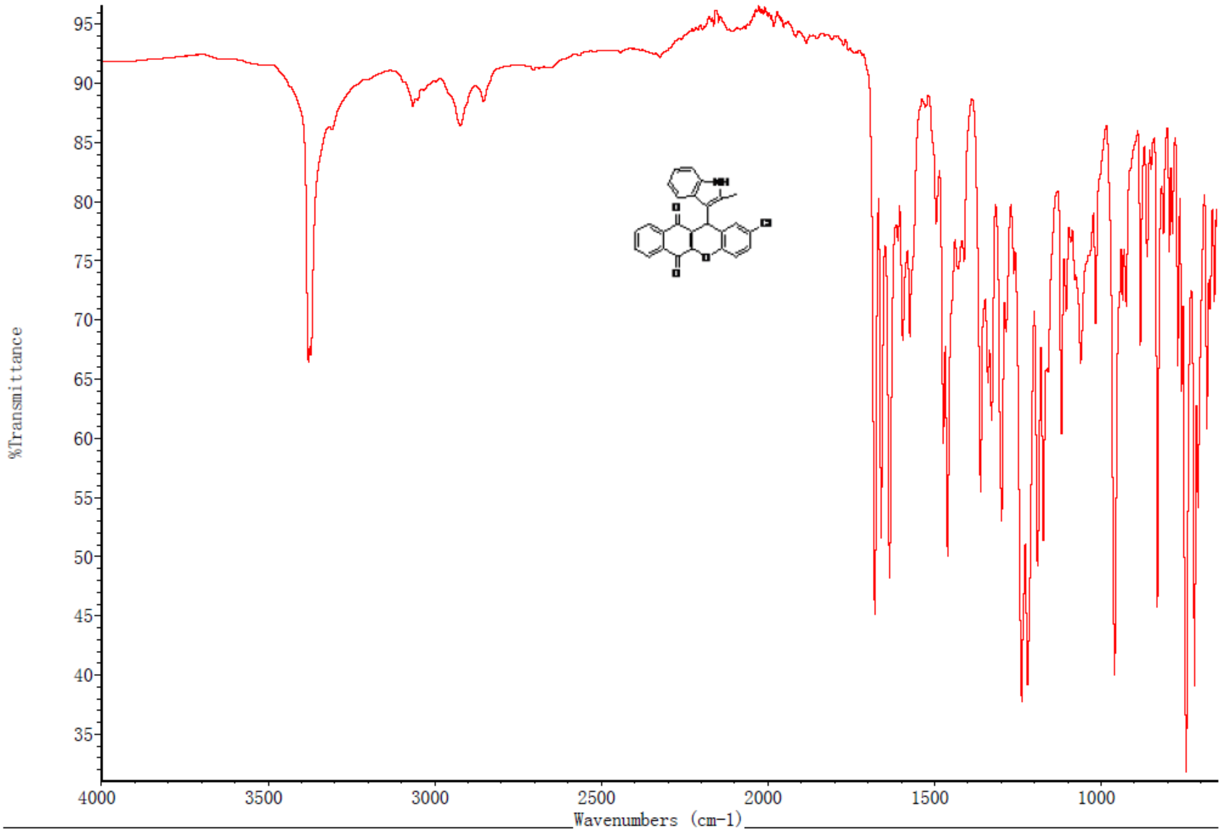

Figure S54 IR of **4n**

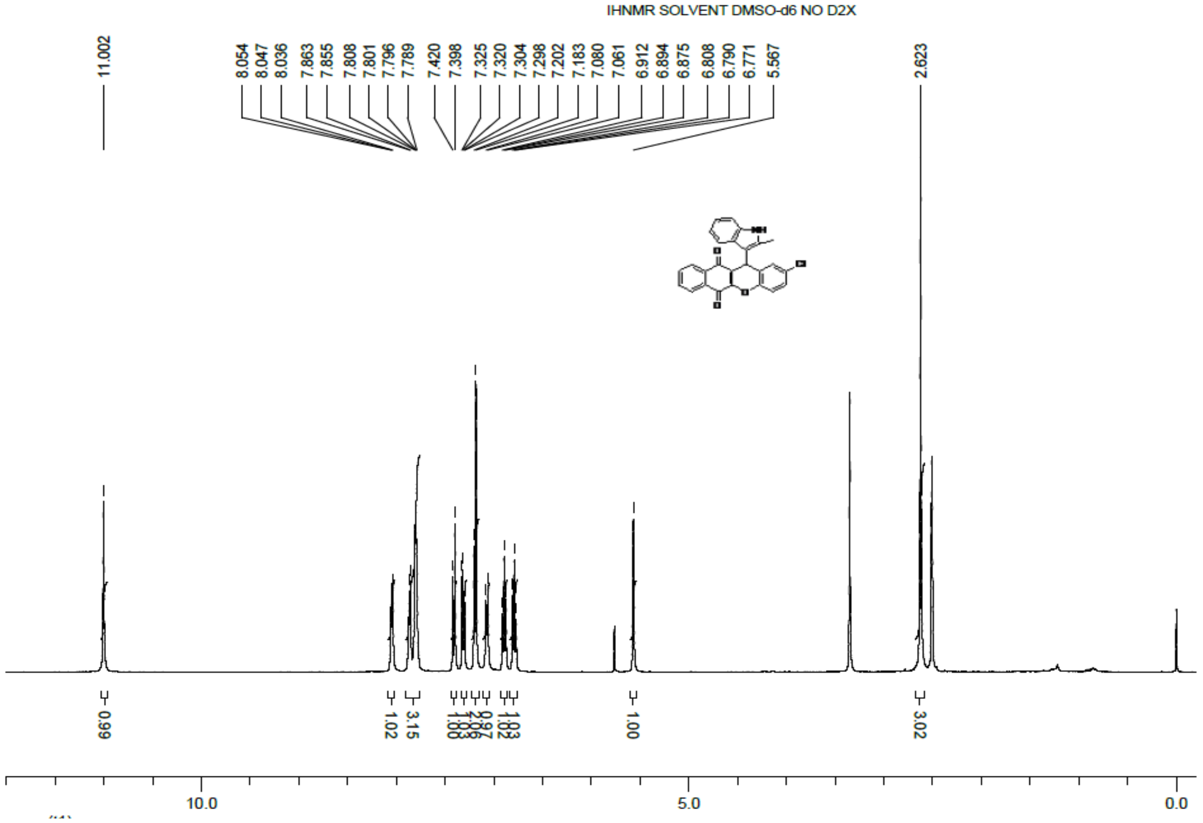

Figure S55 ^{1}H NMR of **4n**

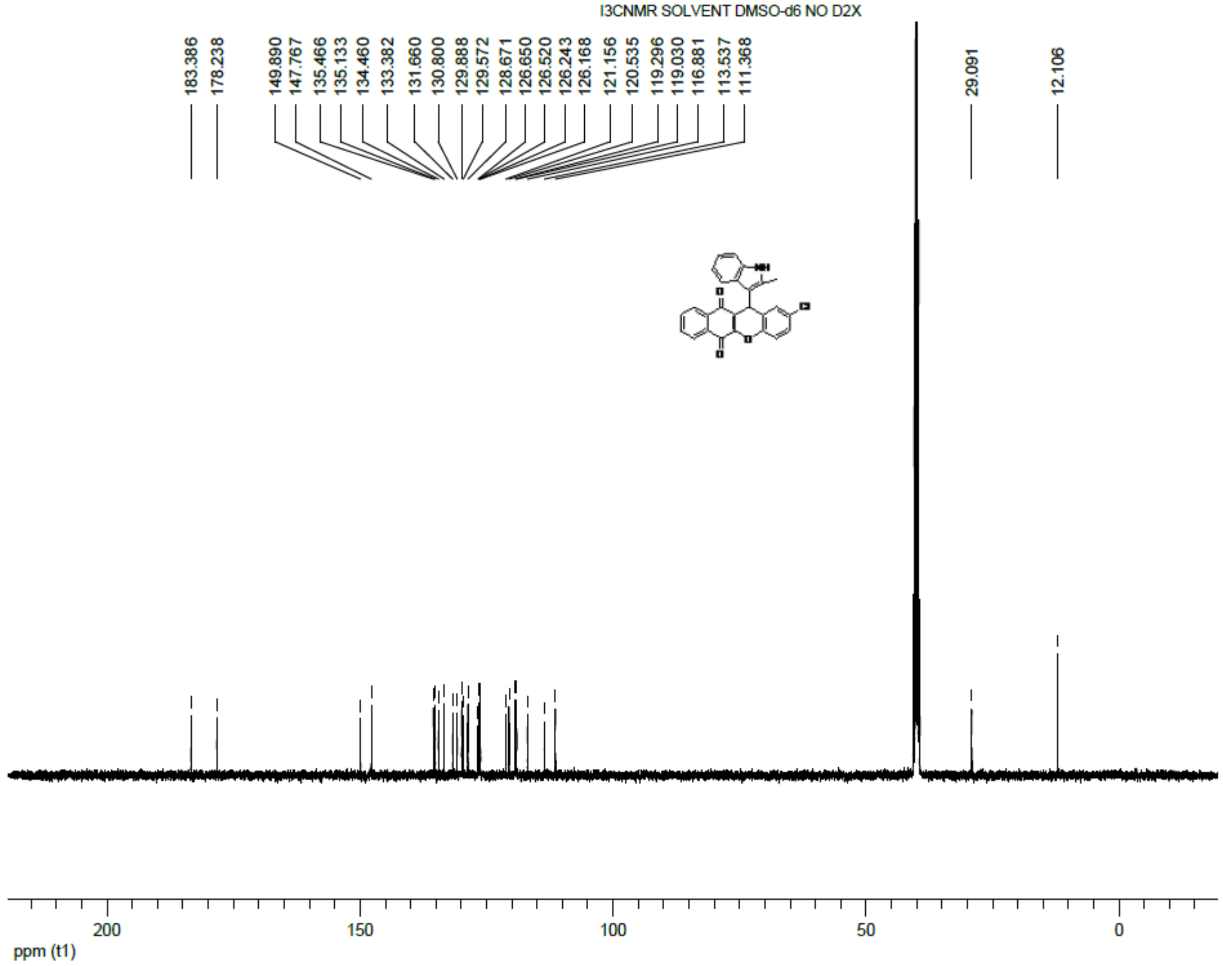

Figure S56 ^{13}CNMR of **4n**

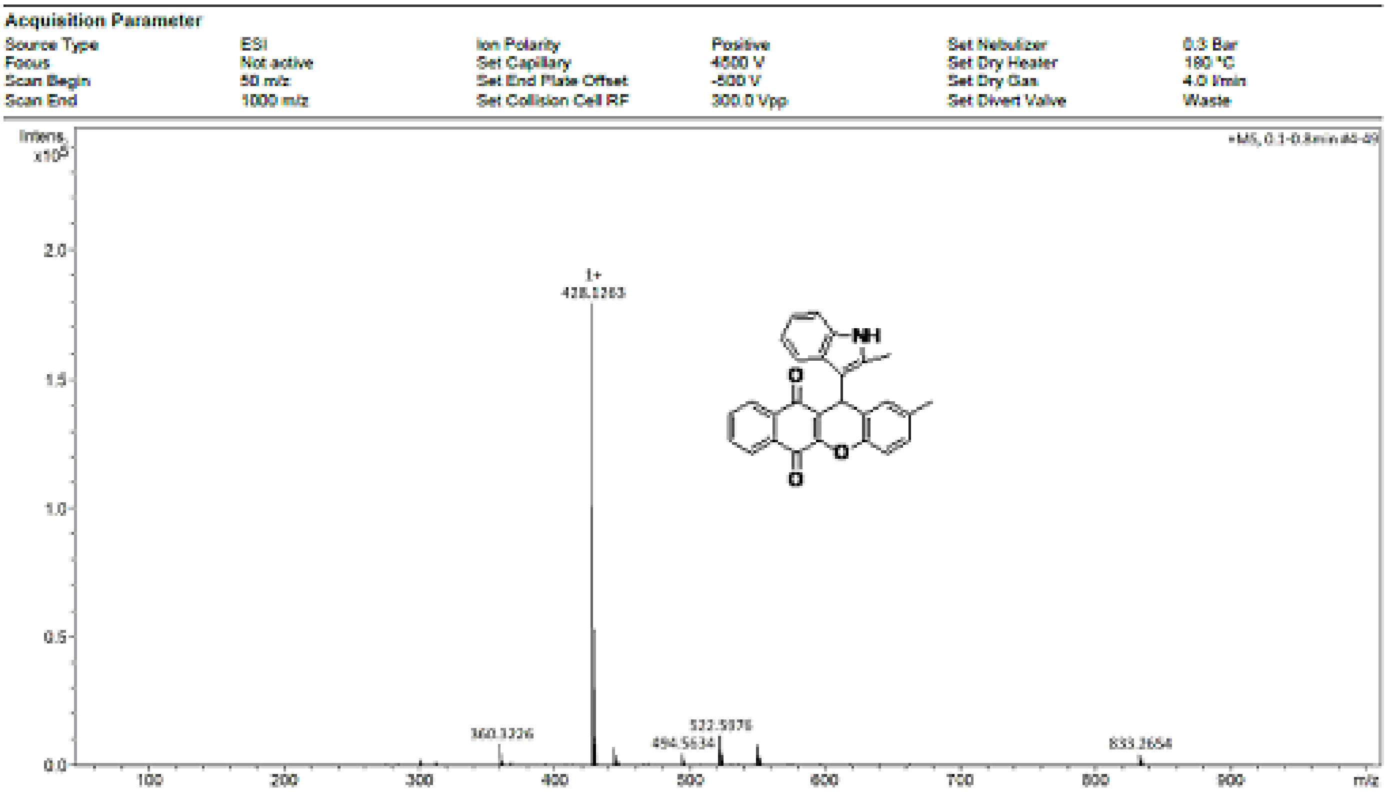

Figure S57 HRMS of **4o**

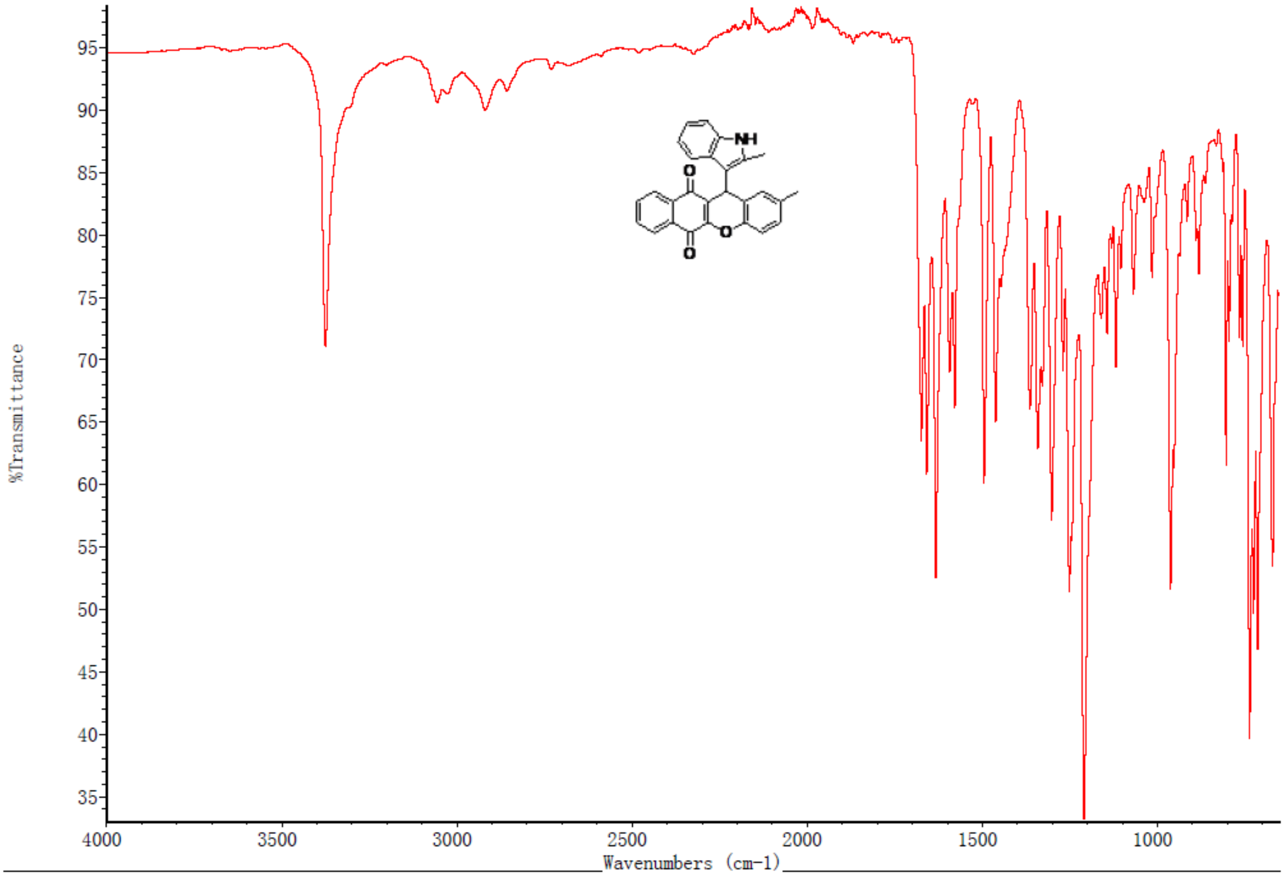

Figure S58 IR of **4o**

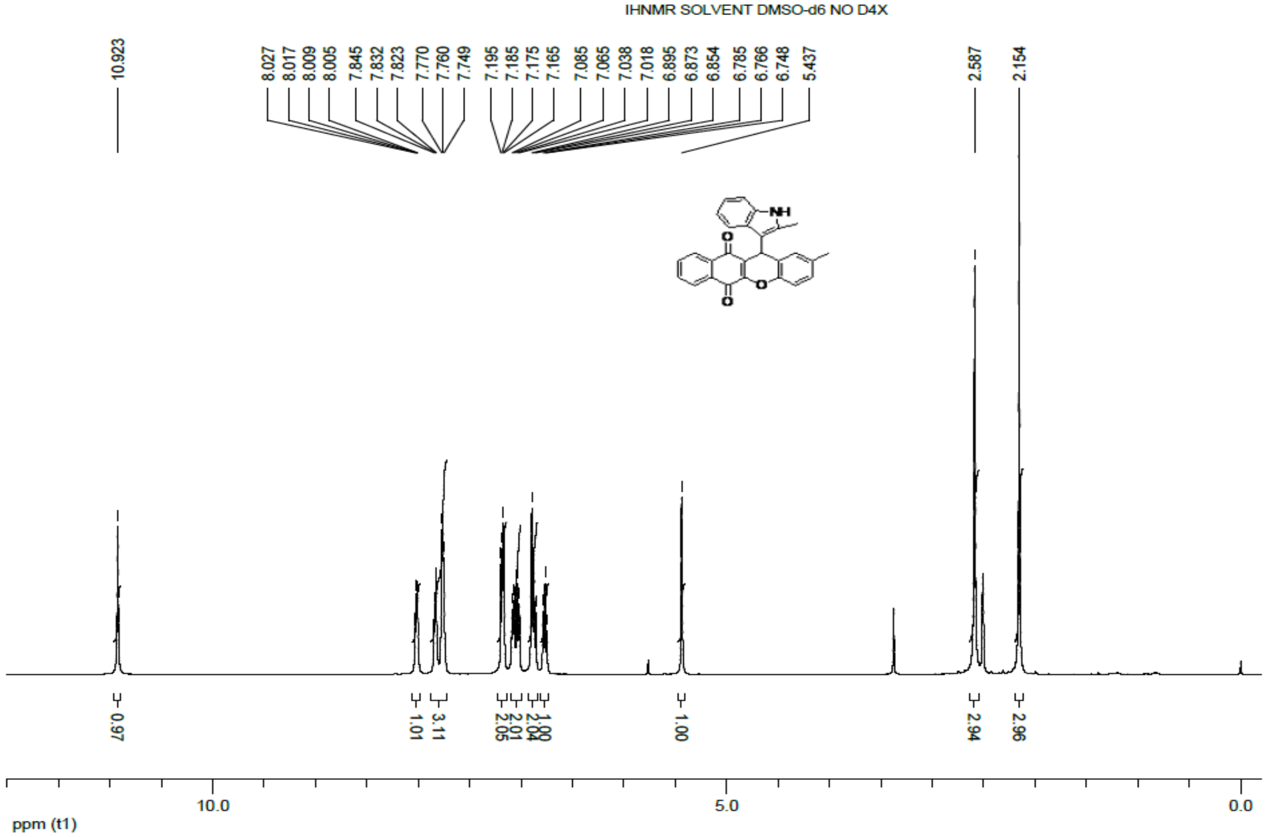

Figure S59 ^{1}H NMR of **4o**

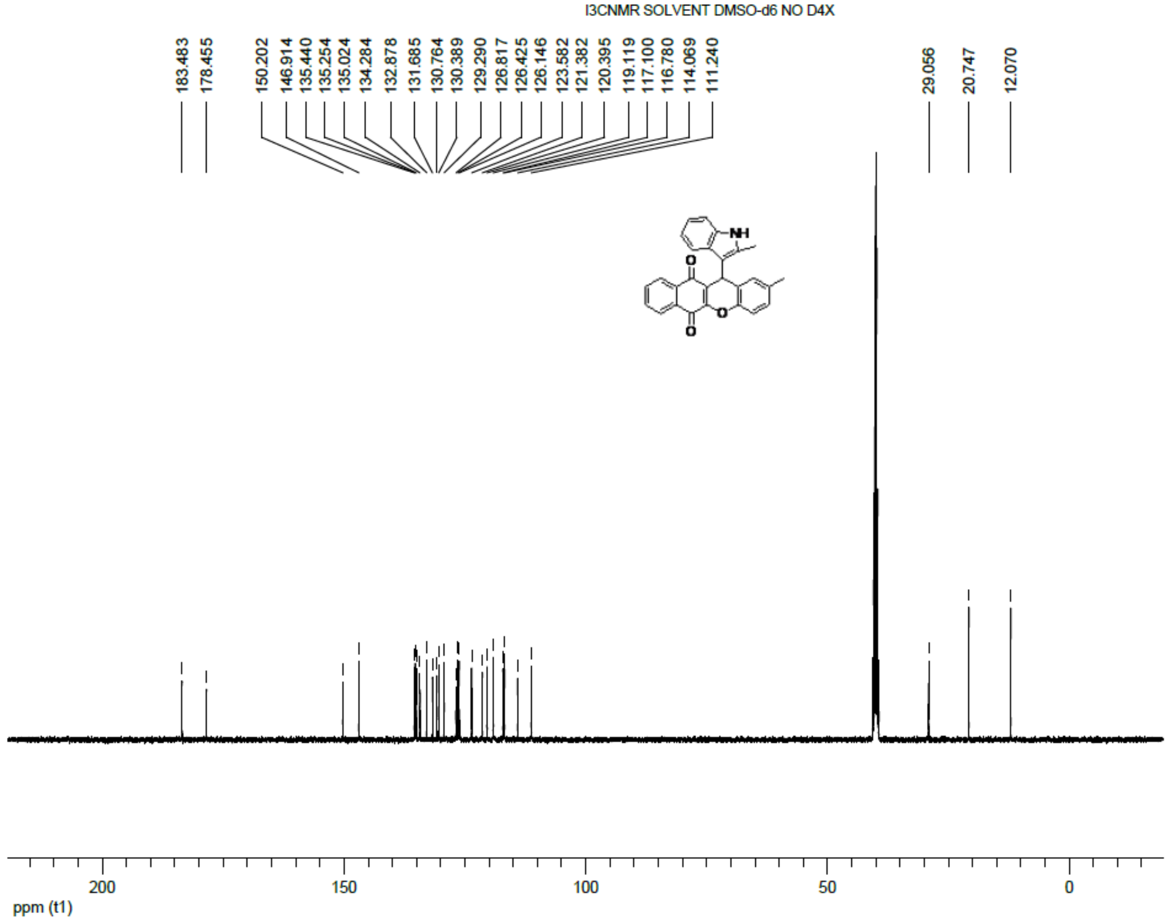

Figure S60 ^{13}CNMR of **4o**

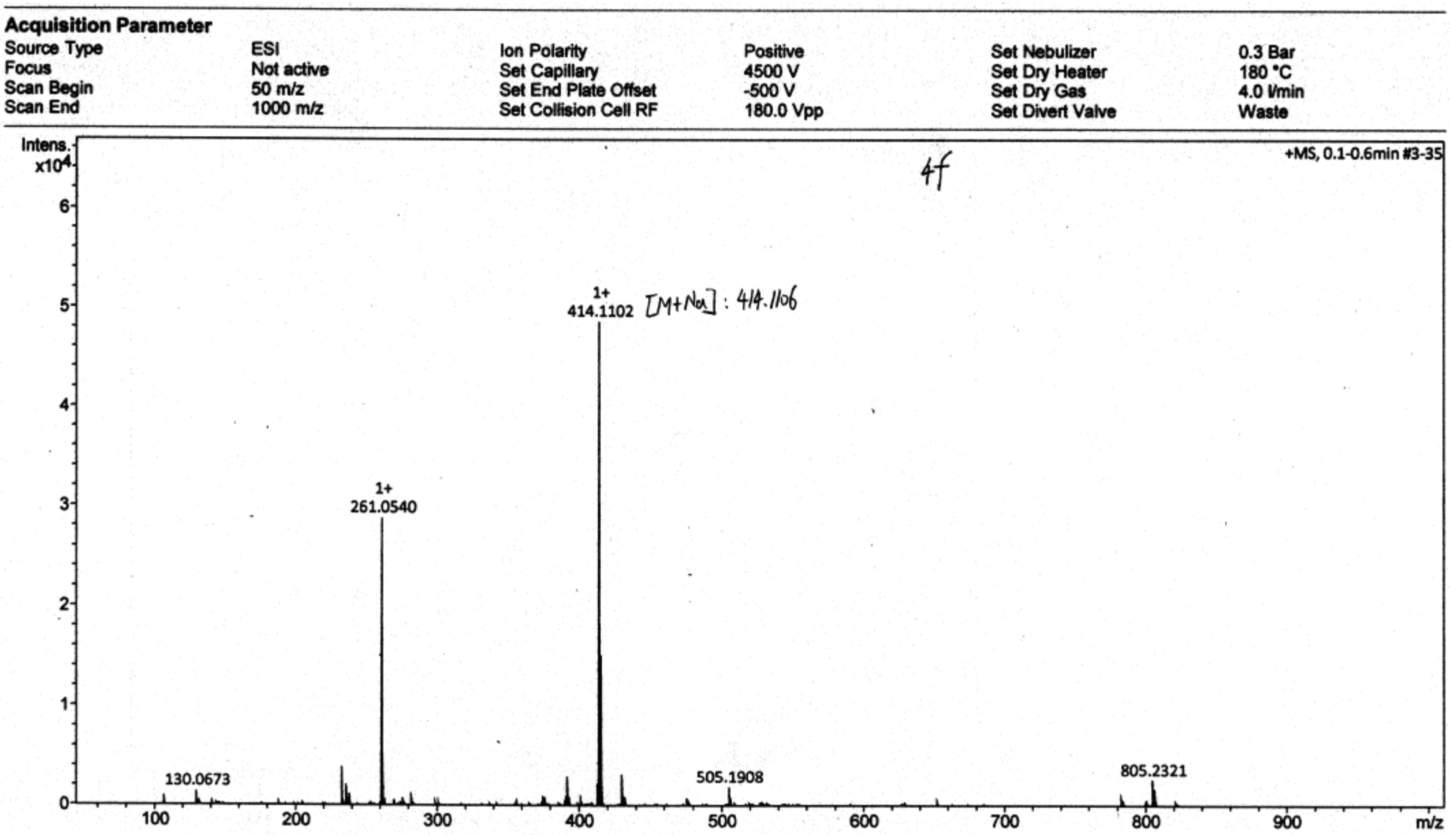

Figure S61 HRMS of **4p**

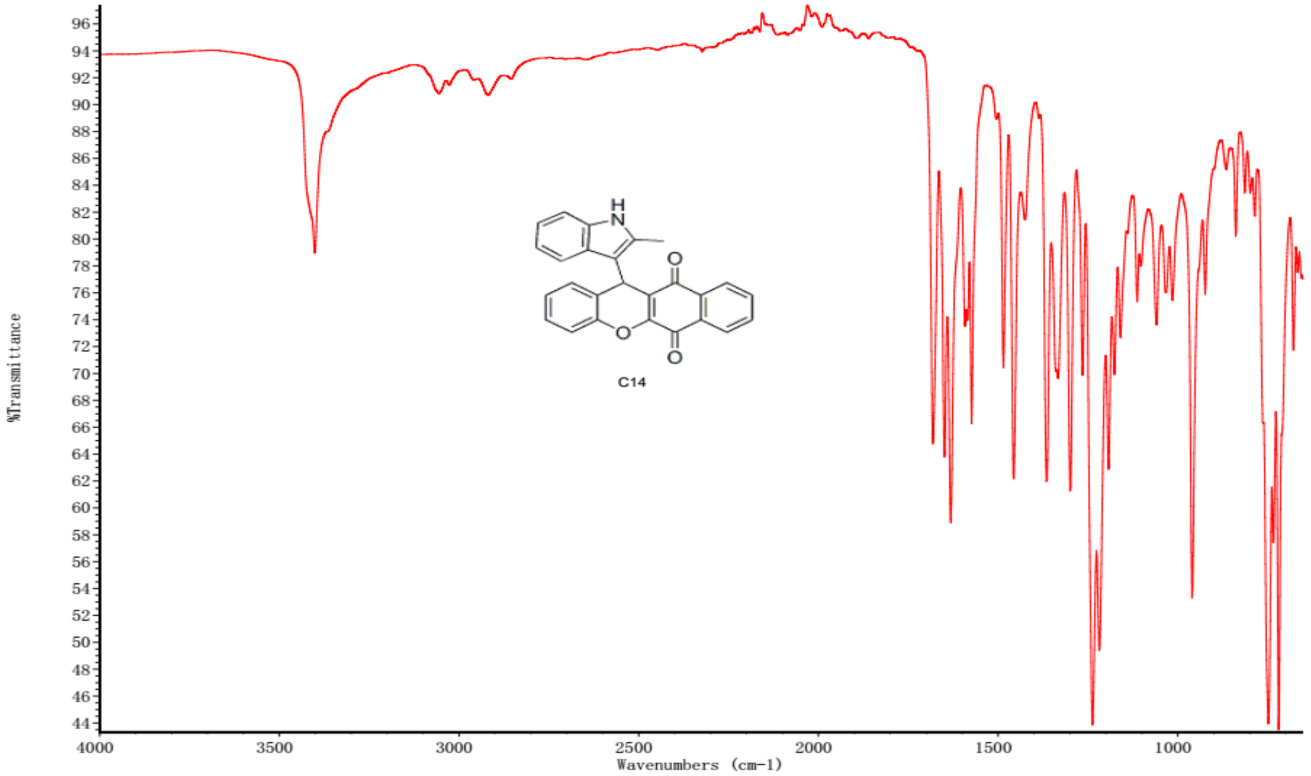

Figure S62 IR of **4p**

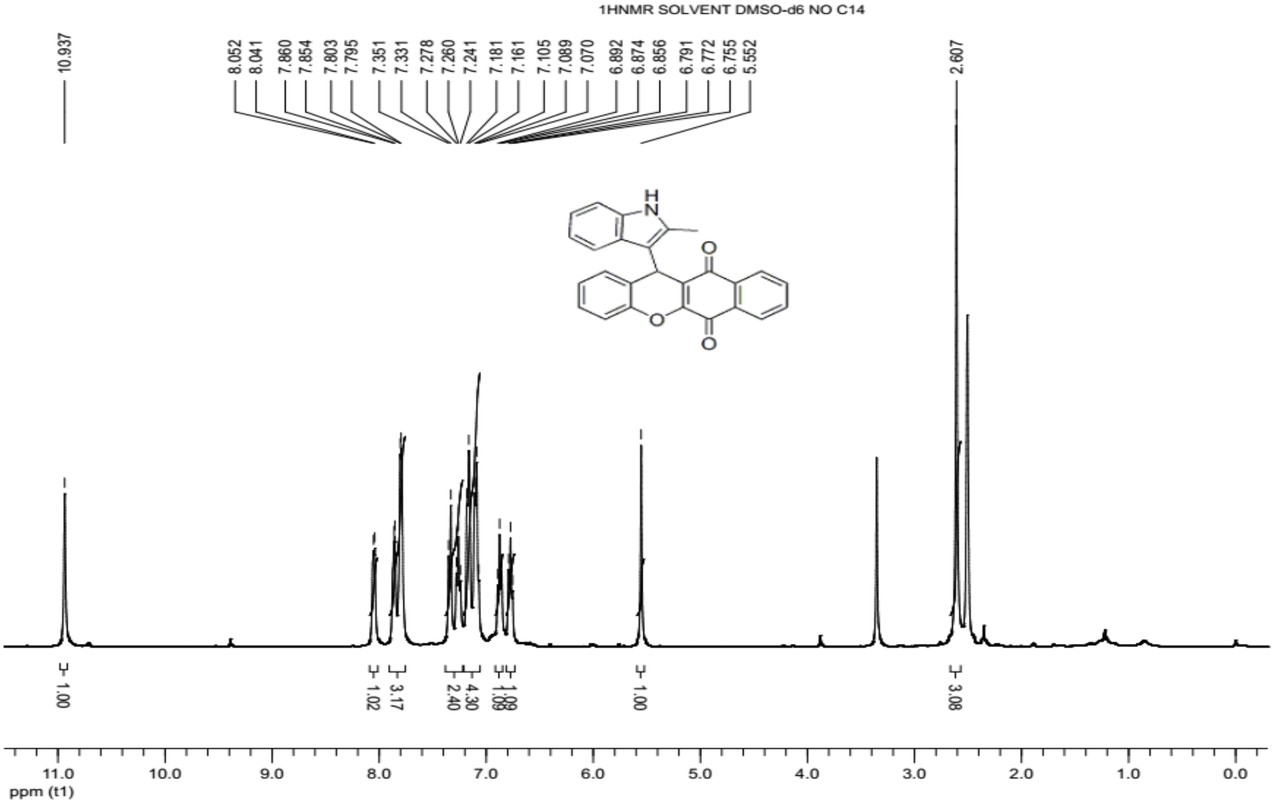

Figure S63 ^{1}H NMR of **4p**

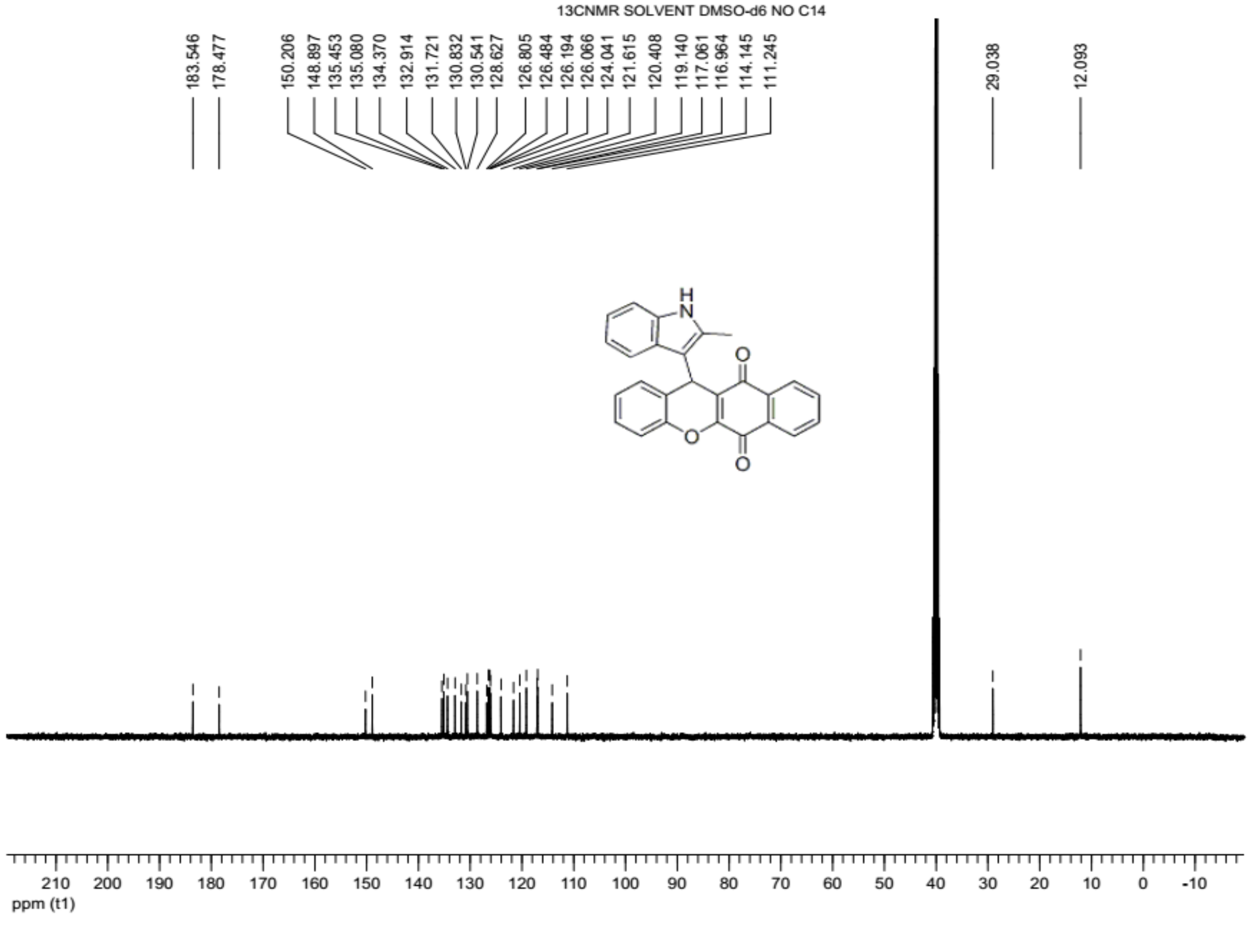

Figure S64 ^{13}C NMR of **4p**

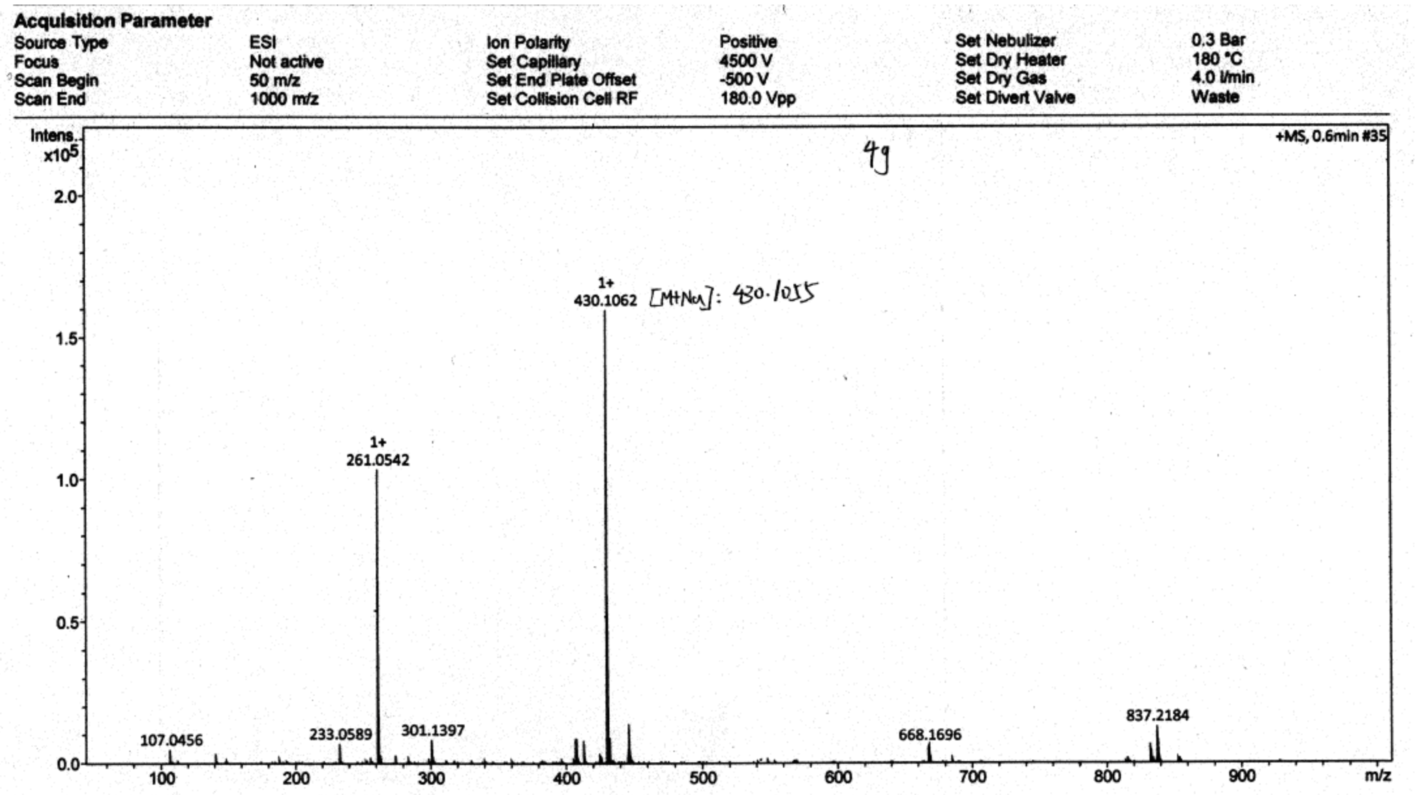

Figure S65 HMRS of **4q**

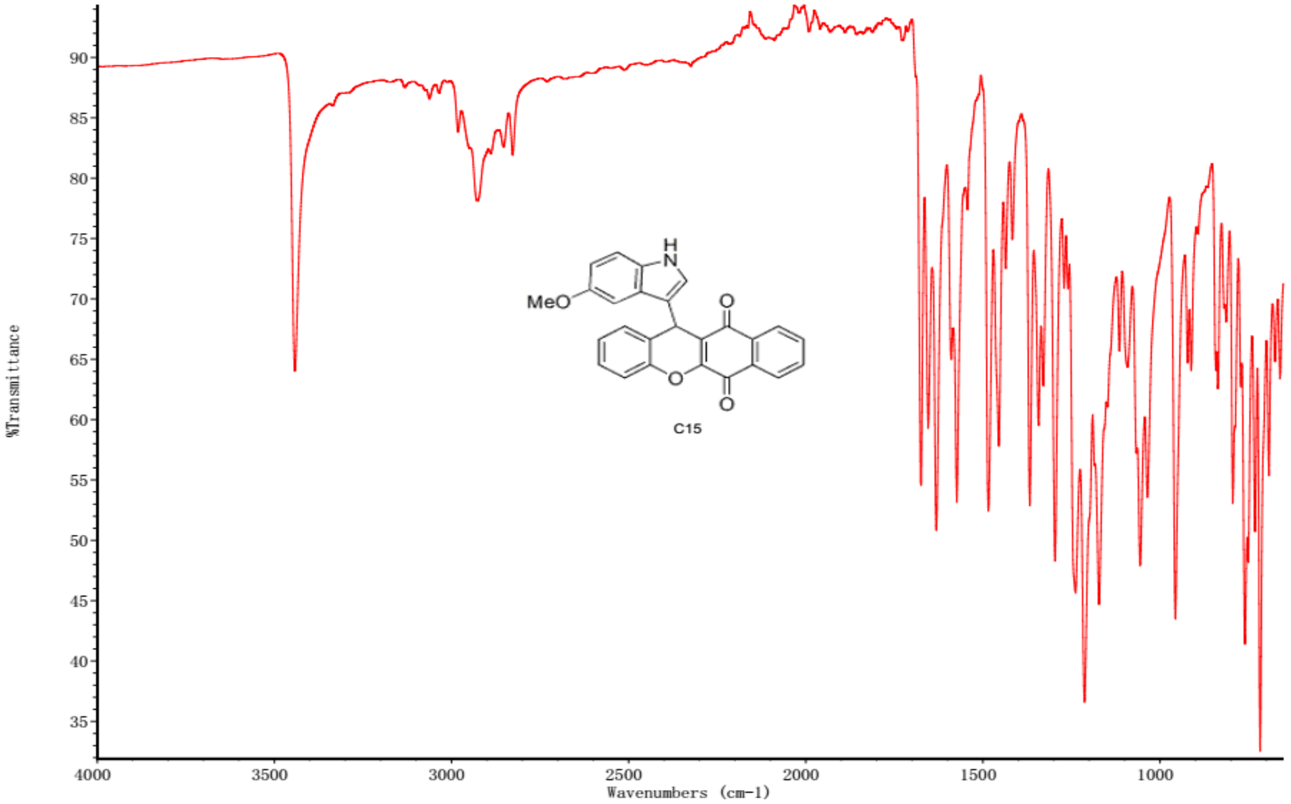

Figure S66 IR of **4q**

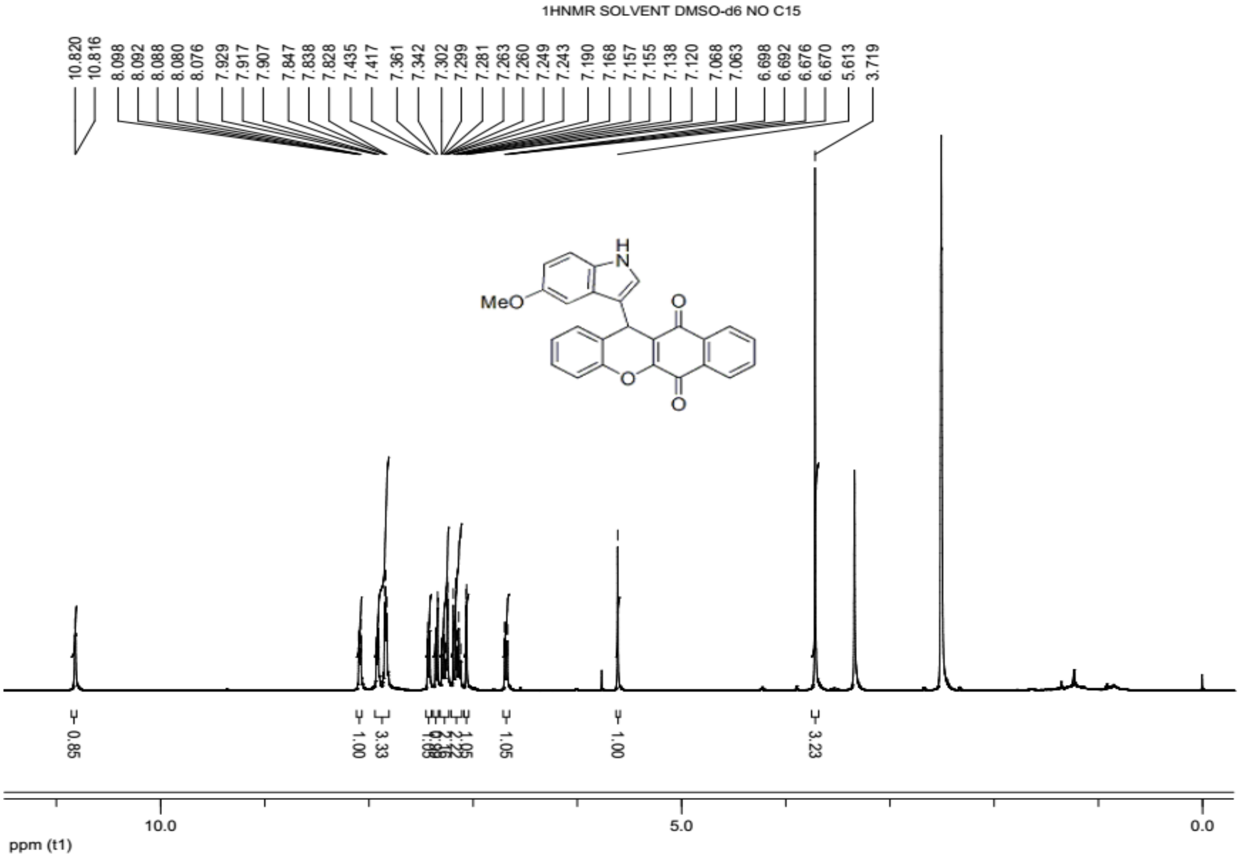

Figure S67 [I]H NMR of **4q**

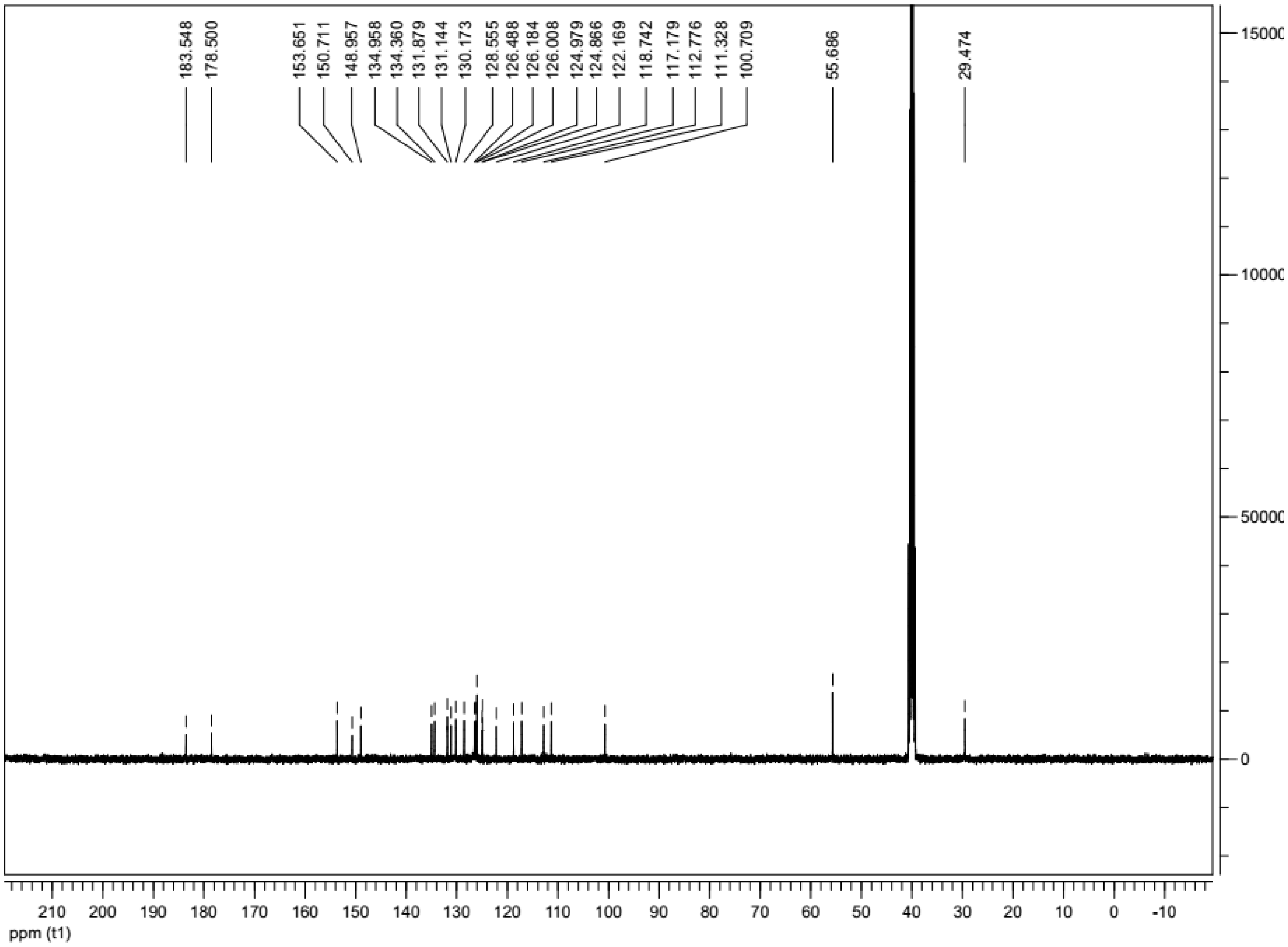

Figure S68 ^{13}C NMR of **4q**

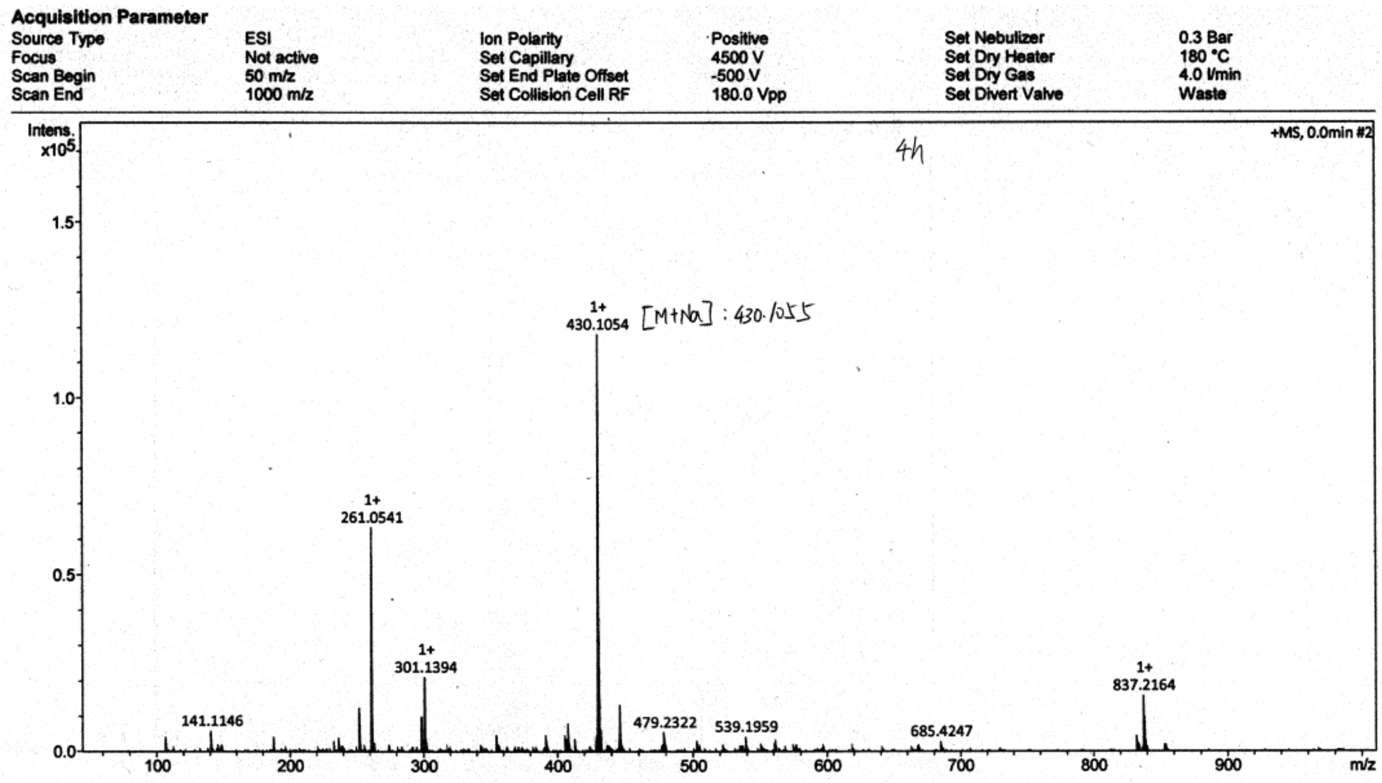

Figure S69 HRMS of **4r**

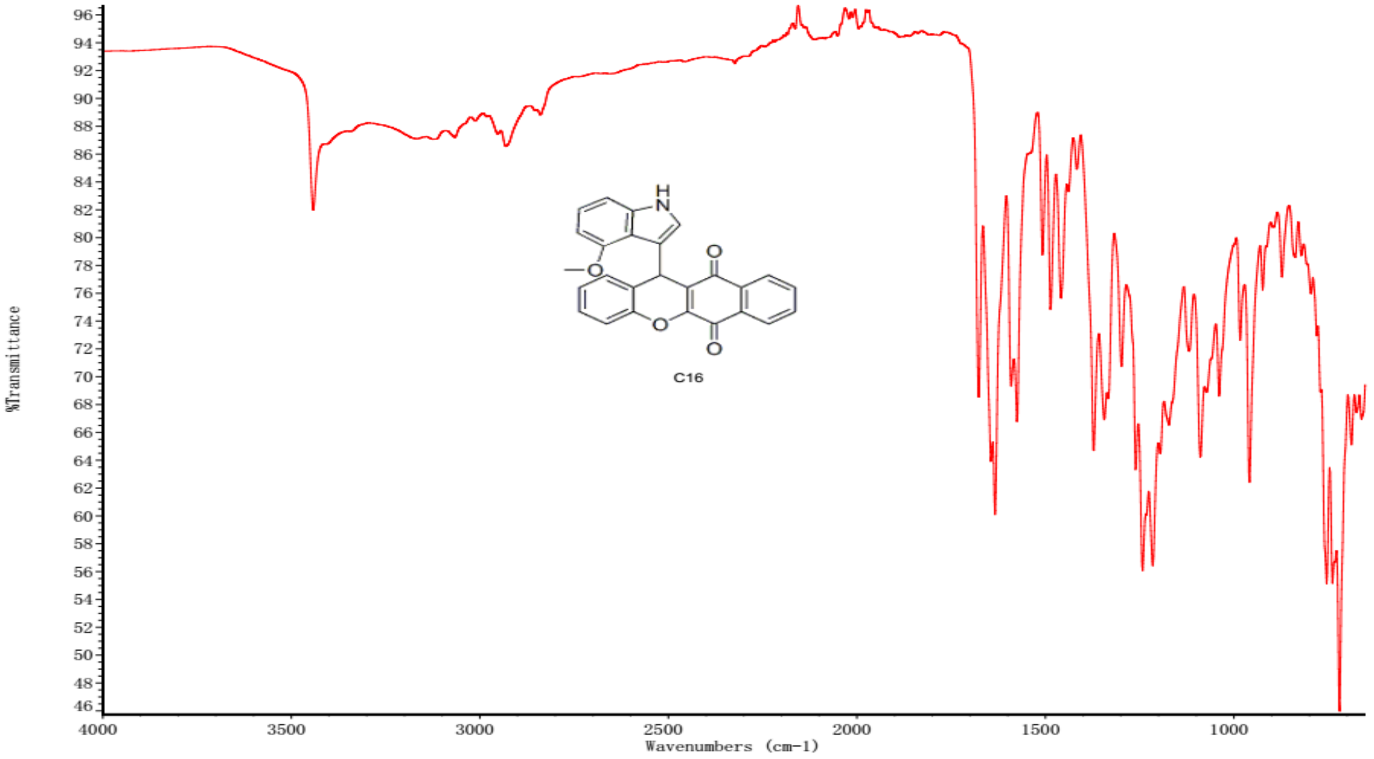

Figure S70 IR of **4r**

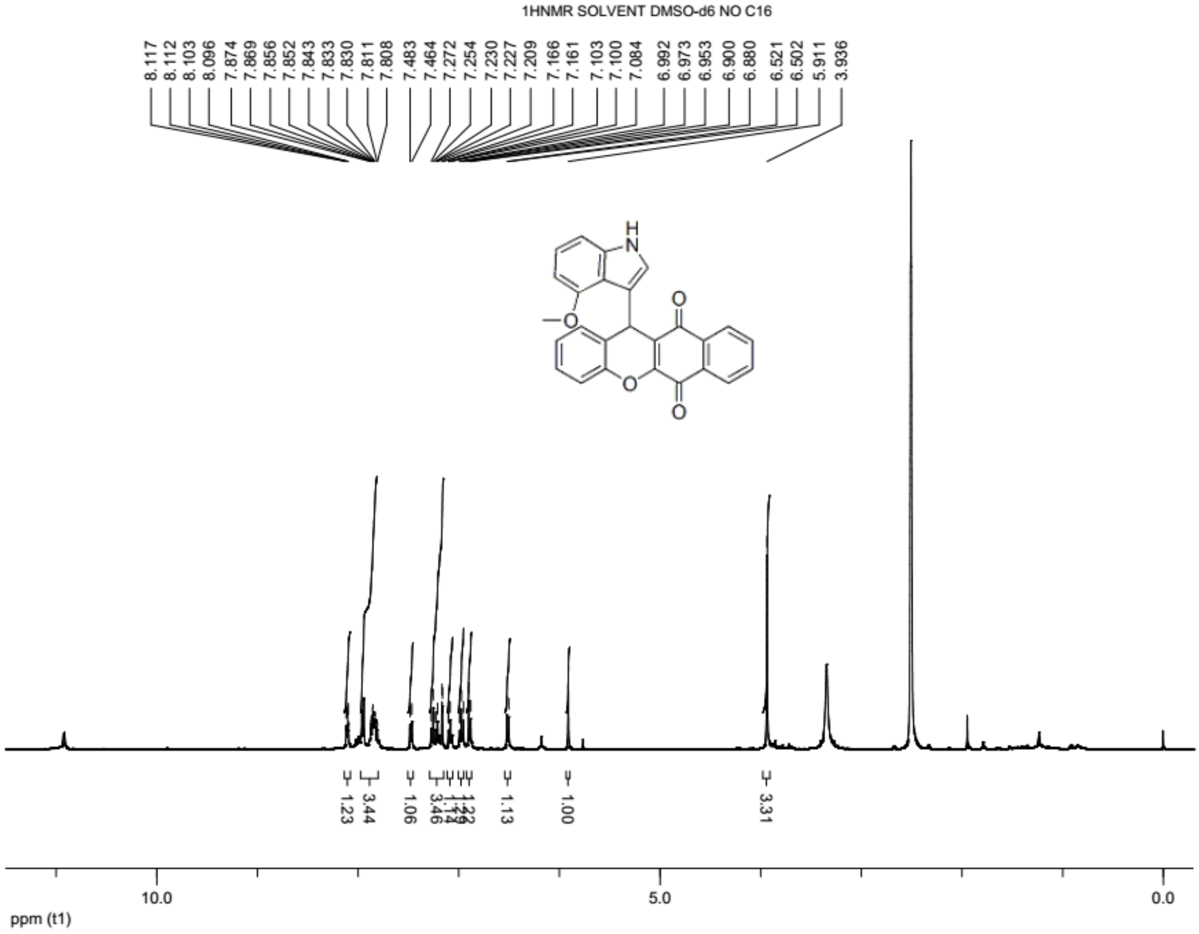

Figure S71 ^{1}H NMR of **4r**

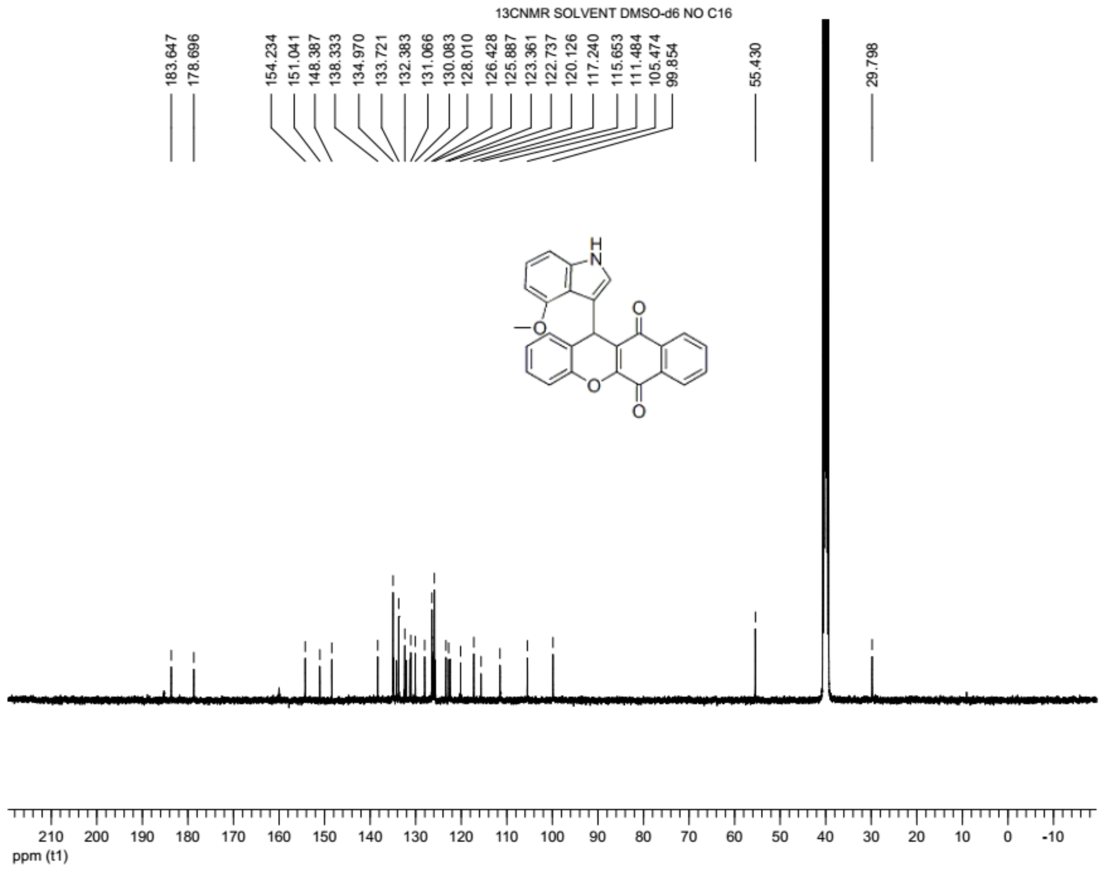

Figure S72 ^{13}C NMR of **4r**

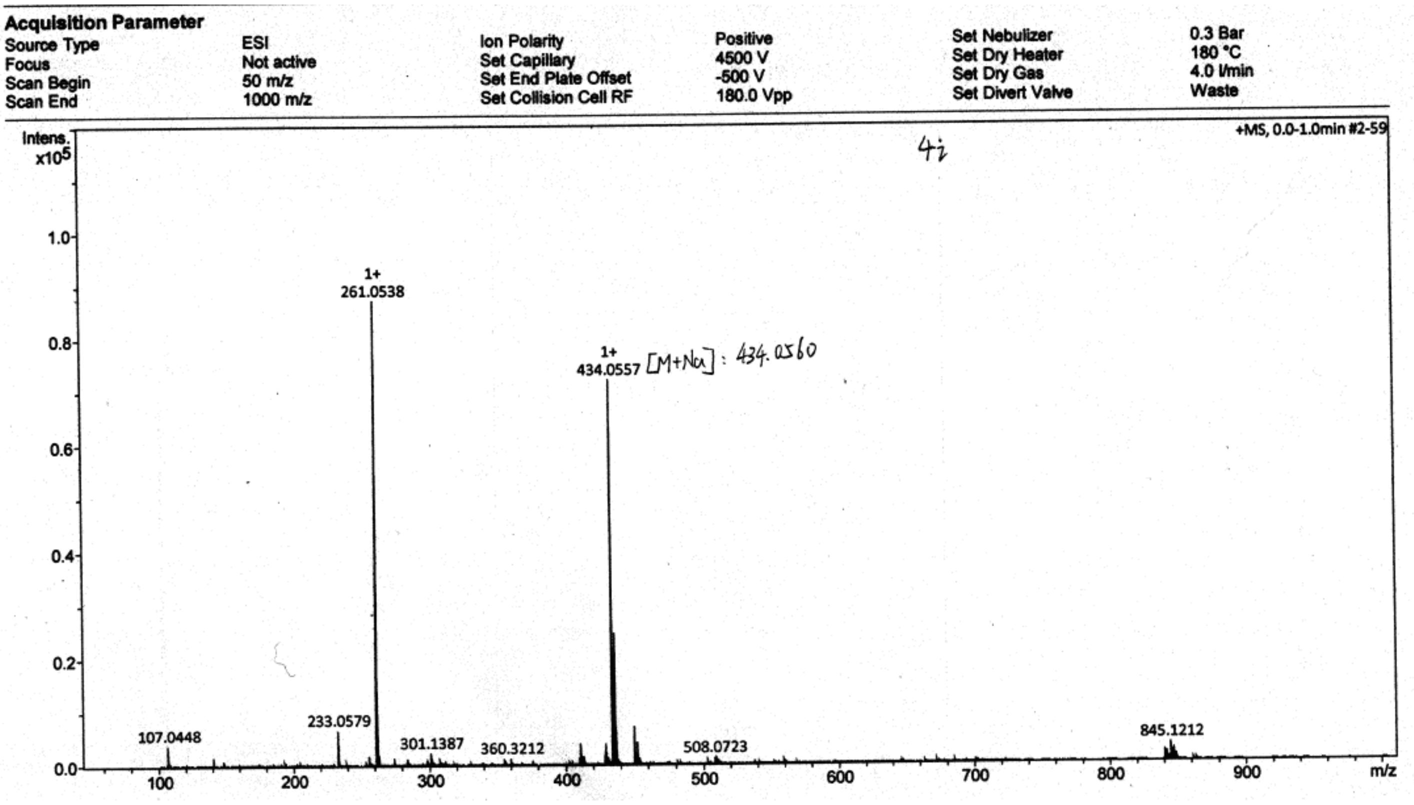

Figure S73 HMRS of **4s**

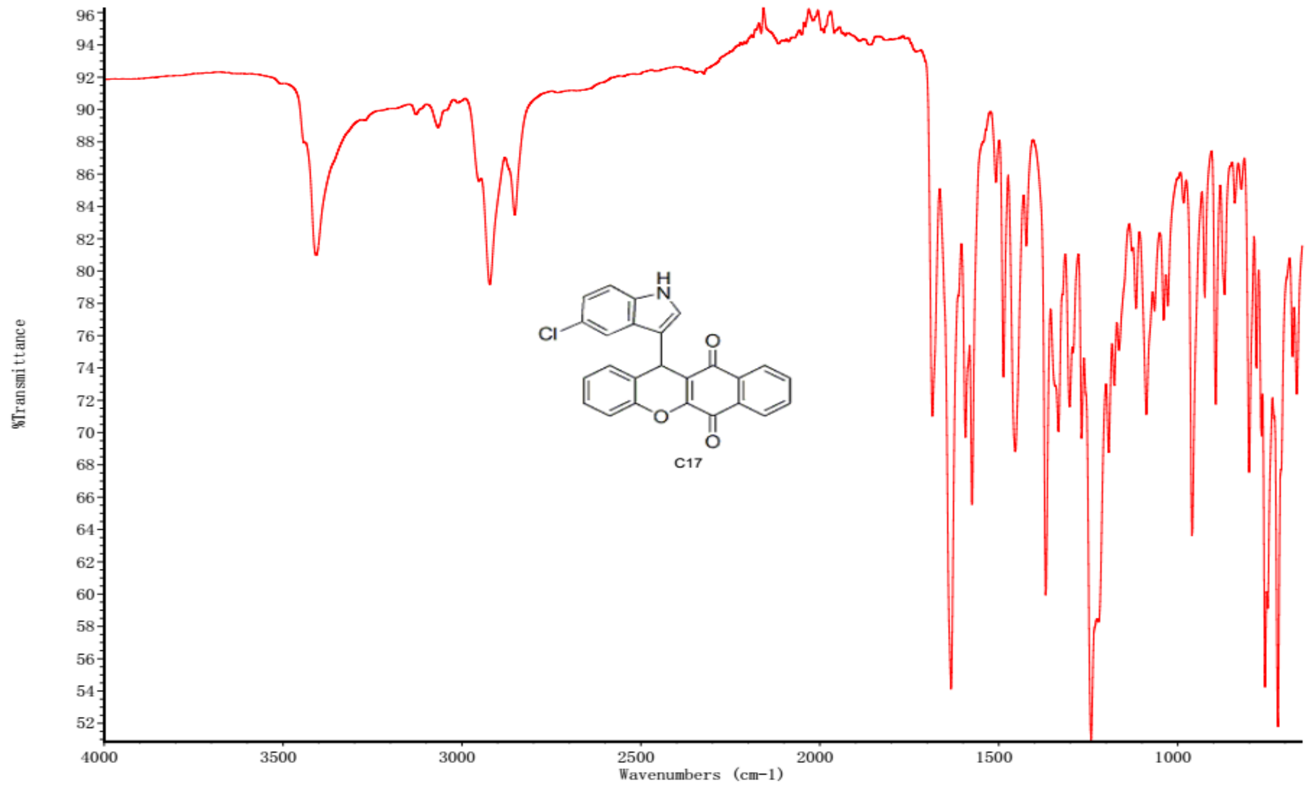

Figure S74 IR of **4s**

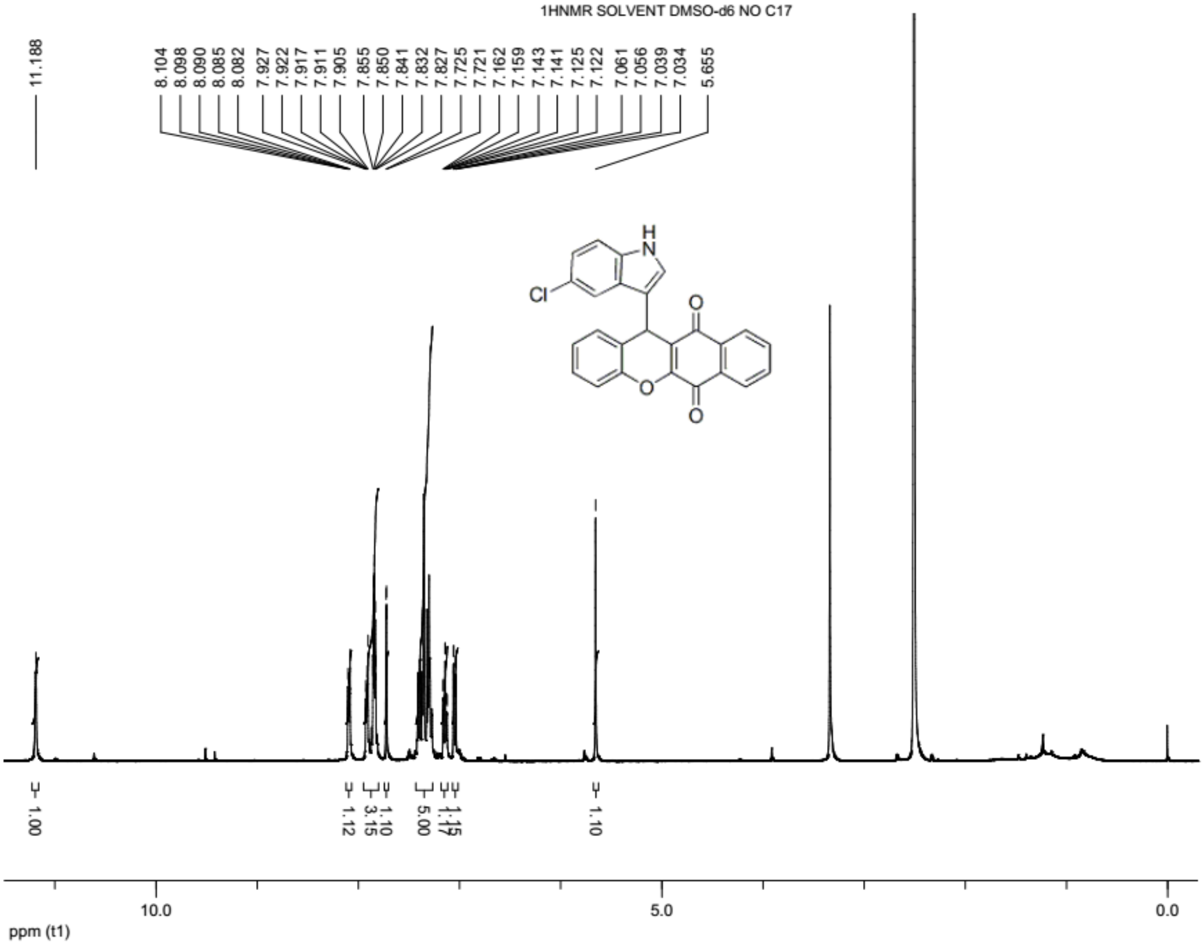

Figure S75 ^{1}H NMR of **4s**

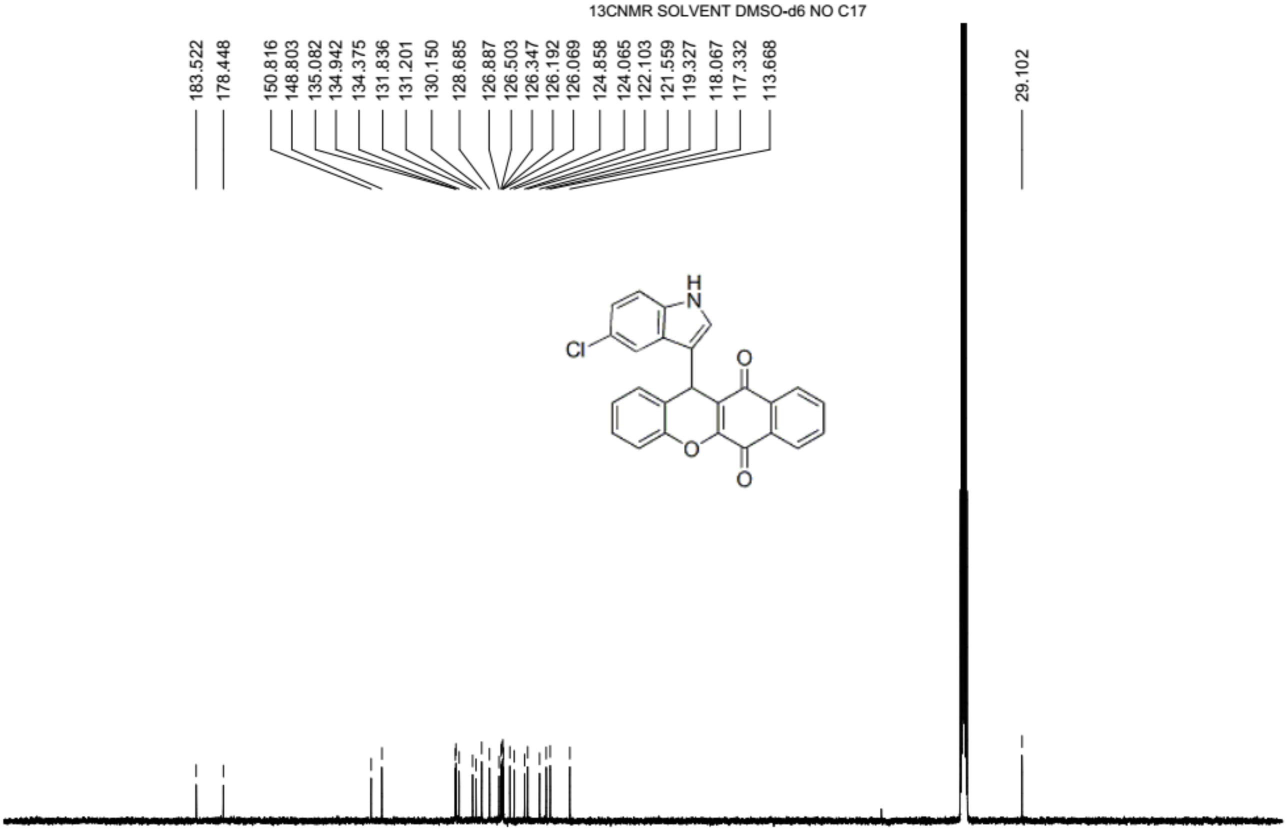

Figure S76 ^{13}C NMR of **4s**
